# Impact of Digital Contact Tracing and Other Nonpharmaceutical Interventions on Pandemic Control: Microlevel, Behavior-Driven Agent-Based Model Analysis

**DOI:** 10.2196/87527

**Published:** 2026-09-30

**Authors:** Luis Ignacio Lopera Gonzalez, Göran Köber, Göran Kirchner, Justus Benzler, Oliver Amft

**Affiliations:** 1 Chair of Digital Health, Friedrich-Alexander-Universität Erlangen-Nürnberg, Erlangen, Germany; 2 Intelligent Embedded Systems Lab, Department of Computer Science, University of Freiburg, Georges-Köhler-Allee 304, Freiburg, 79110, Germany, 49 761-887-865733; 3 Infectious Disease Epidemiology, Robert Koch Institute, Berlin, Germany; 4 Hahn-Schickard gGmbH, Freiburg, Germany

**Keywords:** exposure notification, multi-shot pathogen modelling, human behavior modelling, agent-based modelling, nonpharmaceutical intervention simulation

## Abstract

**Background:**

Nonpharmaceutical interventions (NPIs), including digital contact tracing (DCT), are central to control the spread of airborne pathogens. Nevertheless, the effectiveness of individual interventions and the role of personal behavior remain insufficiently understood.

**Objective:**

This study aimed to understand how NPI combination and the individual’s behavior contribute to reducing airborne pathogen transmission.

**Methods:**

We disentangle the efficacy of individual NPIs, including DCT, with a novel microlevel, behavior-driven agent-based model (ABM) that simulates individual human behavior to analyze the effectiveness of nonpharmaceutical interventions on airborne pathogen propagation. Our model’s Zeitgeber architecture delineates contextual characteristics, including daytime, daily routines, locations, and activities. Our method determines each agent’s current location and behavior in a realistic environment under NPI restrictions. We model viral load transfer between agents from contact duration, distance, and the infected agent’s infectiousness level. We examine the effects of DCT-related behavior parameters, including adoption, adherence, and compliance, with a default intervention, and with further restricting and relaxing NPIs, on key pandemic indicators.

**Results:**

The effect analysis of personal choices regarding DCT indicates that a high DCT adoption rate (activate DCT) should be the first goal of a DCT implementation campaign, followed by adherence (notify others), and compliance (follow recommendations, if notified). There is no monotonic path in the behavior parameter space to improve pandemic characteristics. Assuming realistic behavior with regard to DCT, total infections were reduced by 43% in the default intervention, while DCT combined with other NPIs reduced total infections by up to 52%. Surprisingly, however, some restricting NPI combinations do not improve pandemic characteristics.

**Conclusions:**

DCT implementations will face challenges, as pandemic characteristics do not consistently improve when behavior parameters ( adoption, adherence, and compliance) are increased. When considering realistic behavior, more is not always better; NPI combinations can interfere with each other to the detriment of pandemic control. Our approach offers fine-grained insight on the effectiveness of NPI combinations that cannot be obtained in human studies due to confounding effects. Thus, our approach can guide future pandemic control efforts and prioritization for pandemic preparedness.

## Introduction

Contact tracing is a widely acknowledged tool for outbreak control, especially of human-to-human transmitted respiratory diseases, including pandemic ones. Manual contact person tracing (MCT) is the standard procedure to date, where health authorities perform recall interviews to determine an infected individual’s contact persons, notify those contact persons (mostly by phone) regarding their exposure, and instruct them to quarantine. Upon the global outbreak of coronavirus SARS-CoV-2, health authorities worldwide introduced digital contact tracing (DCT) apps for the first time to help end infection chains. For example, in 2021, TraceTogether (Government Technology Agency; a DCT app administered in an opt-out scheme) was used by more than 90% of Singaporeans [1].

By design, DCT could complement the comparably slow, laborious, error-prone, and not easily scalable process of MCT [2]. Instead of health authorities tracing contact persons, smartphone DCT apps leverage the device’s autonomous monitoring and analysis of Bluetooth signal attenuation received from other nearby smartphones [3]. Consequently, DCT apps can estimate contact duration and distance to other smartphones. DCT focuses on individuals at risk rather than the general population by notifying them about their exposure. As the methods of exposure notification differ between MCT and DCT, the behavior change implementation is different too. For MCT, the exposure notification is followed by an instruction of the health authority, whereas DCT apps provide a recommendation only, and no authority holds the anonymous individual accountable. DCT could avoid more restrictive nonpharmaceutical interventions (NPIs), for example, lockdowns and generalized strict social distancing [4]. In contrast to other NPIs, DCT could thus be sustainable over years [5].

Several studies and reviews have explored the effectiveness of DCT [3-15], with experimental results showing mixed outcomes, ranging from effective [3,12,13] to ineffective [7,14]. However, evaluating the impact of DCT empirically with observational data is infeasible due to overlapping NPI effects during the COVID-19 pandemic. Various approaches have been proposed to model and simulate contact tracing mechanisms in a pandemic. Compartmental models are used to model the pandemic continuum based on estimated epidemiological parameters [16,17]. Usually, compartmental models presuppose homogeneity [18], that is, people are interchangeable. However, from an epidemiological perspective, the unique social behavior of individuals significantly influences pathogen transmission [19]. In this context, the compliance of highly interconnected individuals (ie, potential superspreaders) with NPIs is more critical than that of individuals with fewer social connections [20]. Thus, comprehensive individual-level simulations are warranted and may help to identify optimal combinations of DCT and other NPIs for pandemic preparedness.

Agent-based models (ABMs) are ideal for individual-level simulations as they can encode human behavior heterogeneity using micro- and meso-level agent descriptions. Microlevel descriptions represent individual agent behavior [21], while meso-level descriptions statistically summarize group behavior [22]. Moreover, meso-level ABMs [11,23-25] allow modelers to specify human interaction at the level of social arrangements using contact networks, including contact patterns by location, for example, home or office. In contact networks, nodes represent individuals, while the network edges represent their interaction. NPIs are encoded as changes in the network topology, either by removing edges or by changing edge transmission probabilities before the agent simulation [10]. As the contact networks determine agent interaction, they control the probability of pathogen transmission [11]. In other words, meso-level ABMs summarize interactions between individuals by abstracting the diversity of individual interactions (eg, contact duration) and their dynamic character (eg, contacts due to individual behavior patterns). While the abstraction of interactions in meso-level ABMs reduces simulation complexity, the effect of an NPI on individual interactions needs to be known or hypothesized to modify the ABM’s contact networks [26]. Thus, the analysis of NPI combinations with meso-level ABMs tends to be vague. In contrast, microlevel ABMs can represent NPIs as a change of the behavior rule set that drives agents; therefore, they are ideal to capture the emergent effect of NPIs on pathogen transmission [25,27].

In this paper, we propose a microlevel, behavior-driven ABM to capture the combined influence of individual human behavior on the capacity of NPI combinations, including DCT, to control pathogen transmission. Here, we refer to individuals as specifically created agent models that mimic natural human behavior in the ABM simulation. We use the term agent when referring to the technical implementation. Specifically, the contributions of this work are as follows:

1. We present the design of a behavior-driven ABM model to analyze DCT and NPI combinations as well as its calibration and validation. We demonstrate how the emerging properties of the model simulation match data from literature on activity patterns, contact patterns, and COVID-19 epidemiological observations.
2. We analyze compliance, adherence, and adoption behavior regarding DCT’s capacity to control the pandemic. We show that the path to improve pandemic indicators is not linear and discuss how policymakers may benefit from the findings to improve NPI rollout.
3. We analyze how NPI combinations and individual behavior interact to control the pandemic. We show that under realistic behavior assumptions, NPI effects may not be additive, and we propose future research to better understand the mechanisms.

## Methods

### Overview

ABMs serve as a pivotal tool for unraveling hypothetical outcomes in complex systems. Here, we investigated the specific and joint effect of DCT and other NPIs on pandemic scenarios, which are governed by contacts between people. A component-based model architecture facilitates our approach, which encapsulates individually validated, realistic properties. The architecture builds on 3 model layers: behavior, pathogen, and NPI. To delve deeper into our model’s framework, encompassing its entities, parameters, and initial states, we guide readers to the ABM overview, design concepts, and details (ODD) [28,29] protocol (Multimedia Appendix 1) and Multimedia Appendix 2. Specifics of the modeling and simulation platform can be found in our Python-based implementation, which is publicly accessible on GitHub [30]. Figure 1 provides an overview of the Zeitgeber architecture, pathogen model, and examples of the virtual world infrastructure.

Behavior drives agents to engage in various interactions within their social networks of family, friends, and acquaintances, and experience encounters outside these circles, thus providing additional risk of contracting or transmitting the virus. In greater detail, we simulate agents’ activity patterns, including the nature and duration of activities, considering variables such as daytime, the agent’s current location, infection state (viral load level and infectiousness level). Therefore, we conceptualize NPIs as modifiers of the individual behavior that drives agent interaction, with the goal of reducing pathogen transmission. In other words, the NPI regime shapes the behavior of infected agents, their contact persons, and the population as a whole.

**Figure 1.**
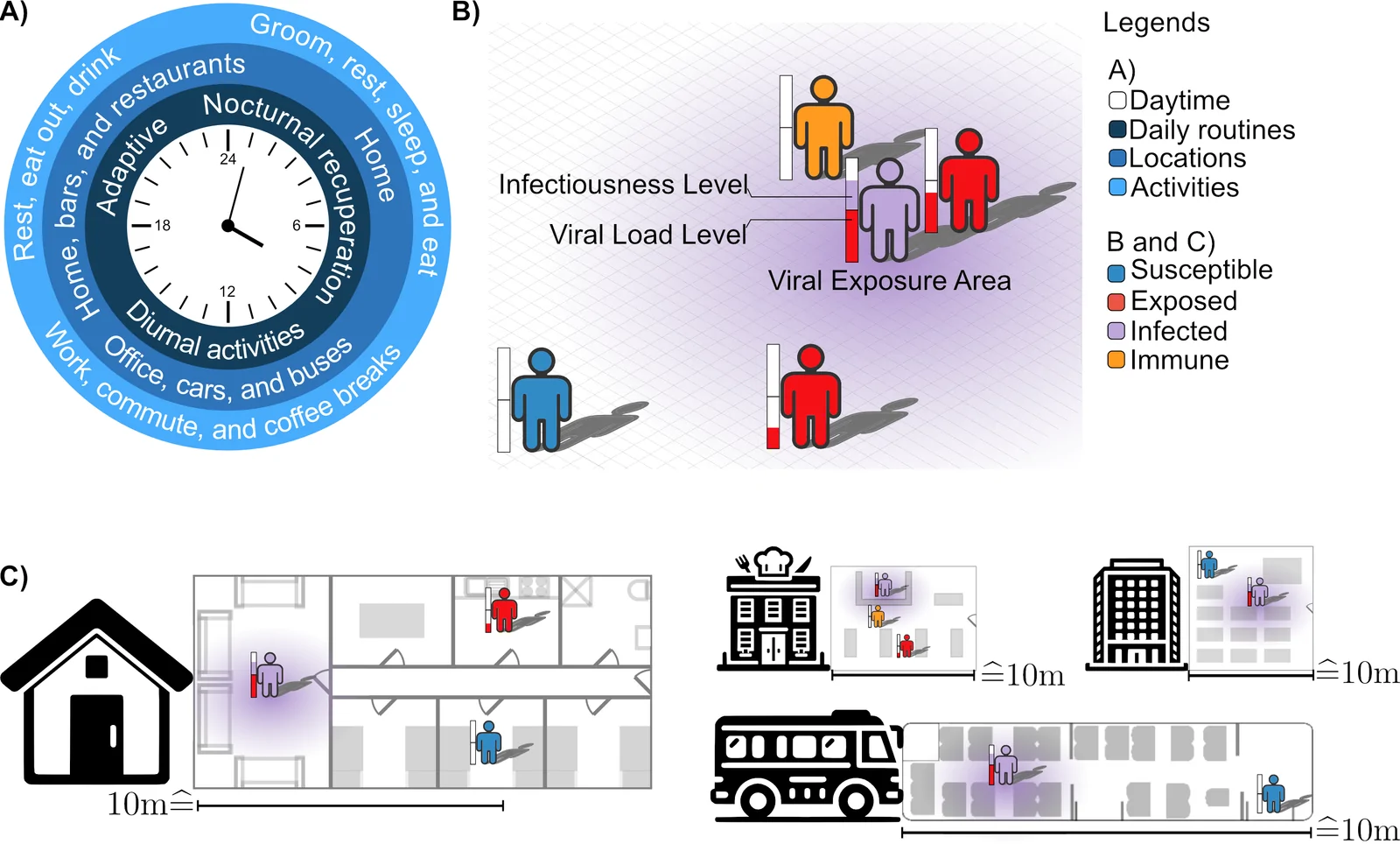
Overview of the behavior-driven ABM approach. (A) Zeitgeber architecture delineates contextual characteristics: daytime, daily routines, locations, and activities. Simulation timekeeping controls the agents’ locations and location-based activities that result in contacts (see Figure 2 for details). (B) The pathogen model describes an agent-specific, dynamically accumulated viral load level due to contacts, their duration, and distance between susceptible and infectious agents and a time-dependent viral removal. The transition from exposed to infected (ie, self-sustaining viral state) is denoted by the viral load threshold (see Figure 3 for disease states and characteristics). An infected agent’s time-dependent infectiousness level determines the exhaled viral load. (C) Snapshots of virtual world infrastructure and agent instances, illustrating the spatial and dynamic character of viral load level (susceptible agents) and viral exposure area (infected agents). Details of all submodels can be found in the Methods section. An example simulation video can be found in Multimedia Appendix 3.

**Figure 2.**
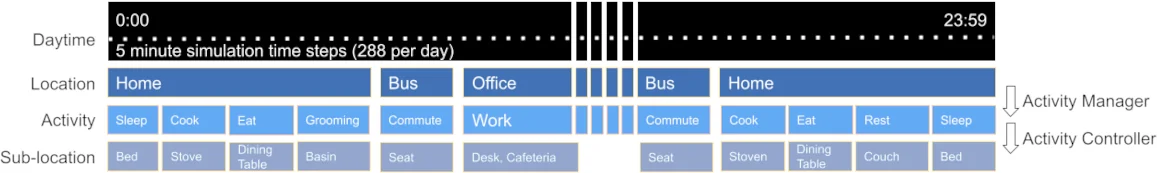
Example of a single-day activity trace illustrating the Zeitgeber architecture’s three contextual characteristics (daytime, location, and activity). In our behavior model, the Zeitgeber schedule determines location, the activity manager orchestrates activities represented by activity controllers, and activity controllers determine individual activities and their sublocation.

**Figure 3.**
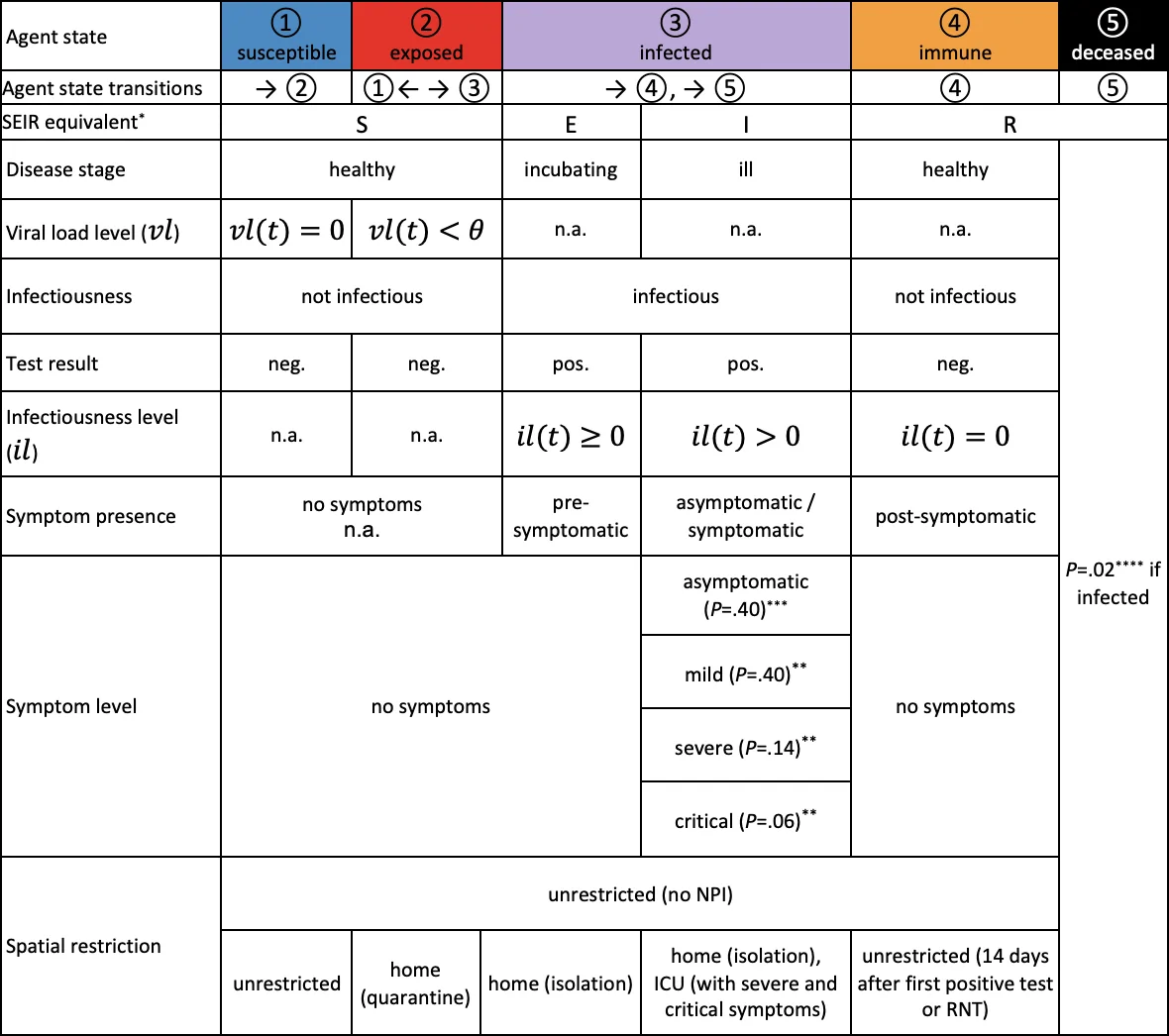
Temporal association of key terminology and pathogen model concepts. The temporal component is driven by the Agent state. *Agent state equivalence to classical susceptible, exposed, infected, and recovered (SEIR) models [31]. **Symptom severity distribution based on WHO data [32]. ***Asymptomatic rate [33,34]. ****Death rate extracted from Johns Hopkins dashboard [35].

### Zeitgeber Architecture

#### Overview

In our approach, agent behavior is intricately shaped by various factors, including the cyclic nature of daily routines, environmental contexts, and intrinsic personal necessities [36]. Thus, behavior plays a pivotal role in guiding the agents as they navigate through the virtual world.

#### Zeitgeber System

While developed in the field of chronobiology to explain exogenous cues that influence the biological inner clocks of organisms [37], the Zeitgeber concept has been extended and applied to various environmental factors, including physical activity and social interaction [38-40]. To realistically represent the daily life patterns of individuals, we simulate Zeitgeber that orchestrate exogenous cues across 3 daily routines, each representing a segment of a typical day (refer to Figure 1A): *diurnal activity* encapsulates a structured array of work-related activities, including commuting, office work, and work breaks. *Adaptive* is a daily routine with increased flexibility and adaptability, where agents may engage in evening activities, ranging from staying at home to social outings. *Nocturnal recuperation* is dedicated to rest and recovery, essential for resetting the agents’ internal biological clocks. Each daily routine dictates agent location and sets the stage for their scheduled, reactive, and opportunistic activities [41]. For example, a scheduled activity (here equivalent to location-dependent default activity) is office work. A reactive activity is, for example, to quarantine upon being requested by a contact tracing notification. Opportunistic activities are suggested by a situation or environment, for example, to cook in a kitchen. The Zeitgeber architecture improves over previous behavior representations by combining context-aware behavior models with variable schedules and by adapting behavior to changes in the scenario, for example, using circadian rhythms to determine sleep schedules when at home, starting the office commute at different hours, and staying home when restaurants are not available. To transfer between locations, agents schedule their commute, in particular, to transfer between work and home locations. For an exhaustive list of activities, along with their associated environments and types, we direct readers to the Table S1 in Multimedia Appendix 1. The hierarchical structure of the Zeitgeber and the layers that synthesize agent activities are depicted in Figure 2.

#### Activity Manager

The *activity manager* orchestrates activities at the agent’s current location based on daily routine and location determined by the Zeitgeber. When an agent arrives at a location, different effects influence their engagement in activities. First, intrinsic necessities represent an agent’s internal drive to undertake specific activities when feasible, for example, to sleep when at home [42]. Second, available facilities, for example, beds, stoves, and so on, provide the basis for an agent’s interactions with their surroundings. For each location type, an activity list and required facilities were predefined. From an implementation perspective, the activity manager polls and prioritizes location-based *activity controllers* that model an activity and its required facilities. By choice of design, NPIs were implemented as changes in schedule and location availability.

#### Activity Controllers

Location-specific *activity controllers* are used to determine activity patterns, ie, onset and duration of an activity, its interruptibility by other *activity controllers*, as well as a delay (ie, cool-down period) before repeating an activity. *Activity controllers* encode knowledge about necessary objects and facilities as well as required motion patterns for the activity; thus, they can simulate intricate movements and interactions. For example, the *activity controller* for cooking requires kitchen furniture, and the controller for sleeping requires a bed.

A more elaborate example, when the Zeitgeber determines “home” as location, the *activity manager* polls all *activity controllers* of the home and may determine the agent to initiate cooking. The *activity manager* derives activity information from the corresponding *activity controller* for cooking, including moving the agent into the sublocation kitchen, positioning the agent appropriately in front of one or more kitchen appliances, etc.

For the *activity manager’s* orchestration of activities, sleep has the highest priority. For sleep at the agent’s home, sleep patterns are derived according to circadian distributions for sleep duration and sleep midpoint. If no other *activity controller* provides an activity during wake hours, the opportunistic activity (ie, default activity) of the location is chosen. Thus, every agent receives an activity at all times during the simulation.

### Pathogen Model

#### Overview

The pathogen model describes the dynamics of a respiratory disease in a spatial agent simulation. We used 4 established concepts to describe the viral load dynamics [39-43]: infectiousness level, viral load transfer, viral load threshold, and viral load removal. Our model represents processes of inhaling and exhaling pathogen particles, transferring viral particles between individuals [44], and body responses to viral particles. Our basic modeling concept is that an infectious agent exhales viral particles proportional to their infectiousness level [45]. Furthermore, a susceptible agent can acquire exhaled particles depending on the distance to an infected agent and the duration of the contact [43]. By inhaling viral particles, a susceptible agent becomes exposed [46,47], and the following rules apply: if no further viral particles are acquired through contacts, any previously accumulated viral particles get removed over time [47,48], and the agent finally becomes susceptible again. Alternatively, acquired viral particles may accumulate faster than they get removed. Consequently, a viral load threshold is reached [49], upon which the agent becomes infected and enters the virus incubation phase. Our pathogen model aligns with evidence that frequent, enduring, and close exposures are more likely to lead to infection [50]. The model behavior described above is known as multishot [51] viral load transfer.

While in susceptible and exposed agent states, viral load level *vl* determines state transitions, and during the infected state, infectiousness level *il* determines when an agent transitions out of the infected state. Furthermore, *il* determines how much viral load is exhaled. The infectiousness level *il* of an infected agent rises and falls as a function of their biology, possibly experiencing symptoms [52]. Upon exiting the infected state, 1 of 2 follow-up states is attributed to an agent with a probability of 0.98 for immune and of 0.02 for deceased.

For details, refer to the Pathogen submodel section in Multimedia Appendix 1. Figure 3 offers a comprehensive overview of the key pathogen concepts and their relations throughout the course of an infection. The model integrates empirical data to represent pathogen spread and disease progression (eg, viral load threshold and incubation period). Disease progression of infected agents is tracked in the simulation to assess NPI effectiveness.

#### Infectiousness Level

$il_{j}(t)$ is the normalized infectiousness level of the j-th agent at time t. The infectious level determines the transmittable viral load to a susceptible or exposed agent (Equation 1). Technically, we use triangular functions to approximate $il_{j}(t)$. For each infectious agent j, an incubation period $\tau_{j}^{i}$, a clearance period $\tau_{j}^{c}$ (Table 1), and a peak infectiousness level ${il}_{j}^{p}$ are sampled from the truncated normal distributions $N(\mu^{i},\sigma_{var}^{i})$, $N(\mu^{c},\sigma_{var}^{c})$, and $N(\mu^{p},\sigma_{var}^{p})$, resulting in a unique triangular function per infectious agent j called the infectiousness level profile. The function for the j-th agent is given by Equation 1, where $t_{j}^{0}$ is the infection time for the j-th agent.


(1) $$il_{j}(t)=\left\{ \begin{array}{ll} il_{j}^{p}\cdot \frac{t-t_{j}^{0}}{\tau_{j}^{i}}, & \text{if }t_{j}^{0}\leq t§lt;t_{j}^{0}+\tau_{j}^{i},\\ il_{j}^{p}\cdot \left(1-\frac{t-t_{j}^{0}}{\tau_{j}^{c}}\right), & \text{if }t_{j}^{0}+\tau_{j}^{i}\leq t§lt;t_{j}^{0}+\tau_{j}^{i}+\tau_{j}^{c},\\ 0, & \text{otherwise}. \end{array}\right.$$

The normalization factor and distribution parameters are based on empirical data [53] and provided in Multimedia Appendix 1. We provide an exemplary visualization of 50 sampled infectiousness level profiles in Figure 4A; for visual convenience, we centered all infectiousness level profiles at the time point of symptom onset here. Symptom onset $t_{j}^{s}$ was defined heuristically as the time point that cuts the area under the triangle function $il_{j}(t)$ in half [53,54].

**Table 1.** Pandemic characteristics are defined as emergent quantities measured on the virtual population. The pandemic characteristics are used to validate and measure the effect of the simulated NPIs.

Group and metric	Definition
Pathogen	
Clearance period	Duration in days between onset and cessation of symptoms of infected agents. For asymptomatic agents, we derived the theoretical onset and cessation of symptoms from their infectiousness level curve.
Incubation period	Duration in days between the infection time and the onset of symptoms. For asymptomatic agents, we derived the theoretical onset of symptoms from their infectiousness level curve.
Generation interval	Duration in days between the infection time of an infector and the infection time of the infectee.
Serial interval	Duration in days between the symptom onset of an infector and the symptom onset of the infectee. If the person remains asymptomatic, the theoretical symptom onset is used.
Population	
Total infections	Number of infections throughout the simulation run. Given that in our simulations every agent can be infected only once, total infections are equivalent to the number of agents infected.
7-day hospitalization incidence	The maximum number of agents per 100,000 population per run admitted to hospital due to the pandemic pathogen within a rolling 7-day time period.
7-day incidence	The maximum number of agents per 100,000 population per run testing positive for the pandemic pathogen within a rolling 7-day time period.
Wave duration	Duration in days measured from the time point of infection of the first infected agent (case 0) to the latest time point of recovery of any infected agent.
False discovery rate (of confinement)	The ratio of the total number of time units (eg, days) of agents in quarantine (false positives: noninfectious, but confined) over the total number of time units of all confined agents (predicted positives), ie, in quarantine or in isolation.
False negative rate (of confinement)	The ratio of the total number of time units (eg, days) of infected agents that are not isolated (false negatives: infectious, but not confined, ie, asymptomatic or noncompliant) over the total number of time units of all infectious agents (positives).
Agent	
Days in quarantine	The cumulated duration in days that a susceptible or exposed agent is confined at home according to quarantine rules after receiving a contact notification through any contact tracing method, either until the time point of infection or until the quarantine end condition is met. Applies only to agents who have been quarantined at least once.
Days in isolation	The cumulated duration in days that an agent is confined at home between the time point of infection and the time point of recovery. Applies only to agents who have been isolated at least once.

**Figure 4.**
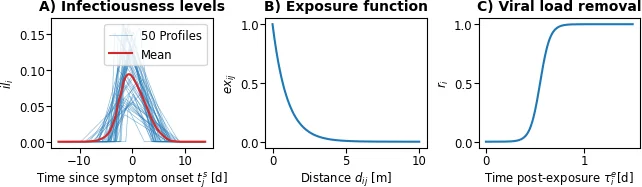
Key functions of the pathogen model. (A) Infectiousness level profiles of 50 exemplary agents, sampled from empirical data [53]. The profiles are shown relative to the agent’s symptom onset. (B) Viral exposure function to represent the distance-dependent dilution of viral load transmitted from agent j to agent i (Equation 1). (C) Clearance, that is, time-dependent viral load removal, to represent the reduction of viral load through viral decay and the agent’s immune system.

#### Viral Load Level

The viral load level $vl_{i}(t)$ of an agent i denotes the normalized accumulated pathogen particles at time t. Agents may accumulate viral load by inhaling pathogen particles when in contact with infectious agents.

The viral load, transferred from infectious agent j to susceptible agent i is modeled according to the viral exposure function ${ex}_{ij}(d_{ij})=e^{-d_{ij}}$, where $d_{ij}$ is the Euclidean distance between agents i and j (Figure 4B). The exposure function is a model parameter. The function was selected because it adjusts to droplet risk estimations [55,56], and has no singularities at close distances (Figure 4B). We calibrated the model with empirical data based on the large aerosol droplet propagation distance generated while speaking [57,58].

The viral load level ($vl_{i}$) of a healthy agent i at simulation time t depends on the viral load level of the previous simulation time step t-1, the newly received viral load, and the viral load removal according to $r_{i}(\tau_{i}^{e})$, if there were no new encounters with infectious agents. The viral load transfer is determined as presented in Equation 2.


(2) $$vl_{i}\left(t\right)=vl_{i}\left(t-1\right)\cdot \left(1-r_{i}\left(\tau_{i}^{e}\right)\right)+\sum_{j=1}^{J}ef\cdot {il}_{j}\left(t\right)\cdot ex_{ij}\left(d_{ij}\right)$$

By design, the infectiousness level $il_{j}(t)$ reflects the size of the viral population inside the host. By using the serial and generation intervals, we calibrated the exhaled viral load factor *ef* to ⅙. Please refer to Multimedia Appendix 2 for details on our empirical calibration.

#### Viral Load Removal

The clearance mechanism is essential to represent the immune system’s function and natural virus decay in reducing the previously accumulated normalized viral load level. We model the process by introducing the viral load removal of agent i depending on the elapsed time since the last exposure ($\tau_{i}^{e}$, measured in days) as presented in Equation 3.


(3) $$r_{i}\left(\tau_{i}^{e}\right)=\sigma_{sig}\left(20\cdot \left(\tau_{i}^{e}-0.55\right)\right)$$

where $\sigma_{sig}$ denotes the sigmoid function (Figure 4C). The viral load removal is based on a transmission case [59], where short, multiexposures, within 24 hours, led to the redefinition of a “close contact.” The function’s constants were calibrated by measuring the emergent serial and generation intervals.

#### Viral Load Threshold

A viral load threshold θ controls the transition from exposed to infected state. When an agent’s viral load level $vl_{i}(t)$ accumulates to the viral load threshold θ, the virus presence is considered self-sustained and no longer depends on accumulation and removal dynamics. When $vl_{i}(t)$ exceeds θ, infection time $t_{i}^{0}=t$ is logged for the newly infectious agent i. After recovering from the disease, agents are considered immune until the end of the simulation. Therefore, for any agent i, viral load level $vl_{i}(t)$ is undefined after $t_{i}^{0}$. θ was set to 1, representing the average normalized viral load that an agent needs to transition from the exposed to the infected state. Refer to Multimedia Appendix 2 for details on the validation of the viral load threshold θ.

#### Diagnostic Tests

Testing for the virus is triggered one day after symptom onset and conducted during business hours (8 AM-4:30 PM). Agents receive test results 24 hours after the test was conducted. Agents with mild symptoms self-isolate, while severe cases are hospitalized. Positive tests are communicated through contact tracing methods. Asymptomatic agents are only tested under require negative test to exit (RNT) after being isolated by contact tracing.

#### Immunity and Mortality

During the infected agent state, there is a small probability (2%) of mortality. Deceased agents are placed in the virtual cemetery. All recovered agents are immune for the remaining simulation time.

### NPI Models

#### Overview

NPIs are conceptualized as a spectrum of actions individuals (can) take, that is, to prevent infection, including using a DCT app (refer to Textbox 1 for an introduction). Each simulated agent has a behavior profile representing the probability of undertaking specific NPI actions. NPIs are integrated as modules into the simulation framework, where they are treated as extensions of the Zeitgeber.

Textbox 1. **Nonpharmaceutical interventions (NPIs) covered in this work.**

**Restrictive NPIs**

- Restrictive NPIs modify the default intervention (see below) and prevent agents from routine activities that are considered high risk (eg, eating out).

 ***Contact tracing***

- Agents that receive an exposure notification are requested to quarantine or isolate for 14 days.
- Informal Contact person Tracing (ICT): Agents who test positive inform other (known) agents that were exposed to them. The decision to inform a contact person is based on the type of relationship with that person.
- Manual Contact person Tracing (MCT): Contact tracers interview agents who tested positive about other (known) agents that were earlier exposed to them. Contact persons are then informed based on the type of inter-agent relationships.
- Digital Contact Tracing (DCT): A proportion of the population is equipped with a DCT app that records duration and distance to other individuals equipped with the DCT app. The DCT app warns its users if there was a possible exposure to an individual who tested positive. Literature sources use “digital proximity tracing” synonymously with DCT.
  - DCT-related terminology
    - “DCT adoption”:proportion of people who downloaded and activated, for example, registered in the app and activated Bluetooth;
    - “DCT adherence”:–proportion of app users who notify others by sharing a positive test result;
    - “DCT compliance”:–proportion of app users who follow recommendations upon receiving an app notification.

 ***Other contact restrictions***

- Contact restrictions may confine (ie, quarantine or isolate) agents to their homes.
- Quarantine Contact person Household (QCH): With QCH, all household members of an agent who is identified as contact person of an infectious agent are quarantined for 14 days. RNT can shorten the quarantine period, as described above. Furthermore, QCH quarantines all household members of an infected agent.
- Closure of Bars and Restaurants (CBR): We consider bars and restaurants as public places that are inaccessible under CBR.

**Relaxing NPIs**

- Relaxing NPIs allow agents to resume routine behavior. Here we analyze RNT as a relaxing NPI.
- Require Negative Test to exit (RNT): RNT is a confinement relaxation NPI that reduces the default 14-day quarantine and isolation period by releasing agents based on a negative test. We assume that if an agent is isolated or in quarantine, the agent will get tested. For infected agents, the test happens once they recover, and for susceptible agents, the test happens at least two days after the start of the quarantine. It takes one additional day for the test to be processed and its result to be received by the agent. Upon a negative test result, agents are permitted to resume their normal activities. Thus, RNT is always used in combination with another NPI.

**Default Intervention**

- As a simulation default, all symptomatic agents will be tested, receive a positive test result, and isolate upon the positive test. The agents wait for 1.5 days after symptom onset and request the test between 8 AM and 4:30 PM; the test takes 24 hours to complete. After 14 days, the agent can resume normal behavior. In addition, we apply ICT and MCT (described above) in all simulations.

#### Contact Tracing Models

##### Overview

The objective of contact tracing technologies is to identify and isolate infectious individuals and quarantine their contact persons in order to curtail virus spread by reducing contacts between individuals. DCT has been introduced as part of the NPI portfolio in many regions at the onset of the COVID-19 pandemic. In particular, DCT is hypothesized to provide an alternative that overcomes the limitations of MCT [4]. In this study, we modeled 3 different contact tracing strategies: DCT, MCT, and informal contact person tracing (ICT; Textbox 1). In our implementation, all contact tracing strategies adhere to a uniform process flow: (1) categorize contacts, (2) construct contact networks, (3) acquire positive diagnostic tests, (4) consider informing contact persons, and (5) consider complying with recommendations. Subsequently, we provide more conceptual detail of the process flow implementation.

##### Contact Categories

Contacts are categorized at the beginning of the simulation, depending on the contact tracing strategy. For DCT, all contacts are considered equal. MCT splits contacts by location into office contacts and home contacts. For ICT, we considered family contacts (with agents from the same household), friends contacts, and acquaintances contacts, where friends and acquaintances are agents selected from the same office.

In our behavior-driven modeling, the shape and strength of the contact networks are an emerging property (Supplementary: Network Dynamics and subsequent sections in Multimedia Appendix 2). We analyze contact types by categorizing the pairwise relationships between all agents. Initially, the number of agent pairs selected for the friends category is 40% of the office size. Accordingly, in a 20-person office environment, the friends category is constructed by randomly selecting 8 agents. To construct the acquaintances’ contacts, 50% of the office-sharing agent pairs that do not belong to the friends category were selected. During simulation time, ICT considers agents as an unknown contact person if the corresponding agent does not fall within the friends and acquaintance contact persons.

##### Contact Network Construction

To construct contact networks, we consider a 3 m [57] radius of contact candidates. For DCT, the minimum continuous contact duration was set to 2 simulation time steps, that is, 10 min, before a contact within the radius is registered. For MCT and ICT, we do not apply a contact duration constraint. Thus, we support that an agent might register a contact person [60] although it was a brief encounter only.

##### Logging Duration

Regardless of the contact tracing strategy used, we log all contacts for a period of 2 simulation weeks, that is, $\tau_{log}=14days$. Contacts older than $\tau_{log}$ are considered “forgotten” and are no longer factored into the contact tracing process. The approach aligns with human memorization and record-keeping limitations in real-world contact tracing scenarios.

##### Recall Bias

Specifically for ICT and MCT, we model a recall probability to represent the temporal effects in recall performance for contacts. As a contact between agent a and b ages, the likelihood of recall declines. On the contrary, more recent contacts are more easily remembered; a phenomenon well-documented in cognitive psychology [61]. Equation 4 expresses the temporal encounter recall $P_{tr}(t,(a,b))$ between agents a and b as a function of the time since the last contact $t_{lc}$, the cumulative contact duration $\tau_{cd}(a,b)$*,* and the logging duration $\tau_{log}$.


(4) $$\begin{array}{c} P_{tr}\left(t,\left(a,b\right)\right)=\frac{\tau_{cd}\left(a,b\right)}{2\cdot \tau_{log}}\cdot \left(1-\frac{t-t_{lc}\left(a,b\right)}{\tau_{log}}\right)\\ \text{where }0\leq P_{tr}\left(t,\left(a,b\right)\right)\leq 1 \end{array}$$

If ${t-t}_{lc}§amp;gt;\tau_{log}$ the contact is forgotten and $P_{tr}(t,(a,b))=0$. The term ${1/(2\cdot \tau }_{log})$ down-weights the cumulative contact duration $\tau_{cd}$ compared with the elapsed time since the last contact $t_{lc}$.

The contextual encounter recall $P_{cr}(c,(a,b))$ represents the situational probability to recollect the contact between a and b. In MCT, c represents the location of the contact (eg, home, office, and public place). In ICT, c represents the contact category (eg, with family, friend, and acquaintance). Contacts with unknown contact persons cannot get recollected in ICT. Contextual encounter recall $P_{cr}$ was predefined per context c and can be found in the Supplementary ODD protocol in Multimedia Appendix 1. Combining both, the contextual and temporal encounter recall yields the total recall bias $P_{rb}$ (Equation 5).


(5) $$P_{rb}\left(t,c,\left(a,b\right)\right)=P_{cr}\left(c,\left(a,b\right)\right)\cdot P_{tr}\left(t,\left(a,b\right)\right)$$

##### Contact Tracing Capacity

For MCT, we model processing capacity limitations by constraining the message dissemination to an agent’s contact persons. In more detail, we simulate that agents report their infection state and a list of recalled contact persons to health authorities. Agent reports are held for 3 days to simulate MCT processing delay. Subsequently, contact person notifications are extracted from the reports and queued according to the reported time of contact. The queue is processed 1 notification per simulation time step. We simulated notification failures by applying an 80% success rate to notifying the contact person. Failed notifications are retried 2 times at most. Notifications remaining in the system that are older than 5 days are dropped from the queue. The capacity of delivering up to 288 notifications per day is equivalent to that of a typical German local health authority (Gesundheitsamt), which, at times of high incidence during the COVID-19 pandemic, was supported by 1 or 2 containment scouts for a population of 100,000 individuals [62]. In our simulations, the MCT capacity settings were applied to just 1000 agents.

##### Adoption

For a DCT app to be effective, it needs to be downloaded and activated; for example, a user registers and activates Bluetooth [63]. Adoption was implemented as the proportion of the population that “downloaded the app and activated Bluetooth,” that is, participated in DCT. Adoption was set at simulation start and remained unchanged during the simulation. For DCT, contact tracing was only possible between individuals that adopted DCT.

##### Adherence

A key success factor of contact tracing is whether individuals inform others of positive test results [64]. Adherence was implemented as the individual’s probability of reporting a positive test result within their contact networks. In our approach, the contact tracing strategy does not affect an individual’s decision to report the test result; that is, an individual who decides to adhere will inform all contact persons via DCT, MCT, and ICT. Consequently, nonadherence means that no contact tracing strategy could inform an individual of a high-risk contact.

##### Compliance With NPI Recommendations

Compliance varies across contact tracing strategies and is governed by compliance parametrization. DCT compliance [11] (Textbox 1) was varied to contrast optimal and realistic behavior (Figure 5). For MCT, we assume a uniform compliance rate of 0.95 across all agents, thus representing a general willingness to cooperate with health authorities. While MCT compliance reflects the perceived societal responsibility and trust in governmental organizations, ICT compliance varies across contact categories, acknowledging the differences in personal relationships. In particular, ICT compliance was 0.99 in case of family contacts, 0.9 for friends contacts, and 0.5 for acquaintances contacts, indicating varying levels of trust and social obligation across the contact categories.

**Figure 5.**
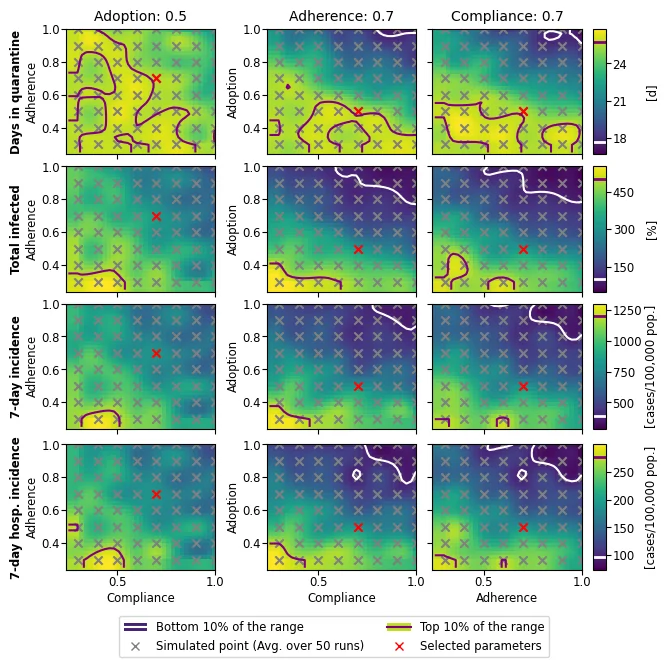
Exploration of behavior parameters: adoption, adherence, and compliance. Uniformly distributed parameter combinations were selected as simulated points. Results were averaged over 50 simulation runs per grid position. The first goal of a DCT implementation campaign should be to maximize DCT adoption (activate DCT), followed by adherence (notify others), and compliance (follow recommendations). There is no monotonic path to improve pandemic characteristics, which presents a major challenge for any DCT implementation campaign.

### Relaxing Models

#### Overview

Relaxing NPIs allow agents to resume routine behavior, reducing the amount of time in confinement. Relaxing NPIs can reduce the social and economic burden of NPIs, for example, on public services such as gastronomy.

#### RNT

Under the RNT intervention, confined agents are required to obtain a negative test result before returning to normal activities before the default 14-day delay. We simulate a waiting period between test taking by sampling a distribution Y∼2+Γ(6,0.45). The wait period determines the duration in days between the last positive test and requesting a new test. After requesting the test, agents need to wait 24 hours for the test results. Without RNT, the standard recommendation for confined agents is to wait 14 days before resuming normal activities.

### Restrictive Models

#### Overview

When an agent tests positive, they enter isolation and may notify agents in their contact network, thus triggering contacts to quarantine. During confinement, that is, isolation and quarantine, scheduling models that dictate an agent’s location changes are suspended. Agents resume their regular routines based on specific quarantine and isolation exit protocols.

#### QCH

During the COVID-19 pandemic, lockdowns and the emphasis on isolating and quarantining at home led to households becoming primary infection locations [65,66]. Our quarantine contact person household (QCH) intervention explores the potential impact of quarantining entire households when a member is isolated or quarantined. Thus, QCH aims to contain the virus spread within a single household, preventing further transmission to other households. QCH is implemented by using the Zeitgeber to stop allocating location changes for all agents in the household.

#### CBR

Closure of social facilities, for example, bars and restaurants, was prevalent during the pandemic [67-69]. To implement closure of bars and restaurants (CBR), we disabled the scheduling model that facilitates agents’ night-out activities. We focused on a static scenario, that is, the status of gastronomy services remains constant throughout the simulation run, thus reflecting the continuous closure of these establishments during certain periods of the pandemic.

#### Quarantine Rules

Agents go into quarantine after receiving a contact notification through any contact tracing strategy and QCH, if applicable. The agent exits quarantine if one of the following conditions is met: (1) after the regular 14 days quarantine period has passed, (2) after receiving a negative test if RNT is used, (3) after all household members test negative, if QCH and RNT are applied together, and (4) by becoming infected, in which case, the agent is considered isolated and no longer quarantined. Recovered agents do not quarantine or comply with QCH after receiving a notification.

#### Isolation Procedure

Symptomatic agents wait 1.5 days after symptom onset to get tested. Tests can be performed only during the test center opening hours between 8 AM and 4:30 PM. Agents receive test results after 24 hours. After receiving a positive test result, the agent may go into isolation.

### Calibration and Validation

#### Overview

Our calibration and validation efforts focused on selecting parameter configurations that let the ABM model respond in agreement with existing literature. We validated that the ABM model was able to reproduce activity patterns, contact patterns, and epidemiological observations specific to COVID-19. We performed sensitivity analyses for adherence, adoption, and compliance behavior parameters as they are the focus of this work. For simplicity, we marked the model outcomes as individual-to-mass behaviour (I2MB). Please see the Supplementary material in Multimedia Appendix 2 for a detailed explanation of the calibration and validation process.

Throughout this work, we use 95% CI using 100,000 bootstrap resamples to evaluate statistical significance since some of the quantities did not fulfill the normality assumption required to estimate the *P* value.

#### Daily Activity Patterns

We compared daily instances per agent and daily cumulative activity duration per agent with the Extrasensory [70] dataset. The Extrasensory dataset was selected because it matched the activity granularity from our model and contained dense labeling for 1 week of study duration. We simulated 50 independent runs of 1000 agents; each run covered 7 days, without a pathogen model. We created an associative mapping of Extrasensory’s activities to I2MB activities. For example, lying down (not sleeping), watching television, and sitting at home labels in Extrasensory were mapped to *OtherHomeActivities* activity in I2MB. Please refer to the supplementary material in Multimedia Appendix 2 to obtain the complete mapping.

Our validation results show that *CommuteCar, Eat, Grooming, OtherHomeActivities* can be considered equal in the daily instances normalized per performing individuals, as the difference of means was not statistically significant. All other activities presented statistically significant differences of means between Extrasensory and I2BM, but we considered them not practically relevant as such differences did not exceed 2 instances per day. For daily activity instances normalized by total population, we observed that all activities but *EatOut* (CI −0.03 to 0.0) presented statistically significant differences of means. OtherHomeActivities (CI 3.51-3.96) and CoffeeBreak (CI 1.98-2.11) were the only activities where the difference of means exceeded the 2 instances per day. Nevertheless, we consider the differences, within a reasonable variation of expected habits, between the Extrasensory study population that primarily covered student campus life and our simulation that reflects a working population.

Daily activity durations show practical levels of agreement too (refer to Table S2 in the supplementary material in Multimedia Appendix 2). Distribution differences can be explained by the Extrasensory study population and its geographical location, that is, Extrasensory included university students from 1 city (San Diego, California). In particular, total daily commuting durations were shorter in Extrasensory, with a median of 20 min, whereas our simulated commutes had a median duration of 70 min in public transport (CI 46-54). In addition, OtherHomeActivity was consistently longer (CI 205-275), which can be attributed to the limited leisure activities provided in the simulation scenario. Detailed information about the validation of activity instances and duration is provided in the supplementary material in Multimedia Appendix 2.

#### Contact Patterns

We validated the contact patterns generated by the I2MB simulator against Tomori et al [71], who investigated contact person frequencies in different pandemic studies, referred to as waves. We replicated behavior patterns of POLYMOD and COVIMOD study conditions [71] by simulating (1) no intervention, (2) CBR, and (3) a population fraction staying at home.

Here we focus on the behavior of contact persons per location and overall. For simplicity, age and gender were not included in the I2MB simulator model. Unlike Tomori et al [71], we did not apply any normalization weights per household size as the simulations followed Germany’s household size distribution [72]. NPI restrictions during each COVIMOD wave were extracted from the first, fourth, fifth, and sixth Bavarian Infection Protection Measures Ordinance [73]. Please see the supplementary material in Multimedia Appendix 2 on how the waves were simulated.

Overall, our simulations matched contact person distributions for all study conditions. While there were statistically significant differences between the means, these differences were less than 2 contacts with the exception of others in POLYMOD (eg, 3.3 weighted contacts CI 3.23-3.40) and POLYMOD Work (eg, 15 weighted and with group contacts CI 14.41-15.59) where I2MB reported significantly lower contacts than the baseline. The latter difference can be explained by the group contacts assessment, where store workers would report their number of contacts in bulk. For simplicity, the special case of group contact assessment was not covered in our simulation.

The largest differences were observed for COVIMOD waves 1 and 2, which can be attributed to differences in average household sizes. For our simulations, the average household size was 2 residents. In contrast, the sampled population in POLYMOD and COVIMOD studies had an average household size of 3. Consequently, simulations showed 1 contact person on average, while COVIMOD reported 2. As restrictions were relaxed in subsequent waves, contact persons increased and nonhousehold contact persons became more relevant. We consider that the virtual world infrastructure always remains a limited abstraction of the real world, even if further spaces would be added. Thus, behavior in a modeled virtual world is already restricted. If NPIs do not show benefits in the modeled virtual world, they will likely fail in the real world too.

#### Pathogen Characteristics

To evaluate the pathogen propagation model, we simulated the individual behavior of 1000 agents for a full pandemic duration. MCT and ICT were included in the simulations to align with the available real-world data reported in the literature.

Our pathogen propagation model has 3 functional parameters: infectiousness level, exposure function, and viral load removal function. Most model parameters were based on available evidence, for example, Kissler et al [53]. We empirically determined the viral load exhaled by an infected individual j as $\frac{1}{6}\cdot il_{j}(t)$. Please see the supplementary material in Multimedia Appendix 2 and the ODD protocol for additional information in Multimedia Appendix 1.

While the emerging distributions for serial interval (CI −0.68 to −0.54 d), generation interval (CI −0.11 to −0.01 d), incubation period (CI −0.19 to −0.12 d), and illness duration (CI −0.47 to −0.40 d) had all statistically significant differences of means, the fraction of a day differences were practically irrelevant.

In addition to the epidemiological variables, we compared our simulated location of infection against the 30% at home-37% at work-33% at community rule [74]. We defined the infection location as the place where the exposed agent received the last load to pass the viral load threshold. Our analysis showed that ICT was necessary to match the rule at 29% (CI 26%-31%), 37% (CI 33%-40%), and 34% (CI 29%-41%) for home, office, and community. Without ICT, 64% (CI 64%-65%) of the infections happened at work and 34% (CI 33%-34%) at home, which corresponds to where individuals spent most of their time.

#### Contact Network Shapes

The behavioral approach used in this study yields contact networks with similar shapes to those generated by agent-based simulators [23]. However, the emergent contact networks in our simulation have contact-specific weights. To sensibly configure weights in a top-down approach that is typically applied in ABM simulators would necessitate data that may not be readily accessible. Figure S10 (supplementary material in Multimedia Appendix 2) illustrates the heterogeneity of networks per contact type, along with the corresponding weights indicating cumulative contact duration. Additionally, we calculated the median daily ratio of risk contacts at home vs all risk contacts (supplementary material in Multimedia Appendix 2 for details).

### Evaluation

#### Overview

The behavior-driven ABM was implemented by the I2MB simulator [30] written in Python (Python Software Foundation). Our simulation generates and tracks the physical positions of 1000 agents in an appropriately sized virtual world, containing 500 apartments, 20 offices, 5 busses for public transport, as well as a hospital, a restaurant, and a bar (Supplementary material Figure S1 in Multimedia Appendix 2 and the Supplementary ODD protocol for further details in Multimedia Appendix 1). Simulations were performed with a time step of 5 minutes. Each pandemic simulation ran until there were no infectious agents in the population left (typically after 40-100 simulation days). Simulations were repeated 50 times for each parameter configuration, that is, specific behavior parameter settings, and NPI combinations; each simulation run had new agents and thus new home and office assignments, friends and acquaintances networks, and agent infectiousness profiles. At the beginning of each simulation run, 5 randomly selected agents were infected. We defined the default intervention as all symptomatic agents will be tested, receive a positive test result, and isolate upon the positive test. The agents wait for 1.5 days after symptom onset and request the test between 8 AM and 4:30 PM. The test takes 24 hours to complete. After 14 days, the positively tested agent can leave isolation and resume normal behavior. In addition, we applied MCT and ICT in all simulations.

As a consequence of our multishot viral model, when computing serial and generation intervals, it is not reasonable to assign the source of infection to a single agent. Therefore, we derived serial and generation intervals by considering all contact persons that contributed to an exposed agent crossing the viral load threshold and becoming infected. As a result, an infected agent contributes samples of serial and generation intervals, 1 for each considered contact person at the time of infection.

Our evaluation strategy first explored how behavior parameters (ie, adoption, adherence, and compliance) modulate DCT’s ability to control the pandemic. Subsequently, we explore how NPI combinations affect key pandemic characteristics.

#### Exploration of DCT Effects

To explore DCT effects on a population during a pandemic, we simulated parameter configurations for DCT adoption, adherence, and DCT compliance [12]. A total of 38,400 simulation runs were performed for the behavior parameter exploration (8×8 behavior parameter grid points, 50 simulation runs per grid point, 4 pandemic characteristics, and 3 constant behavior parameter set points). We included MCT and ICT methods for all simulations, as they would coexist in a real scenario (MCT and ICT set to default parameters; see Methods section). Guided by estimations for the COVID-19 pandemic, we selected the following set points for behavior parameters: adoption: 0.5 [75], adherence: 0.7 [76], compliance: 0.7 [77,78]. Furthermore, for each set point and associated grid, we uniformly sampled 1000 paths of length 3. Each step in the path varied only in 1 parameter. We used the sampled paths in a Morris [79] sensitivity analysis.

#### NPI Interaction

We evaluated NPI interactions by looking into 2 scenarios. First, we consider the optimal behavior scenario where adoption, adherence, and DCT and MCT compliance were set to 1. ICT compliance was left at the predefined compliance values (refer to Methods section for details). A realistic scenario was defined by setting adoption: 0.5 [75], adherence: 0.7 [76], and compliance: 0.7 [77,78] to estimated parameters from the COVID-19 pandemic. For the optimal scenario, we simulated pandemic progression for MCT and ICT alone, and for DCT in different combinations with RNT, QCH, and CBR. For the realistic scenario, we evaluated all possible NPI combinations. Thus, for 16 NPI combinations of realistic behavior and 9 NPI combinations of optimal behavior, a total of 1250 individual pandemic simulation runs were performed. Table 2 summarizes the scenarios and the behavior parameter settings.

**Table 2.** Summary of behavior parameter settings for the optimal and realistic simulation scenarios.

Scenario	Adoption	Adherence	Compliance (DCT, MCT)	ICT compliance
Optimal behavior	1	1	1	Family: 0.99; friend: 0.90; acquaintance: 0.5
Realistic behavior	0.5	0.7	DCT: 0.7; MCT: 0.95	Family: 0.99; friend: 0.90; acquaintance: 0.5

### Ethical Consideration

This research does not constitute human-subjects research. All results were generated from a synthetic ABM of randomly generated populations. No human participants were enrolled, no identifiable private information was accessed, and no individual-level records (medical, administrative, or otherwise) were used. Parametrization relied only on population-level aggregated statistics. Accordingly, ethics board approval was not applicable.

## Results

### Overview

To analyze DCT effects, we present a prospective simulation-based analysis that builds on contacts emerging from an individual, behavior-based modeling approach. Our ABM model uses parameters that had to be set empirically or informed by literature. The validation of key components (ie, population activities and behavior, agent contacts, and pathogen propagation properties) is detailed in Calibration and Validation section (in addition, see the Supplementary ODD protocol in Multimedia Appendix 1). We performed a parametric search across the DCT adoption-adherence-compliance space and evaluated the parameters’ combined effect on key pandemic characteristics. Moreover, we show the effect of selected NPIs in combination with DCT in optimal and realistic behavior scenarios.

### Exploration of DCT Effects

Figure 5 visualizes simulation results for pandemic characteristics when sweeping behavior parameters (adoption, adherence, and compliance) specifically for DCT. Table 3 shows the results for the sensitivity analysis based on the parameter grids of Figure 5. We derived that any path toward the global maxima across all behavior parameters will unlikely yield a monotonic improvement of the pandemic characteristics. From Figure 5, it becomes clear that the public perception of DCT effectiveness may be discouraged during its implementation, thus further inhibiting DCT adoption. In more detail, the Morris sensitivity analysis (Table 3) suggests the following relevance ranking (decreasing order on the effect size μ*) across all pandemic characteristics, except for “total infected”: compliance, adoption, and adherence. For “total infected,” the ranking is adoption, compliance, and adherence, which is sensible because if people do not download and activate the app, they cannot comply. The parameter exploration furthermore suggests that at low adoption, the 10% region is not reached for any pandemic characteristic. Therefore, DCT adoption (ie, user base) should be maximized first in a DCT implementation campaign. Based on the fluctuation and corresponding sensitivity effect of “total infections” and “days in quarantine” across the behavior parameters, we conclude that adherence (25.2%, CI 25%-26% and 12.9, CI 12.6-13.2 d at 70% compliance) and DCT compliance (26.9%, CI 26.4%-27.4% and 13.8, CI 13.5-14.1 d at 70% adoption) are similarly important. However, after maximizing adoption, our results indicate that prioritizing adherence over DCT compliance may lead to the lowest 10% region of the pandemic characteristics, even at a DCT adoption far below the maximum. In more detail, the total infections and the 7-day incidence metrics approach a global minimum (low 10% boundary region), with DCT compliance at 70%, adoption above 90%, and adherence at 50%. In contrast, fixing DCT adherence at 70% requires adoption above 70% to access the lowest 10% region of each pandemic characteristic.

**Table 3.** Metrics from Morris sensitivity analysis for selected pandemic characteristics. The effect size variance σ is consistently higher than the effect size μ* suggesting that there is interaction between the parameters. The parameters are ranked by selecting the highest between the pairs in a head-to-head ranking.

Set point, pandemic characteristic, and parameters	μ* (95% CI)	σ
DCT^a^ adoption=50%		
Days in quarantine (d)		
Adherence	12 (11.85-12.15)	21.7
DCT compliance	13.6 (13.45-13.75)	23.4
Total infected (%)		
Adherence	29.3 (29.05-29.55)	43.3
DCT compliance	34.9 (34.6-35.2)	46.5
7-day Incidence (cases/100,000 pop)		
Adherence	676.8 (670.65-682.95)	1004.2
DCT compliance	811.6 (803.9-819.3)	1068.1
7-day hospital incidence (cases/100,000 pop)		
Adherence	160.5 (159-162)	238.4
DCT compliance	193.1 (191.25-194.95)	253.6
Adherence=70%		
Days in quarantine (d)		
Adherence	12.9 (12.75-13.05)	21.2
DCT compliance	13.8 (13.65-13.95)	22.3
Total infected (%)		
Adherence	27.4 (27.15-27.65)	40.9
DCT compliance	26.9 (26.65-27.15)	39
* *7-day incidence (cases/100,000 pop)		
Adherence	638.2 (632.45-643.95)	940.5
DCT compliance	680.6 (674.45-686.75)	924.9
7-day hospital incidence (cases/100,000 pop)		
Adherence	151.7 (150.35-153.05)	223.1
DCT compliance	162.6 (161.15-164.05)	220.1
DCT compliance=70%		
* *Days in quarantine (d)		
Adherence	13.7 (13.55-13.85)	21.8
DCT compliance	12.9 (12.75-13.05)	21.4
Total infected (%)		
Adherence	29.3 (29.1-29.5)	42.2
DCT compliance	25.2 (24.95-25.45)	37.9
* *7-day incidence (cases/100,000 pop)		
Adherence	683.4 (678.15-688.65)	970.2
DCT compliance	630.8 (625.55-636.05)	907.3
* *7-day hospital incidence (cases/100,000 pop)		
Adherence	163.1 (161.85-164.35)	230.9
DCT compliance	150.1 (148.85-151.35)	215.6

a DCT: digital contact tracing.

### NPI Interaction

For all pandemic characteristics, we report the median of all the 50 runs with their corresponding IQR and 95% CI for difference of medians. Cohen *d* compatible effect sizes are reported in the supplementary material in Multimedia Appendix 2. The analysis shows that DCT, under optimal behavior, can improve pandemic characteristics compared with MCT and ICT alone. For example, without DCT, 65% (IQR 58%-69%) of the population are infected, whereas with DCT, infections reduce to 4% (IQR 3%-7%) at less than half of the wave duration (36 d, IQR 31-42 d). Moreover, days in quarantine are less with DCT than without it (−11 d, CI −12d to −10 d). For the optimal behavior scenario, additional NPIs, with the exception of RNT, do not show an additional effect on the pandemic characteristics. RNT in addition to DCT can reduce the days in quarantine by 10 (CI −10 to −9) days when compared with DCT alone. In summary, DCT has a profound effect on pandemic characteristics under optimal behavior, which could be improved only in combination with RNT if the goal was to reduce the days in quarantine.

For the realistic behavior scenario, Figure 6 illustrates pandemic characteristic ranges for different NPIs when applied with or without DCT. RNT has a negative effect on pandemic characteristics as it sends agents back to the vulnerable pool. Our results confirm that RNT, without DCT, could perform worse than MCT alone, for example, increasing “total infected” by 5% (CI 0.7%-10%). When RNT is applied in addition to DCT, “total infected,” “7-day incidences,” and “wave duration” increase and “days in quarantine” is reduced by 17 (CI −20 to −5) days with respect to DCT without RNT.

Using DCT in the optimal behavior scenario lowers the serial (5 d, IQR 3-7 d) and generation (5 d, IQR 3-7 d) intervals to range at or below the median incubation time of approximately 4.5 days. Hence, most interactions that lead to an infection occur at the end of the incubation period. In contrast, in the realistic behavior scenarios, “serial interval” (−1 d, CI −2 to 1 d) and “generation interval” (−1 d, CI −2 to 0.3 d) remain unaffected by DCT. The 30% of noncompliant agents in our realistic behavior scenarios with DCT applied, infected more people after their symptom onset compared with compliant agents. In particular, noncompliant agents increased “generation interval” and “serial interval” to 5 (IQR 3-7) days each, compared with 4.5 (IQR 3-6) days and 4.7 (IQR 3-7) days in the optimal scenario. Due to noncompliant agents, generation and serial intervals are similar to the configuration without DCT. While numeric results are calculated using medians of means per run, we randomly selected 50 agents with “days in quarantine,” and 50 instances of “generation interval” and “serial interval” each, measured from infectious-infected agent pairs, to build the corresponding distributions in Figure 6.

Different NPI combinations may yield similar results when applied together with DCT, as illustrated in Figure 6. By “total infected,” DCT alone (38%, IQR 26%-45%) performs similarly to DCT with QCH and CBR (38.6%, IQR 20%-47%), for example, a not statistically significant difference of 1.6% (CI −21% to 12%). Furthermore, DCT and QCH (30%, IQR 18%-41%) perform similarly to DCT and CBR (32.6%, IQR 11%-43%), for example, no statistically significant difference of 8.7% (CI −31% to 19%). The best pandemic characteristics are found for applying DCT and either QCH or CBR, but not both. Further interpretation is provided in the Discussion section. For a detailed comparison between NPI configuration, refer to the supplementary material in Multimedia Appendix 2.

A side effect of DCT is that the median false discovery rate increases from 88.4% to up to 97% for optimal behavior. When introducing DCT in realistic behavior scenarios without RNT, the false discovery rate is approximately 93%, depending on the combination of QCH and CBR. In contrast, the false discovery rate for realistic behavior scenarios drops to approximately 76% when using RNT in combination with DCT, and to approximately 62% without DCT. Without DCT and RCT, combinations of QCH and CBR have a median false discovery rate of approximately 88% (supplementary material in Multimedia Appendix 2).

In our simulation of realistic behavior scenarios, the false negative rate was between 26% and 32%, and for optimal scenarios, the false negative rate varied between 24% and 36%. False discovery and false negative rates are shown in Table S9 in Multimedia Appendix 2 for the optimal and in Table S10 in Multimedia Appendix 2 for realistic behavior.

We note that when applying RNT, the false negative rate was larger for the optimal than the realistic scenarios including DCT. By analyzing the optimal scenario, we found that false negatives are generated by (1) unnotified asymptomatic agents, and (2) differences between our agent pathogen model and the standard DCT app behavior. Due to the asymptomatic proportion of 40% (n=1000), it is likely that infection chains of exclusively asymptomatic agents occur, which remain undetected by DCT. As RNT lets susceptible agents return to normal activities, they may get in contact with asymptomatic agents and hence get infected. In contrast, in a realistic scenario using DCT, noncompliant agents, while being infectious, continue to trace their contacts. Thus, upon the contact between a noncompliant and an asymptomatic agent, DCT notifies the latter of an at-risk contact, as well as recommends testing and subsequent isolation of the asymptomatic agent.

Another reason for false negatives, even in the optimal behavior scenario, is a discrepancy between our multishot [44,51] pathogen model and the simulated DCT app behavior. According to our pathogen model, agents who accumulate sufficient viral load through multiple exposures of less than 15 minutes each will eventually get infected. However, our simulated DCT app follows the original World Health Organization (WHO) recommendations [80], where infection is assumed after 15 minutes of continuous exposure (ie, one-shot assumption [44]), thus effectively failing to capture agents that got infected through multiple shorter exposures.

**Figure 6.**
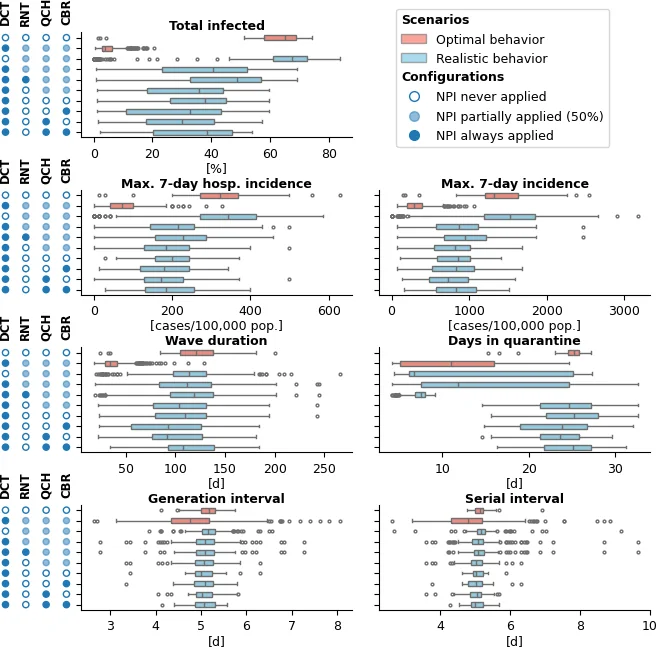
Exploration of NPI combinations regarding their impact on pandemic characteristics. Besides contact tracing, the following NPIs were considered: “Require Negative Test to exit” (RNT), “Quarantine Contact person Household” (QCH), “Closure of Bars and Restaurants” (CBR). Two scenarios were considered: (1) optimal behavior (maximum DCT adoption, DCT adherence, and DCT/MCT compliance) and (2) realistic behavior (DCT adoption: 50%, DCT adherence: 70%, DCT/MCT compliance: 70%; see Table 2). Results show that NPI effects do not stack, but may improve different characteristics. For example, configurations with RNT have a higher total infected population, but fewer days in quarantine, and both QCH and CBR have a mutually exclusive effect on pandemic characteristics. Generation intervals and serial intervals are not affected by the explored NPIs. All simulations include MCT and ICT. We used subsampling to show distributions of “Days in quarantine,” “Generation interval,” and “Serial interval.”

## Discussion

### Principal Findings

We investigated the impact of individual behavior on the effectiveness of DCT and the interaction of DCT with multiple other NPIs to guide future pandemic control efforts and prioritization for pandemic preparedness. Our methodology is motivated by the observation that NPIs can be encoded as behavior rules that affect individual choices and actions. Consequently, a microlevel behavior-driven ABM simulation was proposed, where a Zeitgeber architecture orchestrates activity patterns for every agent that lead to varied agent interactions and key behavior that affects pandemic progression, for example, staying home. Along with the detailed, behavior-driven ABM, we deployed a deterministic multishot viral load transfer model for respiratory pathogens. Our behavior-driven ABM was able to generate realistic pandemic characteristics, reproduce complex phenomena, including stochastic extinctions (Figure 6), and highlight the complex interaction of NPI combinations. Furthermore, results show that individual behavior in general, and related to DCT (adoption, adherence, and compliance) in particular, affects DCT’s capacity to control a pandemic. Our findings suggest that, when composing NPIs, more is not always better; the performance of NPIs (eg, to reduce or delay infections) varies depending on how the interventions are composed (Figure 6).

### Effectiveness of DCT and Its Combinations With Other NPIs

Our analysis demonstrates that DCT alone profoundly reduces pandemic impact if DCT-related behavior is optimal, with a 92% reduction of the total infections. In realistic behavior scenarios, DCT still reduces total infections by one-third compared with only MCT and ICT. Furthermore, our results show a substantial synergy between specific NPI combinations. In particular, combining DCT with CBR or QCH in realistic behavior scenarios yields the best overall performance; that is, reducing total infections by 20% (CI −36% to −4%), from 38% (IQR 26%-45%) for DCT only to 30% (IQR 18%-41%) for DCT and QCH. Retrospective analyses [7] on the importance of combining DCT with other NPIs corroborate our findings. However, even if individual NPIs have a positive effect on pandemic characteristics, their combination may not, as observed in our results when applying both QCH and CBR with DCT, which is statistically similar to only using DCT with a reduction of only 2% (CI −20% to 11%). As individuals spend more time in close proximity at 1 location, they may form a locally dense contact network and thus infect others. Here, QCH and CBR may have forced more agents to spend time at the home location with an infected household member. However, the reduction in the median daily ratio of risk contacts at home to all risk contacts for QCH vs QCH and CBR is less than 0.1% (supplementary material in Multimedia Appendix 2), suggesting complex interaction mechanisms between NPIs. Further investigation with a behavior-driven modeling approach is needed to understand NPI combination effects.

We found that DCT increases the false discovery rate; that is, DCT leads to a higher proportion of individuals being quarantined. Conversely, DCT decreases the false negative rate; thus, fewer infected individuals are left undetected and unisolated. Moreover, our results show that DCT confines more people per risk contact than any other NPI considered in this analysis. Due to the comparably large false discovery rate of DCT, more of the asymptomatic individuals get notified; thus, tested, and subsequently isolated than without DCT.

Although DCT adoption plays an essential role in pandemic control [4], our analysis confirms that maximum DCT adoption is unnecessary to reduce key pandemic characteristics [81,82]. For example, an adoption of 50% in the realistic behavior scenario yielded a reduction of the total infections by 42%. While DCT introduction campaigns should aim at improving adoption and adherence, pandemic characteristics do not react homogeneously (Figure 5), which may negatively influence public perception. Therefore, it is important to maintain ongoing public motivation strategies to offset any short-term setbacks. Additionally, it is not appropriate to judge the effectiveness of DCT solely based on short-term pandemic indicators, as they might give a misleading impression of DCT’s ineffectiveness in pandemic management.

In a typical ABM, the predefined social networks are characterized by their node degree; that is, the contact person count per agent. Predefined networks are often static; that is, not evolving over time. However, behavior changes induced by NPIs change networks. The relationship between behavior changes and network dynamics is often neither observed nor derived. Consequently, often unverified assumptions need to be made about how behavior changes affect the networks. With a behavior-driven ABM, we replace assumptions about how behavior changes affect network dynamics with assumptions about pandemic-independent, verifiable behavior patterns that can be altered by NPIs. Accordingly, agent interactions create the network dynamics. Emergent networks allow us to evaluate metrics, including degree (ie, contact person count) and strength (ie, aggregated encounter duration across all contacts). Further upscaling of the approach should focus on adequate interaction environments, rather than just increasing population. For example, upscaling the population in the current work would likely maintain the statistical relationship of home and office networks as reported, and thus maintain the match with real data (supplementary material in Multimedia Appendix 2). Network diversity could evolve from adding additional public spaces, for example, parks and shopping malls, where super-spreader events may occur.

Our analysis of network metrics and pandemic characteristics revealed epidemiological implications. In particular, our network dynamics analysis (Supplementary material in Multimedia Appendix 2) showed that infection time and 7-day incidence correlated with network strength rather than network degree. In addition, network strength reflects the change in contact preferences, if an NPI modifies individual behavior. Consequently, the reported risk increase for household members due to isolation [65] is not connected to the average degree of the family network. While the average degree of the family network did not change in our analysis, the average family network strength rose during isolations, which may explain the increased infection risk of household members during isolation periods.

DCT advances over MCT by removing recall issues associated with location, familiarity of contact persons, and time since last contact [61]. Furthermore, a DCT app could track short encounters and is not limited by workforce availability to collect and process contact information. While our settings for MCT focused on natural process delays and did not elicit capacity limitations for the considered population size, the addition of DCT clearly improved pandemic characteristics (Figure 6). Based on our results, we conclude that DCT outperforms MCT risk assessment not only due to unrestricted capacity, but the reduction of contact processing time.

During the COVID-19 pandemic, the US Centers for Disease Control and Prevention (CDC) changed the initial contact tracing rule from 15 minutes of continuous exposure to a cumulative exposure duration of at least 15 minutes within 24 hours [83], which corresponds to our multishot approach in the pathogen model. Our analysis of false negative cases confirms the CDC decision. More infectious pathogens may spread during shorter interactions. For example, at the emergence of the omicron variant, the US CDC further shortened the duration threshold to 10 minutes, due to the variant’s increased infectiousness [84]. At our current simulation time step of 5 minutes, the omicron variant spread could still be represented, but short exposure risk would be underestimated. The simulation time step could be reduced to represent short activities and their exposure risk. Besides the pathogen, DCT technology may affect the simulation time step. For instance, the Apple and Google Exposure Notification Framework [85] implemented a dynamic delay between scans in the range of 2-5 minutes. For investigations on the sampling mechanism, a simulation time step of less than 1 minute is justifiable. However, the simulation time step relates indirectly to the simulation time and processing costs. The simulation time step should be chosen carefully to balance simulation resolution with information gain.

The evaluation of guidelines for isolation and using rapid antigen tests identified that unnecessary quarantines produce an avoidable economic burden [86]. Therefore, days in quarantine can be considered a proxy for the economic burden of individuals for whom working from home is not an option. Our findings suggest that RNT is the only NPI that directly reduces the economic burden of quarantine. However, the quarantine reduction by RNT comes at the cost of increases in other pandemic indicators, including 7-day incidences, total infections, and wave duration (Figure 6).

Mobility-restricting NPIs are disregarded in public opinion. Thus, QCH might be more challenging to implement than CBR. However, our findings indicate that CBR and QCH offer limited differential impact on pandemic characteristics. Therefore, the economic benefit of keeping facilities operational, for example, restaurants, might outweigh the costs associated with enforcing QCH.

### Modeling Approach and Metrics Choice

In our modeling approach, behavior and pathogen transmission are modeled independently. The separation allows modelers to distill complex behaviors into physical interaction parameters, that is, distance and duration, which are then used to control viral load transmission. However, modeling viral load transfer and resulting infection needs to align with behavior modeling. As activities and interactions become more fine-grained, the quantization used to describe virus load transmission must follow. The versatility provided by our Zeitgeber architecture and microlevel, behavior-driven ABM approach pairs with our proposed multishot pathogen model. As a result, we can model the composition of different NPIs and implement regulatory changes like the definition of “close contact” by the US CDC (refer to Effectiveness of DCT and Its Combinations With Other NPIs section).

It is recognized that human behavior plays a critical role in pandemic development, from individual decisions to the level of policies, for example, NPIs. Work by Wang et al [24] and Blanco et al [87] presented simulations of disease spread, including NPIs. While we share their objective to achieve realistic simulations of everyday behavior within detailed small-scale cityscapes, including, for instance, offices and restaurants with typical environmental features and behaviors, our work advances in several key areas. Our approach follows important epidemiological principles by incorporating a pathogen model with multishot viral load transfers. Moreover, we analyze key epidemiological metrics (Table 1). Blanco et al [87] asserted the potential of crowd simulation, in particular at the “atomic” level of individual agents, where infection occurs. The analysis in this study is explicit about emerging macro- and meso-level quantities (eg, total infections and confinements stratified by notification type, see video in Supplementary material in Multimedia Appendix 2). In summary, our work breaks new ground in behavior-driven modeling and simulation by integrating rigorous epidemiological principles and ABM methodologies, thereby offering a robust and scientifically grounded approach for analyzing disease spread and assessing public health interventions.

While the selected pathogen characteristics are well-established, for example, the serial interval, there is no consensus on how to evaluate the combined effect of multiple NPIs. Here, we selected pandemic metrics that are generally recognized and used during the COVID-19 pandemic. Consequently, we did not emphasize metrics that apply to DCT only, for example, turn-around times [12]. Several pandemic metrics (7-d incidence metrics, days in quarantine, and total infections) were derived due to their relevance for different stakeholders, including individuals, the health system, and the public.

Our approach could be extended to design and test NPIs that have not been applied before. In particular, NPIs that depend on a person’s characteristics, for example, household size and viral load profile, may be analyzed with our approach and, more specifically, by reconfiguring the platform provided with this work. The novel NPIs and their combinations would be comparably more challenging to describe in compartmental models, which assume that agents are interchangeable.

### Prospects for Behavior-Driven NPI Policy Design

Our ABM modeling approach improves over existing models in the following ways. First, we break the coupling between behavior, NPI rules, and pathogen transmission, thus providing modelers with fine-grained control over their simulated population. In contrast, many existing models do not have a direct correspondence between parameters and individual behavior changes. For example, the compartment transmission rate in a susceptible, exposed, infected, and recovered (SEIR) model condenses many behavioral and pathological aspects into a single parameter that is hard to associate with behavior changes. Second, our ABM model does not rely on the observations of the pandemic evolution. Therefore, scenarios outside pandemic or epidemic events could be studied, catering to pandemic preparedness. Third, our approach supports context information associated with individuals and pandemic control, which may allow policymakers to consider the effects of geographical, structural, and socioeconomic dynamics.

It is clear that modeling a virtual world will only approximate actual behavior and facilities (Figure 1). For a behavior-driven ABM, virtual world complexity, daily routines, location detail, activity diversity corresponding to routines and locations, as well as agent count, are main factors that affect simulation fidelity and generalizability. Nevertheless, a behavior-driven ABM simulation with the multishot viral load transmission mechanism could serve as a first exploration step when designing and orchestrating NPIs. As if an NPI is ineffective in the constrained behavior-driven ABM simulation, it is unlikely that the NPI will create benefits in reality. For example, while we investigated perfect behavior scenarios, the NPI combinations showed limitations, with results indicating that about 3.5% (1000) of the population was still getting infected. The simulation should therefore be considered as an expected performance boundary, and used to select promising NPI combinations.

For SARS-CoV-2, the original pathogen transmission characteristics were understood within 6 months after the initial outbreak [54] and subsequent variants were characterized within weeks [88,89]. NPI effects on pandemic characteristics require additional time in extent of weeks to months to be determined in an ongoing pandemic [90]. However, quickly understanding the effects of NPIs is essential for policymakers to control pandemic development [91]. Moreover, the population may perceive an NPI and potential NPI adjustments as unnecessary grievances as they restrict personal freedom [67]. Furthermore, evaluating a single NPI’s effect on pandemic characteristics is infeasible as effects cannot be isolated in reality. Behavioral simulation is therefore essential to design and test NPIs regarding their short-term and long-term effects and before implementing them in reality. Our approach equips policymakers with a tool to understand an NPI’s effectiveness for pandemic control, for example, in 7-day incidences and total infections, compared with collateral damage expressed in wave duration, days in quarantine, days in isolation, false discovery rate, and false negative rate. For example, policymakers can use the simulation to analyze the benefits of implementing RNT as a measure to reduce economic burden.

Since simulated agents are not required to match actual individuals, no privacy-critical data from the citizens are required to evaluate the NPI’s effectiveness. With behavior-driven ABMs, no data from previous NPI implementations are necessary to create and simulate contact networks. Consequently, in a virtual world representation and assuming that the underlying pathogen transmission characteristics are known, the NPI analysis time depends on simulation capacity only.

### Limitations

To investigate behavior effects related to DCT and NPIs, we kept ABM model parametrization static for several components, for example, the viral transfer model. Therefore, our analysis does not provide conclusions across wider viral characteristics, location arrangements, etc. However, our current findings provide meaningful insight on NPI composition performance for contact tracing in complex realistic behavior scenarios under a COVID-19 pathogen.

### Curse of Complexity

Our current analysis was focused on Western culture and daily repeated activity patterns in an urban setting, using office spaces, public transport, and bars and restaurants as a surrogate of informal interhousehold interaction. Future studies could explore additional activity patterns influenced by culture, age, and work vs leisure days. Furthermore, other interhousehold activities, including visits to other agents’ homes, could be added to provide additional infection pathways. Moreover, the pathogen model could be adjusted for virus variations. While our approach can be extended to more diverse conditions as described above, our current results illustrate the potential, limitations, and contributions of NPIs, including DCT, alone and in concert. The model complexity [92] of a behavior-driven ABM is controlled by simulation population size *n*. While activities, behaviors, and locations create a linear complexity increase, interaction-based NPIs, for example, contact tracing, have a worst-case quadratic complexity, that is, $O\left(n^{2}\right)$. Our implementation averages a complexity of $O\left(n^{1.3}\right)$ (refer to Runtimes section in supplementary material in Multimedia Appendix 2), resulting in run times of 55 minutes for n*=*1000 and 21 hours for n*=*10,000. The simulation was timed on an Intel Core Ultra 7@4.8 GHz with 68 GB of RAM.

Future work may extend the modeling and simulation and consequently refine understanding of the underlying interaction mechanisms, while still managing complexity. We argue that the flexibility of behavior-driven ABM is 2-sided. While our approach offers countless ways to explore uncharted conditions, it burdens the modeler with decisions on fidelity, thus potentially adding structure without necessarily enhancing understanding and results.

### Conclusions

Behavior-driven ABMs add important insight on DCT effectiveness as well as help to understand the impact of combining and fine-tuning NPI combinations that maximize effectiveness, minimize collateral damage, and avoid more disruptive interventions, including proactive school closures or general lockdowns. Compared with predefined network ABMs, behavior-driven ABMs are easier to adjust and to interrogate regarding specific environments, population routines, and virus properties, and provide additional transparency and explainability that helps to disentangle assumptions. For example, the complexity of behavior patterns inside locations can be modified independently of the pathogen transmission mechanics, which helps identify risky behaviors, rather than assuming a priori that certain behaviors are risky. Future investigations may model additional detail in the pathogen model parametrization, the virtual worlds, and the choice of metrics to measure social and economic impact. Based on the simulation results for a selected region of interest, policy design can balance pandemic impact with cultural differences across communities, thus preventing possibly discriminatory NPIs.

## Supplementary material

10.2196/87527 Multimedia Appendix 1 Overview, design concepts, and details protocol (ODD). Summary of the simulator parameter settings.

10.2196/87527 Multimedia Appendix 2 This supplemental material presents a comprehensive investigation into the validation and calibration of our behavior-driven agent-based simulation engine, termed individual-to-mass behaviour (I2MB). We start by showing how the virtual world is constructed and detailing its infrastructure, followed by validation of daily activity patterns, contact patterns, and the pathogen model. Subsequently, we investigate agent interaction behavior and its effects on selected emerging pandemic characteristics using network dynamic analysis. Finally, we present tables of false discovery rate and false negative rate as referenced in the main article, as well as medians with IQR measurements and 95% CIs of the difference of medians between NPI combinations for each exploration scenario.

10.2196/87527 Multimedia Appendix 3 The video shows a dashboard illustrating agents’ behavior (daytime, daily routines, locations, and activities) and interaction in a virtual world. The video presents an entire simulation run with 100 agents and the results of three simulations with different NPI configurations using DCT-optimal behavior. The configurations used are (1) DCT, (2) DCT+QCH+CBR, and (3) DCT+RNT (for abbreviations, see Box 1 in the main manuscript).

## Abbreviations

ABM: agent-based model
CBR: closure of bars and restaurants
CDC: Centers for Disease Control and Prevention
DCT: digital contact tracing
I2MB: individual-to-mass behaviour
ICT: informal contact person tracing
MCT: manual contact person tracing
NPI: nonpharmaceutical intervention
ODD: overview, design concepts, and details
QCH: quarantine contact person household
RNT: require negative test to exit
SEIR: susceptible, exposed, infected, and recovered
WHO: World Health Organization
