# Supplementary material for: Impact of Digital Contact Tracing and Other Nonpharmaceutical Interventions on Pandemic Control: Microlevel, Behavior-Driven Agent-Based Model Analysis
Source: J Med Internet Res. 2026 Sep 30;28:e87527. doi: 10.2196/87527 (PMC13626189; doi:10.2196/87527)
Supplement: Supplementary file 2 [file jmir-v28-e87527-s002.docx]

**Supplementary Material**

Luis Ignacio Lopera González ^1^, Göran Köber ^2^, Göran Kirchner ^3^, Justus Benzler ^3^, Oliver Amft ^2,4^

^1^ Friedrich Alexander Universität Erlangen Nürnberg

^2^ University of Freiburg

^3^ Robert Koch Institut

^4^ Hahn Schickard

**Table of contents**

**Introduction	2**

**Virtual world infrastructure	3**

**Validation and calibration	4**

**Daily activity patterns	4**

**Contact patterns	6**

**Overall contact patterns	8**

**Location-based contact patterns	8**

**Conclusion on contact pattern validation	10**

**Pathogen characteristics	11**

Runtimes	13

**Network dynamics	13**

**Contact network shapes	14**

**Correlation between network metrics and pandemic characteristics	17**

**Analysis of daily contacts, degree, and strength	22**

Risk Contact Ratios	26

**False discovery rate and false negative rate	27**

**Medians and Inter Quartile Ranges Realistic Behaviour Optimal behaviour	29**

**Medians and Inter Quartile Ranges Realistic Behaviour	30**

**Pandemic characteristics difference of medians with 95% Confidence Intervals for Optimal Behaviour	31**

Total Infected	31

Max. 7-day Hosp. Incidence	32

Max. 7-day Incidence	33

Wave Duration	34

Generation Interval	36

Serial Interval	37

**Pandemic characteristics difference of medians with 95% Confidence Intervals for Realistic Behaviour	38**

Total Infected	38

Max. 7-day Incidence	40

Max. 7-day Hosp. Incidence	42

Wave Duration	44

Days in Quarantine	46

Generation Interval	48

Serial Interval	50

**Standard Relative Error	52**

Optimal Behaviour	52

Realistic Behaviour	53

**Effect sizes	54**

Total Infected	55

Optimal Behaviour	55

Realistic Behaviour	56

Max. 7-day Hosp. Incidence	57

Optimal Behaviour	57

Max. 7-day Incidence	59

Optimal Behaviour	59

Realistic Behaviour	60

Wave Duration	61

Optimal Behaviour	61

Realistic Behaviour	62

Days in Quarantine	63

Optimal Behaviour	63

Realistic Behaviour	64

Generation Interval	65

Optimal Behaviour	65

Realistic Behaviour	66

Serial Interval	67

Optimal Behaviour	67

Realistic Behaviour	68

**References	69**

# Introduction

This supplemental material presents a comprehensive investigation on the validation and calibration of our behaviour-driven agent-based simulation engine, termed individual-to-mass behaviour (I2MB). We start by showing how the virtual world is constructed and detailed the virtual world’s infrastructure, followed by the validation of daily activity patterns, contact patterns, and the pathogen model. Subsequently, we investigate agent interaction behaviour and its effects on selected emerging pandemic characteristics using network dynamic analysis. Finally, we present tables of false discovery rate and false negative rate as referenced in the main article.

The I2MB simulator was implemented in Python and follows a modular design. The main article presents the abstractions and the theory behind the I2MB implementation. The technical implementation details for the I2MB engine and each module can be found in the accompanying Overview, Design Concepts & Details (ODD) protocol and [GitHub repository](https://github.com/i2mb/i2mb-core).

# Virtual world infrastructure

Given the characteristics of the COVID-19 virus and the comparably low risk of outdoor contagion, our simulations focused on indoor locations. For simplicity, the hospital and cemetery do not have internal partitions since the interaction between agents at these locations does not contribute to virus transmission and they can be disregarded in contact statistics.

*Figure S1:* Illustration of the virtual world infrastructure scaled for 100 agents. Life outside of the modelled spaces, e.g. homes, cars, etc. was not considered.

The virtual world depicted in Figure S1 was scaled proportional to the population (see ODD protocol for initialisations). Agents were assigned homes such that the number of agents per home distribution matched the 2020 German statistics[1]. Each home was assigned a private means for commuting, represented by a car in the virtual world. The car serves as a proxy for commuting by foot or other private means of transportation. A proportion of the population is selected at the beginning of the simulation to commute by bus. Please refer to the ODD protocol for details.

*Figure S2:* Some of the buildings were given internal structures to add spatial reference for activity execution, including (a) rooms in the home, and tables in the (c) office / classroom, (d) bar, and (e) restaurant, while (b) bus seats were used to spatially arrange agents during rides. Dimensions of all virtual world structures were derived from actual building sizes, and reference material, e.g., public bus manufacturer catalogue.

Each location provides a unique set of activities that can be performed by agents. For simplicity, we restricted activities to locations or to specific furniture, as depicted in Figure S2. For example, homes were fitted with several subspaces and furniture, allowing agents to engage in different activities, including Resting by sitting on the couch in the living room. Similarly, the activity EatingOut happens when the agent is at the bar or restaurant.

# Validation and calibration

## Daily activity patterns

To validate activity patterns, we matched daily instances per agent and daily cumulative activity duration per agent with existing datasets. We selected the Extrasensory[2] (ES) dataset as baseline because its labelled activity granularity matched ours. In ES, 60 participants annotated their activities e.g., sleeping, resting, working, for a week. While larger activity recognition repositories exist, e.g., UK biobank[3] with 100,000 participants, and an average 7.6 days of recording, labels are sparse and the granularity limited to individual actions such as typing or drinking from a cup, which are not comparable to our activity abstractions. We simulated 50 independent runs of 1,000 agents, each run covered seven days, without a pathogen model. We created an associative mapping of ES’ activities to I2MB activities. For example, lying down (not sleeping), watching tv, and sitting at home labels in ES were mapped to OtherHomeAct activity in I2MB. The complete mapping is available in the source code in simulator/activity_utils/extrasensory.py.

In Table S1, we present the average daily activity instance count per agent measured in two ways: (1) normalised by including individuals/agents, who actually performed the activity on a given day, and (2) normalised across the total population, which includes those who did not perform the activity. Due to missing activity logging of the participants in ES, frequencies across the total population appear too low (e.g., toilet, less than once per day). Overall, the simulated (I2MB) frequencies across the total population appear reasonable, and a sufficient overlap between ES and I2MB can be observed.

Compared to the normalisation by total population, normalisation by performing individuals/agents showed a better overlap between ES and I2MB. We assume that when ES participants logged an activity, they did so consistently during that day. No variance was observed for CommuteBus, CommuteCar, and EatOut in I2MB, as each commuting agent did so exactly twice per day, and each agent could only use the EatOut activity once daily. The variances observed for the abovementioned activities in the ES study could be associated with changes in commute mode during a day or that locations in cars and buses were logged in more situations than as part of a daily commute. The largest absolute differences were observed for coffee breaks (1.5/d in ES vs. 2.4/d for simulations) and work (3.9/d in ES vs. 3.0/d for simulations).

|  | **Daily Instances per Individual** | | | | | |
| --- | --- | --- | --- | --- | --- | --- |
| **Activity** | **Normalised by performing individuals/ agents per day** | | | **Normalised by total population** | | |
|  | **ES [**𝛍 ± 𝞼**]** | **I2MB [**𝛍 ± 𝞼**]** | **95% CI** $\boldsymbol{\Delta}\mu$ | **ES [**𝛍 ± 𝞼**]** | **I2MB [**𝛍 ± 𝞼**]** | **95% CI** $\boldsymbol{\Delta}\mu$ |
| **CoffeeBreak** | 1.5 ± 0.8 | 2.4 ± 1.0 | [0.50, 1.32] | 0.0 ± 0.3 | 2.1 ± 1.3 | [1.98, 2.11] |
| **CommuteBus** | 1.6 ± 0.8 | 2.0 ± 0.0 | [0.25, 0.57] | 0.1 ± 0.4 | 0.2 ± 0.6 | [0.08, 0.16] |
| **CommuteCar** | 2.1 ± 1.3 | 2.0 ± 0.0 | [-0.35, 0.10] | 0.2 ± 0.7 | 1.5 ± 0.8 | [1.32, 1.43] |
| **Eat** | 2.0 ± 1.2 | 2.0 ± 0.9 | [-0.15, 0.20] | 0.4 ± 1.0 | 1.7 ± 1.1 | [1.17, 1.32] |
| **EatOut** | 1.3 ± 0.5 | 1.0 ± 0.0 | [-0.42, -0.18] | 0.1 ± 0.3 | 0.0 ± 0.2 | [-0.03, 0.00] |
| **Grooming** | 1.6 ± 0.9 | 1.6 ± 0.5 | [-0.18, 0.18] | 0.1 ± 0.5 | 1.3 ± 0.7 | [1.16, 1.25] |
| **KitchenWork** | 1.5 ± 0.9 | 2.2 ± 1.1 | [0.47, 0.93] | 0.1 ± 0.5 | 1.8 ± 1.3 | [1.61, 1.74] |
| **OtherHomeAct.** | 5.7 ± 6.1 | 5.7 ± 1.9 | [-0.82, 0.60] | 1.2 ± 3.6 | 4.9 ± 2.6 | [3.51, 3.96] |
| **Shower** | 1.2 ± 0.5 | 1.6 ± 0.5 | [0.29, 0.57] | 0.1 ± 0.3 | 1.3 ± 0.7 | [1.23, 1.31] |
| **Sleep** | 1.9 ± 2.2 | 1.2 ± 0.4 | [-1.05, -0.50] | 0.3 ± 1.1 | 0.9 ± 0.6 | [0.50, 0.63] |
| **Toilet** | 1.7 ± 1.0 | 2.1 ± 1.0 | [0.25, 0.74] | 0.1 ± 0.6 | 1.7 ± 1.3 | [1.53, 1.66] |
| **Work** | 3.9 ± 2.7 | 3.0 ± 1.1 | [-1.20, -0.55] | 0.9 ± 2.1 | 2.6 ± 1.4 | [1.63, 1.89] |

*Table S1:* *Averaged daily activity instances per individual in ES and agent in I2MB. Frequencies were computed (1) normalised by performing individuals/agents on the day, and (2) normalised by the total population size. Normalisation by total population showed that some activities were implausibly annotated in ES, e.g. “Toilet” use less than once per day is unlikely. Normalisation by performing individuals/agents showed reasonable overlap between simulation (I2MB) and annotations (ES). Some differences can be explained by the ES participants' student lifestyle that contrast with the urban worker lifestyle assumed in the simulation.*

We randomly selected 1,000 samples per activity from the daily cumulative activity duration per agent generated from 50 runs of behaviour simulations (total approx. 350,000 samples). Figure S3 compares daily cumulative activity duration distributions per agent between simulations and ES. The interquartile ranges (IQRs) of simulated activities overlap with their ES counterparts. Distribution differences can be explained by the ES study population and its geographical location, i.e. ES included university students from one city (San Diego, California). In particular, commuting durations were shorter in ES, with a median of 20 min, whereas our simulated commutes had a median duration of 70 min in public transport. Simulated commuting durations were aligned with those of office workers in urban regions sampled from public transportation network route durations[4].

*Figure S3:* *Daily cumulative activity duration per agent distributions of I2MB simulations in comparison to Extrasensory (ES) study data. The 25th, 50th (median), and 75th percentiles (*interquartile ranges, IQRs) *are shown for all activities. Distribution differences can be attributed to the specific ES population characteristics. ES mainly comprises on-campus students, where duration of commuting and other activities do not conform to whole-population averages.*

|  | **Daily Duration Median** | **Daily Duration Median** | **Daily Duration 95% CI** |
| --- | --- | --- | --- |
|  | **ES** | **I2MB** |  |
| **CoffeeBreak** | 20 | 35 | [5.00, 25.00] |
| **CommuteBus** | 20 | 70 | [45.00, 55.00] |
| **CommuteCar** | 45 | 55 | [-5.00, 20.00] |
| **Eat** | 50 | 40 | [-15.00, 5.00] |
| **EatOut** | 35 | 50 | [0.00, 30.00] |
| **Grooming** | 20 | 20 | [-10.00, 5.00] |
| **KitchenWork** | 27,5 | 15 | [-15.00, -5.00] |
| **OtherHomeAct.** | 150 | 395 | [205.00, 275.00] |
| **Shower** | 20 | 30 | [0.00, 15.00] |
| **Sleep** | 410 | 490 | [50.00, 120.00] |
| **Toilet** | 15 | 15 | [-5.00, 5.00] |
| **Work** | 240 | 375 | [115.00, 175.00] |

*Table S2: 95% Confidence intervals for the difference of medians of the daily duration in minutes. ES is taken as the reference.*

## Contact patterns

We validated the I2MB simulator's generated contact patterns against Tomori et al.[5], who investigated contact person frequencies in different pandemic studies, referred to as waves. For a definition of a contact person, please see the main document. Repeated contacts with the same person within a given time period count only once. In addition, Tomori et al. considered group contacts as the number of remembered contact persons, where listing them individually was not possible, e.g., clients at a store.

We replicated behaviour patterns of POLYMOD and COVIMOD study conditions[5] by simulating (1) no intervention, (2) closure of bars and restaurants (CBR), and (3) a population fraction staying at home. Each condition was simulated 50 times with 1,000 agents for seven days. For each run, we generated new friends and family networks as well as new house and office assignments. We tracked each agent's daily contact persons and stratified them by locations “Home”, “Work”, “Transportation”, and “Other”.

Here we focus on the behaviour of contact persons per location and overall. We did not include age or gender in the simulator model. Unlike Tomori et al., we did not apply any survey or normalisation weights per household size to our simulations since the simulations follow Germany's household size distribution[1]. To compare with Tomori et al., we show both weighted and unweighted data from their result.

NPI restrictions during each COVIMOD wave were extracted from the first, fourth, fifth, and sixth Bavarian Infection Protection Measures Ordinance[6]. For each simulation of a COVIMOD wave, we adjusted the lockdown level by setting the percentage of the population that went to work. The population that had to go to work was split into two groups: fixed and dynamic. The fixed group represents a population share that could not stay at home during daytime. The fixed group was selected at the beginning of each run and had a size of 10% of the total population. The dynamic group was selected every day from the remaining population, with the group size set to 5%, 20%, 40%, and 60% of the total population. Thus, the total percentages of people who went to work were 15%, 30%, 50%, and 70% for COVIMOD waves 1 through 4, respectively. For COVIMOD waves 1 and 2, bars and restaurants were closed. Table S2 details parameter combinations for each COVIMOD wave that was replicated by our simulations.

| **Study / wave** | **Study execution date** | **Restrictions** |
| --- | --- | --- |
| POLYMOD | 2005 - 2006 | No restriction |
| COVIMOD 1 | 30/04 to 06/05/2020 | B&R closure, offices 10% fixed, 5% dynamic |
| COVIMOD 2 | 14/05 to 21/05/2020 | B&R closure, offices 10% fixed, 20% dynamic |
| COVIMOD 3 | 28/05 to 04/06/2020 | B&R closure from 22:00, offices 10% fixed, 40% dynamic |
| COVIMOD 4 | 11/06 to 22/06/2020 | B&R closure from 22:00, offices 10% fixed, 60% dynamic |

*Table S3:* *Summary of simulator configurations (I2MB) to replicate the* POLYMOD and COVIMOD studies*. We used* Tomori et al.’s *study execution dates to estimate the closure of bars and restaurants and the proportion of the population allowed to go to work. See text for details on fixed and dynamic population shares.*

We recreated the plots for the weighted and unweighted contacts, with and without group contacts using a negative binomial distribution with parameters derived from the corresponding mean and standard deviation reported in File 3 of Tomori et al.’s supplemental material[5].

Overall, our simulations matched contact person distributions for all study conditions. The largest differences were observed for COVIMOD waves 1 and 2, which can be attributed to differences in average household sizes. For our simulations, the average household size was two residents. In contrast, the sampled population in POLYMOD and COVIMOD studies had an average household size of three. Consequently, in simulations, we observed one contact person on average, while COVIMOD reports two. As restrictions are relaxed in subsequent waves, contact persons increase and non-household contact persons become more relevant.

### Overall contact patterns

The results show that simulated contact person count increased, as it was observed in Tomori et al.’s results after correcting for group contacts. We find that simulated contact person profiles are within less than Tomori et al.’s reported experiment standard deviation. However, the results also indicate that the virtual world considered here may constrain agent interaction: The overall contact person count is below the reported values by five contact persons on average when considering group contacts and by one contact person when considering only reported contacts as shown in Figure S4.

*Figure S4:* *Box plots of overall contact person distributions estimated by I2MB simulations and reported by Tomori et al.[5] Boxes illustrate the IQR ([25% to 75%]), line marking the median, and white dots the mean. Whiskers indicate 1.5 times IQR. Average contact person counts generated by I2MB simulations are comparable to contact patterns found by Tomori et al.*

### Location-based contact patterns

**Location “Home”.** Figure S5 illustrates distributions of contact persons at location home based on I2MB simulations and Tomori et al. We notice that I2MB simulations produced fewer contact persons than reported by Tomori et al., in particular for POLYMOD. Differences can be explained by the lack of friend and family visits in the simulation and the lack of a joint household activity schedule for agents at home, e.g., dinner time. In our simulations, agents determined their home activities individually.

*Figure S5:* *Box plot of contact person distributions estimated by I2MB simulations and reported by Tomori et al.[5] at location “home”. During the COVIMOD waves, when friends and family visits were suspended, we saw the effect of the joint household schedules and household visits.*

**Location “Work”.** Figure S6 illustrates distributions of contact persons at location work, based on I2MB simulations and Tomori et al. We noticed that I2MB simulation averages reflect the unweighted averages (i.e. without group correction) reported by Tomori et al. Simulations covered 20-person offices only, which may explain why simulations showed smaller IQR than those reported.

*Figure S6:* *Box plot of contact person distributions estimated by I2MB simulations and reported by Tomori et al.[5] at location “work”. On average, I2MB simulation results appear similar to unweighted contact person reports. Our simulations covered up to 20-person offices, which may explain the lower IQR compared to those reported by Tomori et al.*

**Location “Transport”.** Figure S7 illustrates distributions of contact persons at location transport, based on I2MB simulations and Tomori et al.The simulations’ transportation configuration generates contact patterns that match reported values.

*Figure S7:* *I2MB simulated transportation configuration generated contact patterns that match reported values by Tomori et al.*

**Location “Other”.** Figure S8 illustrates distributions of contact persons at location other, based on I2MB simulations and Tomori et al. While restaurant and bar provided agent interactions, the “other” possible contacts were underrepresented. Further work may add other locations and public places, including shops, supermarkets, gyms, and parks.

*Figure S8:* *Contact person counts reported at other locations. I2MB’s simulated contacts in “other” locations are less than one on average for all experiments. We observe that for POLYMOD and COVIMOD 3 and 4, some agents achieve contact persons in the same order of magnitude.*

### Conclusion on contact pattern validation

|  |  | **Unweighted** | **Weighted** | **Unweighted and With Group Contacts** | **Weighted and With Group Contacts** |
| --- | --- | --- | --- | --- | --- |
| **Overall** | **POLYMOD** | [-0.63, -0.33] | [-1.23, -0.91] | [-13.73, -12.59] | [-11.40, -10.44] |
|  | **COVIMOD 1** | [-1.11, -1.02] | [-0.95, -0.87] |  |  |
|  | **COVIMOD 2** | [-2.37, -2.13] | [-1.99, -1.80] |  |  |
|  | **COVIMOD 3** | [-1.02, -0.78] | [-0.90, -0.70] | [-3.74, -2.96] | [-4.26, -3.51] |
|  | **COVIMOD 4** | [0.72, 0.98] | [0.86, 1.11] | [-3.73, -2.53] | [-3.49, -2.38] |
| **Home** | **POLYMOD** | [-1.79, -1.69] | [-2.30, -2.20] | [-1.79, -1.70] | [-2.31, -2.22] |
|  | **COVIMOD 1** | [-0.60, -0.53] | [-0.62, -0.55] |  |  |
|  | **COVIMOD 2** | [-0.62, -0.55] | [-0.78, -0.71] |  |  |
|  | **COVIMOD 3** | [-0.54, -0.47] | [-0.61, -0.54] | [-0.51, -0.44] | [-0.61, -0.54] |
|  | **COVIMOD 4** | [-0.47, -0.40] | [-0.56, -0.49] | [-0.51, -0.44] | [-0.61, -0.54] |
| **Work** | **POLYMOD** | [2.75, 3.00] | [2.43, 2.64] | [-13.40, -12.15] | [-15.59, -14.41] |
|  | **COVIMOD 1** | [-0.40, -0.35] | [-0.19, -0.15] |  |  |
|  | **COVIMOD 2** | [-1.86, -1.61] | [-1.34, -1.09] |  |  |
|  | **COVIMOD 3** | [-0.87, -0.60] | [-0.69, -0.49] | [-3.28, -2.67] | [-4.83, -4.01] |
|  | **COVIMOD 4** | [0.87, 1.14] | [1.14, 1.35] | [-2.73, -1.79] | [-3.44, -2.27] |
| **Transport** | **POLYMOD** | [0.14, 0.20] | [-0.14, -0.06] | [0.14, 0.21] | [-0.19, -0.11] |
|  | **COVIMOD 1** | [-0.03, -0.01] | [-0.01, 0.00] |  |  |
|  | **COVIMOD 2** | [-0.07, -0.05] | [-0.07, -0.05] |  |  |
|  | **COVIMOD 3** | [0.01, 0.04] | [0.10, 0.12] | [0.00, 0.03] | [0.10, 0.12] |
|  | **COVIMOD 4** | [0.11, 0.15] | [0.11, 0.15] | [0.11, 0.16] | [0.10, 0.15] |
| **Others** | **POLYMOD** | [-2.98, -2.84] | [-3.40, -3.23] | [-3.07, -2.91] | [-3.45, -3.27] |
|  | **COVIMOD 1** | [-0.41, -0.37] | [-0.42, -0.37] |  |  |
|  | **COVIMOD 2** | [-1.15, -1.07] | [-0.92, -0.85] |  |  |
|  | **COVIMOD 3** | [-1.10, -0.98] | [-1.01, -0.92] | [-1.96, -1.71] | [-1.57, -1.38] |
|  | **COVIMOD 4** | [-1.11, -1.01] | [-0.95, -0.86] | [-3.57, -2.70] | [-2.49, -1.93] |

*Table S4: 95% Confidence interval of difference of means between Tomori et al simulated distributions and I2MB generated contacts. The confidence interval was computed using 100000 bootstrapping iterations.*

The overall contact patterns appeared sufficiently close between I2MB simulations and reports by Tomori et al. Furthermore, location analysis revealed that household, transport, and work contact patterns were comparable, with differences explained by the lack of inter-household visits and the homogeneity of the work location in the simulations. The “Other” location contact patterns differed more widely due to the virtual space limitations. We consider that the virtual world infrastructure always remains a limited abstraction of the real world, even if further spaces would be added. Thus, behaviour in a modelled virtual world is already restricted. If NPIs do not show benefits in the modelled virtual world, they will likely fail in the real world too.

## Pathogen characteristics

We introduce a mechanistic pathogen propagation model to describe the exchange of viral load (for detail see methods section in the main paper). To evaluate the pathogen propagation model, we simulated the individual behaviour of 1,000 agents for a full pandemic duration. We show probability density functions (PDFs) of epidemiological variables and compare them with observed COVID-19 statistics. MCT and ICT were included in the simulations to align with the available real world data.

The mechanistic pathogen propagation model has three functional parameters: infectiousness level, exposure function, and viral load removal function. The method section in the main text describes these parameters in detail. Although the model’s parameters were based on available evidence, e.g., Kissler et al.[7] We determined the viral load exhaled by an infected individual j as $\frac{1}{6}\cdot il_{j}(t)$. The exhaled viral load term is included in Eq. 3 of the main manuscript.

Figure S9 illustrates selected epidemiological variable distributions. Our simulations widely agree with measured distributions for serial[8] and generation[9] intervals. The overall agreement confirms that contact patterns generated by the simulation match natural contact patterns. Furthermore, the simulations agree with reported measurements of incubation[10] and clearance[7] periods, supporting the validity of our novel pathogen propagation model.

Table S5 shows the 95% confidence interval of the difference of means between the literature and the simulated epidemiological variable. Our simulations widely agree with measured distributions for serial and generation intervals. The overall agreement confirms that contact patterns generated by the simulation match natural contact patterns. Furthermore, the simulations agree with reported measurements of incubation and clearance periods, supporting the validity of our novel pathogen propagation model.

|  | **Serial Interval**[8] | **Generation Interval**[9] | **Incubation Period**[10] | **Illness Duration**[7] |
| --- | --- | --- | --- | --- |
| **Baseline** | [-0.51, -0.39] | [0.08, 0.16] | [-0.19, -0.13] | [-0.46, -0.41] |
| **With Ict** | [-0.63, -0.50] | [-0.05, 0.05] | [-0.16, -0.09] | [-0.45, -0.38] |
| **With Ict Mct** | [-0.68, -0.54] | [-0.11, -0.01] | [-0.19, -0.12] | [-0.47, -0.40] |

*Table S5: 95% Confidence intervals of the difference of means between simulated and the respective reference.*

*Figure S9:* *Comparison of I2MB simulation and reported epidemiological variables for COVID-19. We assume that ICT and MCT were applied during the reference collection. For the simulations, we varied the presence of ICT and MCT in three simulation configurations, to analyse their impact on the epidemiological variables. Overall, simulation results match the references.* Incubation[10] and clearance[7] *periods were not affected by the analysed contact tracing strategies as they are properties of the interaction between the virus and the individual host. In addition, ICT and MCT did neither have an effect on* serial[8] and generation[9] *intervals.*

In addition to the epidemiological variables, we compared where infections happened in the simulation against the 30%@home-37%@work-33%@community rule [11]. We defined the infection location as the place where the exposed individual received the last load to pass the viral load threshold. Table S6 shows the mean, standard deviation, and confidence interval for the different percentages of infections. Our baseline scenario corresponds to a scenario where no one is telling anything about their disease status, and as a result, the infection location matches with where people spend the most time making community infection almost irrelevant. However, when ICT is applied, we see that the results closely match the 30%-37%-33% rule. Adding, MCT shifts infections towards home, but this shift is not significant.

|  |  | **Mean ± SD** | **CI 95%** |
| --- | --- | --- | --- |
| **Baseline** | **Home** | 0.336 ± 0.011 | [0.333, 0.339] |
|  | **Office** | 0.644 ± 0.011 | [0.641, 0.647] |
|  | **Community** | 0.020 ± 0.004 | [0.019, 0.022] |
| **With Ict** | **Home** | 0.291 ± 0.092 | [0.263, 0.313] |
|  | **Office** | 0.365 ± 0.125 | [0.329, 0.397] |
|  | **Community** | 0.344 ± 0.215 | [0.289, 0.409] |
| **With Ict Mct** | **Home** | 0.298 ± 0.077 | [0.275, 0.317] |
|  | **Office** | 0.361 ± 0.101 | [0.330, 0.386] |
|  | **Community** | 0.341 ± 0.177 | [0.298, 0.394] |

*Table S6: Measurement of the percentage of infection locations based on the categories from Ferguson et al.[11]*

## Runtimes

Table S7 shows the average runtime for a full wave and 7 days runs. A full wave represents the time between the start of the simulation until all individuals have gone through the disease, e.g., are immune, or died. The simulator has a runtime complexity of $O\left( n^{1.3} \right)$, where n is the population size. The complexity was calculated by fitting a the log-log transformation of the power law $log(T(n))=log(a)\cdot b\cdot log(n)$ to the log of the data and then extracting the coefficient $b \sim1.3$ for both cases. These runtimes were computed on an Intel core ultra 7@4.8GHz with 68GB of RAM.

|  | **Process time [hh:mm:ss]** | | | |
| --- | --- | --- | --- | --- |
|  | **Full Wave** | | **7-Day Run** | |
| **Population size** | **mean** | **SD** | **mean** | **SD** |
| **100** | 00:03:03 | 00:00:24 | 00:00:20 | 00:00:03 |
| **1000** | 00:55:58 | 00:06:15 | 00:05:08 | 00:00:12 |
| **10000** | 21:12:12 | 00:26:44 | 02:14:44 | 00:02:27 |

*Table S7: Average runtimes for full wave and 7-day runs.*

# Network dynamics

We calculated the networks created by the interactions between agents and compared them with the unweighted networks used in other agent-based simulators. We defined four contact types in our simulation: with family, friends, acquaintances, and random (See main manuscript for details).

For this analysis, we considered four epidemiological concepts with their adapted definitions: (1) contact persons of an agent, regardless whether infectious or not, are the agents within three metres, (2) contacts of an agent are the agent’s encounters with contact persons, (3) contact duration is the accumulated encounter duration between two contact persons, and (4) aggregated contact duration of an agent is the accumulated contact duration with all contact persons of that agent. For example, if agent A met with agent B three times for 5 minutes each time and with no one else, the count of agent A’s contact persons would be one, the count of contacts would be three, and the contact duration would be 15 minutes. We mapped the epidemiological variables #1 and #4 to network metrics as follows: the count of contact persons (of an agent) maps to the degree (of a node), while the aggregated contact duration maps to the strength (of a node) using the contact duration as weight (on an edge). Eq. S1 defines the degree $d_{i}$ as the count of edges $E$ that contain node $i$.

$d_{i}=\left| \{ \forall e \in E : i \in e\} \right|$	(S1)

Eq. S2 illustrates the strength of a node $i$ $s_{i}$ as the sum of the edge property $p$ for all edges containing $i$.

$s_{i}=\sum_{e: e \in E} e_{p}: i \in e$	(S2)

Our analysis is divided into three steps: (1) analyse contact network shapes, (2) correlation between epidemiological variables and network variables, and (3) time series network metrics. Across all, we focus on the network metrics computed using the contact network. We used four scenarios for the analysis: (1) the baseline, where only symptomatic agents isolate, all with compliance of 1.0, (2) MCT and ICT, where people quarantine based on information from friends and families or manual contact tracing following the realistic scenario conditions (see main manuscript for details), (3) realistic behaviour scenario including DCT, where DCT adoption, adherence, and compliance were set to 0.5, 0.7, and 0.7 respectively, and (4) optimal behaviour scenario, where DCT, MCT and ICT are used to inform contact persons of positive cases, and adoption, adherence, and compliance were all set to 1.0.

## Contact network shapes

The behavioural approach employed in this study yields contact networks with similar shapes to those generated by agent-based simulators[12]. However, the resulting contact networks exhibit the emergent property of being independently weighted. In contrast, the top-down approach used in other agent-based simulators necessitates data that may not be readily accessible for configuring the weights of each potential network during setup. Figure S10 illustrates the heterogeneity of networks per contact type, along with the corresponding weights indicated as strengths based on contact duration.

*Figure S10:* *Examples of emerging weighted networks by contact type. These examples come from a simulated week without pathogen presence. For each edge we used the accumulated contact duration property to control the width and shading. The generated behaviour driven encounters create heterogeneous weighted networks, illustrating agent interaction preference. The emergent networks have similar shapes to networks used for implementing traditional ABMs, which supports the realism of the behaviour driven simulation paradigm.*

The differences between min-max normalised network degree and strength distributions for different simulation configurations are illustrated in Figure S11. The degree focuses on contact opportunities, as seen in the bimodal distribution for the random network. For instance, full-time workers are more likely to experience random contacts during their work hours and coffee breaks, public transport commuters during their public transport rides, and diners and patrons when visiting bars and restaurants. In contrast, network strength tracks the cumulative contact duration, which is an unimodal distribution with no discernible groups.

.

*Figure S11:* *Differences between min-max normalised degree and strength distributions, by contact tracing modality and behaviour scenario and by contact type. The degree focuses on contact opportunities, as shown by the multimodality of random networks, where full-time workers and public transport commuters drive the second mode. Strength focuses on the contact duration, follows an unimodal distribution and provides no discernible grouping.*

The lack of connection in the friends' network may be attributed to limited availability of leisure spaces and no inter-household home visits. In future work, we will consider more public spaces, including locations for leisure activities, e.g., parks and malls. In addition, we will enable inter-household visits.

The difference between distributions might lead to different risk assessments and planned interventions. Based on the simulation results, if the degree is used, i.e., unweighted networks, full-time workers, public transport commuters, and gastronomy services, would constitute a target group for contact reduction interventions. In contrast, network strength yields more holistic intervention strategies that would not target any specific social group.

## Correlation between network metrics and pandemic characteristics

We investigate the relationship between network metrics — specifically degree and duration-weighted strength — and pandemic characteristics such as infection time and 7-day incidence. Our analysis focuses on the infection history, comparing the degree and strength of the infector (source) and the infectee (target) in relation to pandemic progression. As shown in **Figure S12**, combining different types of contact tracing pushes contact persons from random to family-based networks. In addition, strength shows a stronger correlation to infection time, especially with digital contact tracing (DCT), highlighting the importance of measuring risk based on contact duration rather than contact person counts.

Further analysis (**Figure S13**) shows that strength correlates more strongly with infection time when measured only before infection, but this insight does not yield actionable prevention strategies as agents infected later in the pandemic have had more time to accumulate strength. When contact metrics are limited to the week preceding infection, the correlation with infection time diminishes (**Figure S14**), suggesting that socially active agents drive the pandemic, even with small social circles. Moreover, as DCT improves, source agents with larger social networks and longer contact durations drive later-stage infections. The relationship between 7-day incidence and network metrics (**Figure S15**) reveals a positive correlation between the source's degree and strength and the 7-day incidence, while targets show a negative correlation, especially under optimal DCT conditions. This indicates that low-incidence periods pose higher infection risks for susceptible agents due to increased interaction duration and the availability of infectious agents. The findings suggest that future DCT implementations could enhance pandemic control by encouraging individuals to monitor and moderate strength, i.e., cumulative contact duration, particularly during periods of low incidence.

*Figure S12:* *Illustration of overall degree and strength, which are measured during the entire simulation, vs. infection time. Overall degree (contact persons [CP]) of the source and target are not correlated to the infection time. Overall strength (aggregated contact duration in days [d]) shows a positive correlation in each of the test scenarios.*

*Figure S13:* *Degree (contact persons [CP]) and strength (aggregated contact duration in days [d]) before the target’s infection time. The improved correlation between strength and infection time yields no actionable insight, as the target has had more time to accumulate connections.*

*Figure S14:* *Degree (contact persons [CP]) and strength (aggregated contact duration in days [d]) measured during the 7-day period prior to the target’s infection. Correlations between strength and infection time collapse when considering the contacts during the 7-day prior to the target’s infection. The negative correlation with the target degree indicates that the pandemic will progress even if the target has small social groups.*

*Figure S15:* *Degree (contact persons [CP]) and strength (aggregated contact duration in days [d]) measured during the 7-day period prior to the target’s infection. The source degree and strength follow the intuition that a socially active source will cause more infections. The negative correlation of the target’s degree and strength indicates that social agents are more at risk when the incidence is low at infection time.*

## Analysis of daily contacts, degree, and strength

In this section, we examine the daily trends of contact metrics—contacts, degree, and strength—across four different scenarios. **Figure S16** highlights the baseline scenario without contact tracing, where contacts, degree, and strength remain largely stable throughout the pandemic. A slight increase in all metrics occurs the day before infection, followed by a small reduction in acquaintances and random contacts as individuals self-isolate. However, no shift toward increased family contact persons is observed during isolation in this scenario. Introducing manual (MCT) and individual contact tracing (ICT), as shown in **Figure S17**, leads to a decline in daily contacts, degree, and strength as the pandemic progresses, returning to pre-pandemic levels by the end of the simulation. Relative time plots reveal that family interactions intensify during quarantine, while contact persons with acquaintances and random individuals decline, aligning with earlier findings that quarantine heightens the risk of household transmission.

In scenarios with digital contact tracing (DCT), MCT, and ICT, **Figure S18** shows a moderate reduction in random contact persons as isolation and quarantine measures take effect, which is not seen in the baseline scenario where all metrics remained constant. The relative time plots show that contact tracing effectively reduces the daily degree after infection, although family degree remains steady, and family strength increases due to home isolation. **Figure S19** demonstrates the impact of quarantine over time, with external contact persons decreasing and family interactions increasing during the first 14 days of the pandemic. As individuals leave isolation after 20 days, metrics begin to normalize, although a secondary reduction in contacts is observed after 90 days. Even under optimal DCT conditions, not all infection chains are broken in the first 20 days. Notably, across all scenarios, there is a slight increase in contacts, degree, and strength on the day before infection, suggesting future work could explore contact proximity as a factor to further refine contact tracing strategies.

*Figure S16:* *Time series data for daily contacts, degree (contact persons), and strength (aggregated contact duration) for the baseline scenario and discarding hospital contacts. Strength is also constant throughout the simulations after removing hospital contacts. A small peak in contacts, degree and strength just before the infection remains.*

*Figure S17:* *Time series data for daily contacts, degree (contact persons), and strength (aggregated contact duration) for the MCT and ICT scenario and discarding hospital contacts. While contacts and degree show a considerable reduction in overall contacts, only strength shows the increased interaction with household members.*

*Figure S18:* *Time series data for daily contacts, degree (contact persons), and strength (aggregated contact duration) for the realistic behaviour scenario and discarding hospital contacts. In the realistic behaviour scenario, we observed a faster degree reduction after infection when compared to MCT and ICT, which highlights the impact of using DCT, as the degree restoration happens at the same rate. Contacts and strength illustrate how family encounters substitute other types of contact during confinement.*

*Figure S19:* *Time series data for daily contacts, degree (contact persons), and strength (aggregated contact duration) for the optimal behaviour scenario and discarding hospital contacts. The simulation time plots show how degree and contacts reduce as positive tests arrive. The time series shows a second wave that occurs, as not all infection chains can be broken.*

## Risk Contact Ratios

The risk contact at home ratio is defined as the median daily ratio of the duration of risk contacts at home to the duration of all risk contacts. A risk contact is the contact of a susceptible or exposed individual with an infected individual.

|  |  |  |  | **Risk contact at home ratio** | | | | | | |
| --- | --- | --- | --- | --- | --- | --- | --- | --- | --- | --- |
|  |  |  |  | **mean** | **std** | **min** | **25%** | **50%** | **75%** | **max** |
| **DCT** | **RNT** | **QCH** | **CBR** |  |  |  |  |  |  |  |
| **Dct** | **No Rnt** | **No Qch** | **No Cbr** | 32,12 % | 5,03 % | 22,82 % | 28,57 % | 31,60 % | 35,20 % | 51,78 % |
|  |  |  | **Cbr** | 31,61 % | 5,24 % | 22,76 % | 27,86 % | 30,74 % | 34,31 % | 55,12 % |
|  |  | **Qch** | **No Cbr** | 31,25 % | 5,11 % | 22,57 % | 27,62 % | 30,50 % | 33,89 % | 100,00 % |
|  |  |  | **Cbr** | 31,40 % | 4,77 % | 22,93 % | 28,04 % | 30,57 % | 33,95 % | 54,03 % |
|  | **Rnt** | **No Qch** | **No Cbr** | 28,78 % | 3,55 % | 11,29 % | 25,92 % | 28,46 % | 31,01 % | 83,37 % |
|  |  |  | **Cbr** | 28,94 % | 3,33 % | 11,23 % | 26,46 % | 28,76 % | 31,10 % | 82,95 % |
|  |  | **Qch** | **No Cbr** | 29,05 % | 3,71 % | 11,91 % | 25,96 % | 28,77 % | 31,43 % | 83,58 % |
|  |  |  | **Cbr** | 28,86 % | 3,40 % | 11,15 % | 26,31 % | 28,65 % | 30,85 % | 84,26 % |
| **No Dct** | **No Rnt** | **No Qch** | **No Cbr** | 31,96 % | 5,44 % | 22,78 % | 27,27 % | 31,64 % | 36,11 % | 49,08 % |
|  |  |  | **Cbr** | 31,75 % | 4,56 % | 13,05 % | 28,10 % | 31,82 % | 35,12 % | 46,25 % |
|  |  | **Qch** | **No Cbr** | 31,59 % | 4,96 % | 22,65 % | 26,93 % | 32,15 % | 35,13 % | 50,88 % |
|  |  |  | **Cbr** | 32,01 % | 5,20 % | 23,04 % | 27,33 % | 32,31 % | 35,74 % | 51,15 % |
|  | **Rnt** | **No Qch** | **No Cbr** | 29,83 % | 4,38 % | 12,08 % | 25,79 % | 29,77 % | 33,21 % | 90,55 % |
|  |  |  | **Cbr** | 30,24 % | 5,05 % | 15,79 % | 25,78 % | 29,76 % | 33,51 % | 88,92 % |
|  |  | **Qch** | **No Cbr** | 30,05 % | 4,89 % | 12,17 % | 25,64 % | 29,52 % | 33,70 % | 86,81 % |
|  |  |  | **Cbr** | 29,87 % | 4,56 % | 12,60 % | 25,76 % | 29,20 % | 33,17 % | 82,99 % |

*Table S8: The difference between the DCT+QCH and DCT+QCH+CBR ratios (amber) is less than 0.1%, suggesting that the NPI interaction mechanisms are more complex and further research is needed.*

# False discovery rate and false negative rate

|  |  |  |  | **False discovery rate** | | **False negative rate** | | **Fowlkes mallows index** | |
| --- | --- | --- | --- | --- | --- | --- | --- | --- | --- |
|  |  |  |  | **Median** | **IQR** | **Median** | **IQR** | **Median** | **IQR** |
| **DCT** | **RNT** | **QCH** | **CBR** |  |  |  |  |  |  |
| **NO DCT** | **NO RNT** | **NO QCH** | **NO CBR** | 88.4% | [88.1%, 88.7%] | 29.1% | [27.8%, 30.1%] | 28.7% | [28.3%, 29.1%] |
| **DCT** | **NO RNT** | **NO QCH** | **NO CBR** | 97.3% | [96.3%, 97.9%] | 24.8% | [21.5%, 28.2%] | 14.2% | [12.4%, 16.3%] |
|  |  |  | **CBR** | 97.5% | [96.8%, 98.3%] | 24.5% | [19.5%, 28.9%] | 13.6% | [11.3%, 14.9%] |
|  |  | **QCH** | **NO CBR** | 97.6% | [97.0%, 98.3%] | 24.2% | [20.5%, 26.1%] | 13.5% | [11.5%, 15.0%] |
|  |  |  | **CBR** | 97.6% | [96.6%, 98.4%] | 24.2% | [19.5%, 27.8%] | 13.9% | [11.3%, 15.8%] |
|  | **RNT** | **NO QCH** | **NO CBR** | 93.4% | [91.3%, 94.9%] | 35.9% | [33.2%, 38.9%] | 20.2% | [18.6%, 23.6%] |
|  |  |  | **CBR** | 92.4% | [90.6%, 94.1%] | 34.4% | [32.2%, 37.9%] | 21.5% | [20.2%, 24.5%] |
|  |  | **QCH** | **NO CBR** | 92.2% | [90.4%, 95.0%] | 35.7% | [32.4%, 38.7%] | 21.8% | [19.1%, 24.8%] |
|  |  |  | **CBR** | 92.8% | [90.7%, 94.5%] | 35.9% | [31.5%, 38.2%] | 22.2% | [19.6%, 24.0%] |

*Table S9:* *False discovery rate and false negative rate for the NPI combinations in the optimal behaviour scenarios. The selected scenarios provide a performance boundary for the pandemic characteristics under study, focusing on using DCT as the primary contributor to performance improvement.*

|  |  |  |  | **False discovery rate** | | **False negative rate** | | **Fowlkes mallows index** | |
| --- | --- | --- | --- | --- | --- | --- | --- | --- | --- |
|  |  |  |  | **Median** | **IQR** | **Median** | **IQR** | **Median** | **IQR** |
| **DCT** | **RNT** | **QCH** | **CBR** |  |  |  |  |  |  |
| **NO DCT** | **NO RNT** | **NO QCH** | **NO CBR** | 88.0% | [87.7%, 88.6%] | 29.5% | [28.3%, 30.7%] | 28.9% | [28.3%, 29.5%] |
|  |  |  | **CBR** | 88.3% | [87.9%, 88.7%] | 29.4% | [28.2%, 30.7%] | 28.7% | [28.3%, 29.3%] |
|  |  | **QCH** | **NO CBR** | 87.9% | [87.5%, 88.5%] | 29.2% | [27.2%, 30.1%] | 29.2% | [28.4%, 29.6%] |
|  |  |  | **CBR** | 88.2% | [87.7%, 88.8%] | 29.6% | [28.1%, 30.7%] | 28.8% | [28.1%, 29.3%] |
|  | **RNT** | **NO QCH** | **NO CBR** | 62.7% | [61.7%, 63.9%] | 32.1% | [31.2%, 32.8%] | 50.5% | [49.6%, 51.1%] |
|  |  |  | **CBR** | 62.9% | [61.6%, 63.8%] | 31.5% | [30.5%, 32.8%] | 50.5% | [49.4%, 51.3%] |
|  |  | **QCH** | **NO CBR** | 63.1% | [61.7%, 64.3%] | 32.1% | [31.1%, 33.3%] | 50.0% | [49.0%, 50.8%] |
|  |  |  | **CBR** | 62.9% | [61.6%, 64.9%] | 32.0% | [30.9%, 33.0%] | 50.3% | [48.6%, 51.0%] |
| **DCT** | **NO RNT** | **NO QCH** | **NO CBR** | 92.6% | [92.2%, 93.7%] | 27.5% | [25.6%, 28.5%] | 23.3% | [21.5%, 23.7%] |
|  |  |  | **CBR** | 93.0% | [92.2%, 94.1%] | 26.5% | [24.3%, 27.6%] | 22.7% | [20.8%, 24.0%] |
|  |  | **QCH** | **NO CBR** | 93.1% | [92.4%, 94.1%] | 27.1% | [25.5%, 28.2%] | 22.5% | [20.8%, 23.4%] |
|  |  |  | **CBR** | 92.8% | [92.1%, 93.4%] | 26.8% | [25.1%, 28.8%] | 23.1% | [21.7%, 24.1%] |
|  | **RNT** | **NO QCH** | **NO CBR** | 76.4% | [75.1%, 78.9%] | 32.0% | [30.7%, 33.2%] | 39.9% | [37.9%, 41.2%] |
|  |  |  | **CBR** | 76.6% | [75.0%, 78.1%] | 32.3% | [30.8%, 34.1%] | 39.9% | [37.8%, 41.1%] |
|  |  | **QCH** | **NO CBR** | 76.5% | [74.6%, 78.2%] | 31.8% | [30.3%, 33.3%] | 40.3% | [38.4%, 41.6%] |
|  |  |  | **CBR** | 76.1% | [74.5%, 78.6%] | 32.2% | [30.1%, 33.3%] | 40.2% | [37.9%, 41.8%] |

*Table S10:* *False discovery rate and false negative rate for the different NPI combinations in the realistic behaviour scenarios.*

*Figure S20:* *False discovery rate and false negative rate distributions for the NPI combinations in the optimal and realistic behaviour scenarios ordered by the mean total infections with lowest total infections at the top. DCT’s success is derived from a low false negative rate (presumably by a slightly faster isolation) at the price of a high false discovery rate, which translates to targeted quarantines that effectively diminishes the risk of infection for highly exposed agents. Note that the best performance based on the total infected epidemiological metric occurs at the worst values for conventional performance metric of classification algorithms, i.e., low Fowlkes Mallows index, due to a high false discovery rate.*

# Medians and Inter Quartile Ranges Realistic Behaviour Optimal behaviour

|  |  |  |  | **Generation interval** | | **Serial interval** | | **7-day incidence** | | **7-day hosp. incidence** | | **Total infected** | | **Wave duration** | | **Days in quarantine** | |
| --- | --- | --- | --- | --- | --- | --- | --- | --- | --- | --- | --- | --- | --- | --- | --- | --- | --- |
|  |  |  |  | **Median** | **IQR** | **Median** | **IQR** | **Median** | **IQR** | **Median** | **IQR** | **Median** | **IQR** | **Median** | **IQR** | **Median** | **IQR** |
| **DCT** | **RNT** | **QCH** | **CBR** |  |  |  |  |  |  |  |  |  |  |  |  |  |  |
| **NO DCT** | **NO RNT** | **NO QCH** | **NO CBR** | 4.9 | [3.1, 6.9] | 5.1 | [3.1, 7.2] | 13.2 | [12.0, 16.2] | 3.2 | [2.7, 3.7] | 651.5 | [578.0, 689.5] | 121.2 | [104.4, 137.6] | 24.7 | [21.8, 28.0] |
| **DCT** | **NO RNT** | **NO QCH** | **NO CBR** | 4.5 | [3.0, 6.3] | 4.7 | [2.8, 6.7] | 2.9 | [2.3, 3.9] | 0.6 | [0.4, 0.9] | 43.5 | [30.0, 65.8] | 36.1 | [31.3, 41.8] | 14.0 | [14.0, 14.0] |
|  |  |  | **CBR** | 4.6 | [2.9, 6.5] | 4.8 | [2.7, 6.7] | 2.7 | [1.8, 3.7] | 0.6 | [0.4, 0.9] | 35.0 | [25.2, 55.8] | 31.9 | [28.7, 39.7] | 14.0 | [14.0, 14.0] |
|  |  | **QCH** | **NO CBR** | 4.6 | [2.9, 6.5] | 4.7 | [2.5, 6.8] | 2.9 | [2.0, 3.6] | 0.7 | [0.3, 1.0] | 38.0 | [24.0, 54.8] | 33.2 | [30.4, 38.9] | 14.0 | [14.0, 14.0] |
|  |  |  | **CBR** | 4.2 | [2.8, 6.0] | 4.7 | [2.7, 6.7] | 2.7 | [2.0, 4.0] | 0.7 | [0.4, 1.0] | 39.0 | [25.2, 50.0] | 33.5 | [29.7, 37.4] | 14.0 | [14.0, 14.0] |
|  | **RNT** | **NO QCH** | **NO CBR** | 4.4 | [3.0, 6.4] | 4.8 | [2.8, 6.8] | 2.9 | [2.0, 3.8] | 0.7 | [0.5, 0.9] | 42.0 | [28.5, 65.0] | 34.0 | [29.3, 41.5] | 4.1 | [4.0, 5.0] |
|  |  |  | **CBR** | 4.4 | [2.9, 6.3] | 4.9 | [2.8, 6.9] | 2.9 | [2.2, 4.8] | 0.8 | [0.4, 1.1] | 49.0 | [30.5, 67.5] | 36.1 | [30.9, 48.1] | 4.1 | [4.0, 5.0] |
|  |  | **QCH** | **NO CBR** | 4.3 | [2.9, 6.1] | 4.7 | [2.7, 6.7] | 2.8 | [1.9, 4.1] | 0.7 | [0.6, 1.0] | 39.5 | [24.5, 68.8] | 34.1 | [28.2, 44.9] | 4.1 | [4.0, 5.1] |
|  |  |  | **CBR** | 4.6 | [2.9, 6.4] | 4.8 | [2.8, 6.8] | 2.6 | [2.0, 3.4] | 0.6 | [0.3, 1.0] | 39.5 | [25.2, 69.5] | 35.4 | [27.8, 55.8] | 4.1 | [4.0, 5.0] |

*Table S11:* *Median and IQR of pandemic characteristics for optimal behaviour. This table complements Figure 5 from the main document. In DCT’s optimal behaviour, we observe that DCT is the main contributor to the reduction of the pandemic characteristics. The only clear differentiator is the use of DCT and RNT and its effect on days in quarantine. For other combinations, the IQRs overlap indicates that additional pandemic characteristics might be necessary when selecting NPI combinations.*

# Medians and Inter Quartile Ranges Realistic Behaviour

|  |  |  |  | **Generation interval** | | **Serial interval** | | **7-day incidence** | | **7-day hosp. incidence** | | **Total infected** | | **Wave duration** | | **Days in quarantine** | |
| --- | --- | --- | --- | --- | --- | --- | --- | --- | --- | --- | --- | --- | --- | --- | --- | --- | --- |
|  |  |  |  | **Median** | **IQR** | **Median** | **IQR** | **Median** | **IQR** | **Median** | **IQR** | **Median** | **IQR** | **Median** | **IQR** | **Median** | **IQR** |
| **DCT** | **RNT** | **QCH** | **CBR** |  |  |  |  |  |  |  |  |  |  |  |  |  |  |
| **NO DCT** | **NO RNT** | **NO QCH** | **NO CBR** | 4.9 | [3.1, 6.9] | 5.1 | [3.1, 7.2] | 15.7 | [12.4, 17.7] | 3.6 | [2.9, 4.1] | 660.0 | [592.0, 708.0] | 107.3 | [94.0, 130.8] | 24.6 | [22.0, 27.5] |
|  |  |  | **CBR** | 4.9 | [3.1, 7.0] | 5.2 | [3.1, 7.3] | 11.9 | [10.0, 14.4] | 2.9 | [2.3, 3.6] | 633.0 | [538.5, 679.8] | 122.6 | [107.9, 144.1] | 24.7 | [22.1, 28.0] |
|  |  | **QCH** | **NO CBR** | 4.9 | [3.1, 7.0] | 5.2 | [3.1, 7.3] | 14.4 | [11.4, 18.0] | 3.4 | [2.8, 3.9] | 641.0 | [582.0, 684.5] | 99.9 | [89.7, 120.9] | 24.6 | [22.0, 27.3] |
|  |  |  | **CBR** | 4.9 | [3.1, 7.0] | 5.2 | [3.1, 7.3] | 13.6 | [10.7, 17.7] | 3.1 | [2.6, 4.0] | 665.5 | [605.5, 699.8] | 119.9 | [98.6, 138.2] | 24.7 | [22.0, 28.0] |
|  | **RNT** | **NO QCH** | **NO CBR** | 4.9 | [3.1, 6.9] | 5.2 | [3.1, 7.3] | 16.2 | [14.0, 19.5] | 3.8 | [3.1, 4.5] | 707.0 | [657.5, 749.0] | 109.3 | [100.6, 123.6] | 5.0 | [2.8, 8.7] |
|  |  |  | **CBR** | 4.9 | [3.1, 6.9] | 5.2 | [3.1, 7.2] | 16.5 | [14.6, 18.5] | 3.7 | [3.1, 4.4] | 700.5 | [663.0, 752.2] | 112.9 | [97.7, 126.3] | 5.0 | [2.8, 8.7] |
|  |  | **QCH** | **NO CBR** | 4.9 | [3.1, 6.9] | 5.1 | [3.1, 7.2] | 16.3 | [13.8, 19.7] | 3.6 | [3.0, 4.4] | 699.5 | [661.0, 747.8] | 118.0 | [103.4, 132.6] | 4.9 | [2.8, 8.8] |
|  |  |  | **CBR** | 4.9 | [3.1, 6.9] | 5.2 | [3.1, 7.3] | 14.8 | [11.2, 18.8] | 3.3 | [2.8, 4.3] | 695.5 | [629.5, 739.5] | 118.5 | [101.5, 133.0] | 4.9 | [2.9, 8.6] |
| **DCT** | **NO RNT** | **NO QCH** | **NO CBR** | 4.8 | [3.1, 6.8] | 5.0 | [2.9, 7.1] | 8.6 | [5.9, 10.1] | 2.0 | [1.6, 2.4] | 379.5 | [260.8, 451.0] | 109.3 | [79.9, 130.9] | 24.9 | [14.0, 35.5] |
|  |  |  | **CBR** | 4.8 | [3.0, 6.8] | 5.0 | [3.0, 7.1] | 8.3 | [5.2, 10.6] | 1.8 | [1.2, 2.4] | 328.5 | [109.8, 432.5] | 93.2 | [55.7, 125.5] | 24.3 | [14.0, 28.0] |
|  |  | **QCH** | **NO CBR** | 4.8 | [3.0, 6.8] | 5.0 | [3.0, 7.2] | 7.2 | [4.8, 9.8] | 1.7 | [1.3, 2.3] | 300.0 | [176.8, 410.0] | 92.0 | [76.1, 127.0] | 24.1 | [14.0, 28.0] |
|  |  |  | **CBR** | 4.8 | [3.0, 6.8] | 5.1 | [3.0, 7.1] | 8.4 | [5.7, 10.9] | 1.9 | [1.3, 2.6] | 386.0 | [203.0, 471.0] | 107.8 | [92.2, 139.1] | 24.4 | [14.0, 28.0] |
|  | **RNT** | **NO QCH** | **NO CBR** | 4.8 | [3.1, 6.9] | 5.1 | [3.0, 7.2] | 9.2 | [6.4, 12.8] | 2.1 | [1.6, 2.9] | 476.0 | [259.8, 570.8] | 112.7 | [90.0, 138.6] | 5.9 | [4.0, 10.3] |
|  |  |  | **CBR** | 4.8 | [3.0, 6.8] | 5.0 | [3.0, 7.1] | 9.0 | [7.4, 11.1] | 2.3 | [1.6, 2.8] | 486.0 | [361.0, 559.0] | 122.1 | [94.4, 135.4] | 5.9 | [4.0, 10.3] |
|  |  | **QCH** | **NO CBR** | 4.9 | [3.1, 6.9] | 5.1 | [3.1, 7.2] | 10.0 | [6.1, 12.8] | 2.4 | [1.4, 3.0] | 514.0 | [336.8, 591.0] | 117.7 | [99.2, 135.5] | 5.9 | [4.0, 10.4] |
|  |  |  | **CBR** | 4.9 | [3.1, 6.9] | 5.1 | [3.1, 7.2] | 9.6 | [6.9, 12.1] | 2.3 | [1.6, 2.7] | 463.5 | [356.0, 555.5] | 126.3 | [99.2, 144.3] | 5.9 | [4.0, 10.2] |

*Table S12:* *Median and IQR of pandemic characteristics for realistic behaviour. This table complements Figure 5 from the main document. In realistic behaviour, we observe that DCT is the main driver of pandemic characteristic reduction, and in combination with RNT, provides strong differentiation for days in quarantine. Using the IQR, we observe that DCT and No RNT yield the best performance group. However, selecting between combinations of QCH and CBR based on the mean and median shows that additional information is required for an adequate selection.*

# Pandemic characteristics difference of medians with 95% Confidence Intervals for Optimal Behaviour

## Total Infected

| **Total infected** | | | | | | | | | | | |
| --- | --- | --- | --- | --- | --- | --- | --- | --- | --- | --- | --- |
|  | | | **DCT** | **Dct** | | | | | | | |
|  |  |  | **RNT** | **No Rnt** | | | | **Rnt** | | | |
|  |  |  | **QCH** | **No Qch** | | **Qch** | | **No Qch** | | **Qch** | |
|  |  |  | **CBR** | **No Cbr** | **Cbr** | **No Cbr** | **Cbr** | **No Cbr** | **Cbr** | **No Cbr** | **Cbr** |
| **DCT** | **RNT** | **QCH** | **CBR** |  |  |  |  |  |  |  |  |
| **No Dct** | **No Rnt** | **No Qch** | **No Cbr** | -608 [-633, -561] | -616 [-639, -574] | -613 [-636, -573] | -612 [-637, -570] | -609 [-634, -569] | -602 [-631, -560] | -612 [-635, -569] | -612 [-635, -569] |
| **Dct** | **No Rnt** | **No Qch** | **No Cbr** |  | -8.50 [-26.0, 3.00] | -5.50 [-23.0, 4.00] | -4.50 [-25.0, 6.50] | -1.50 [-20.5, 10.0] | 5.50 [-17.5, 19.0] | -4.00 [-20.5, 12.0] | -4.00 [-24.0, 9.00] |
|  |  |  | **Cbr** |  |  | 3.00 [-5.50, 9.51] | 4.00 [-7.50, 11.5] | 7.00 [-4.00, 16.0] | 14.0 [-1.50, 24.0] | 4.50 [-4.00, 18.0] | 4.50 [-5.51, 16.0] |
|  |  | **Qch** | **No Cbr** |  |  |  | 1.00 [-9.00, 8.00] | 4.00 [-5.00, 14.0] | 11.0 [-3.50, 22.0] | 1.50 [-4.00, 14.0] | 1.50 [-8.00, 12.0] |
|  |  |  | **Cbr** |  |  |  |  | 3.00 [-7.00, 14.0] | 10.0 [-5.50, 24.0] | 0.50 [-8.00, 16.0] | 0.50 [-10.5, 14.5] |
|  | **Rnt** | **No Qch** | **No Cbr** |  |  |  |  |  | 7.00 [-8.50, 19.5] | -2.50 [-12.5, 13.5] | -2.50 [-14.0, 11.0] |
|  |  |  | **Cbr** |  |  |  |  |  |  | -9.50 [-20.5, 11.5] | -9.50 [-22.0, 7.51] |
|  |  | **Qch** | **No Cbr** |  |  |  |  |  |  |  | 0.00 [-15.0, 11.0] |
|  |  |  | **Cbr** |  |  |  |  |  |  |  |  |

*Table S13:* *Difference of median with 95% CI for the total infected population for all NPI configurations. A negative difference refers to an improvement from configurations in the columns with respect to the row, whereas a positive CI marks an improvement of the row configuration over the column. There is a significant difference (green) in the median of total infected between any configuration with DCT and and only the default NPI configuration. However, there are no significant differences between any of the configurations using DCT.*

## Max. 7-day Hosp. Incidence

| **Max. 7-day Hosp. Incidence [d]** | | | | | | | | | | | |
| --- | --- | --- | --- | --- | --- | --- | --- | --- | --- | --- | --- |
|  | | | **DCT** | **Dct** | | | | | | | |
|  |  |  | **RNT** | **No Rnt** | | | | **Rnt** | | | |
|  |  |  | **QCH** | **No Qch** | | **Qch** | | **No Qch** | | **Qch** | |
|  |  |  | **CBR** | **No Cbr** | **Cbr** | **No Cbr** | **Cbr** | **No Cbr** | **Cbr** | **No Cbr** | **Cbr** |
| **DCT** | **RNT** | **QCH** | **CBR** |  |  |  |  |  |  |  |  |
| **No Dct** | **No Rnt** | **No Qch** | **No Cbr** | -2.64 [-2.86, -2.29] | -2.57 [-2.93, -2.29] | -2.50 [-2.86, -2.14] | -2.50 [-2.86, -2.21] | -2.50 [-2.71, -2.21] | -2.43 [-2.79, -2.07] | -2.50 [-2.71, -2.07] | -2.64 [-2.93, -2.29] |
| **Dct** | **No Rnt** | **No Qch** | **No Cbr** |  | 0.07 [-0.21, 0.14] | 0.14 [-0.14, 0.29] | 0.14 [-0.14, 0.29] | 0.14 [0.00, 0.21] | 0.21 [-0.14, 0.43] | 0.14 [0.00, 0.36] | 0.00 [-0.29, 0.14] |
|  |  |  | **Cbr** |  |  | 0.07 [-0.21, 0.43] | 0.07 [-0.21, 0.29] | 0.07 [-0.07, 0.29] | 0.14 [-0.14, 0.43] | 0.07 [-0.07, 0.43] | -0.07 [-0.29, 0.21] |
|  |  | **Qch** | **No Cbr** |  |  |  | 0.00 [-0.29, 0.29] | 0.00 [-0.14, 0.29] | 0.07 [-0.29, 0.43] | 0.00 [-0.14, 0.36] | -0.14 [-0.43, 0.14] |
|  |  |  | **Cbr** |  |  |  |  | 0.00 [-0.14, 0.29] | 0.07 [-0.14, 0.36] | 0.00 [-0.14, 0.36] | -0.14 [-0.36, 0.14] |
|  | **Rnt** | **No Qch** | **No Cbr** |  |  |  |  |  | 0.07 [-0.21, 0.29] | 0.00 [-0.14, 0.29] | -0.14 [-0.29, 0.00] |
|  |  |  | **Cbr** |  |  |  |  |  |  | -0.07 [-0.21, 0.29] | -0.21 [-0.43, 0.07] |
|  |  | **Qch** | **No Cbr** |  |  |  |  |  |  |  | -0.14 [-0.43, 0.00] |
|  |  |  | **Cbr** |  |  |  |  |  |  |  |  |

*Table S14:* *Difference of median with 95% CI for the “max. 7-day Hosp. Incidence” for all NPI configurations. A negative difference refers to an improvement from configurations in the columns with respect to the row, whereas a positive CI marks an improvement of the row configuration over the column. There is a significant difference (green) in the median of “max. 7-day Hosp. Incidence” between any configuration with DCT and only the default NPI.*

## Max. 7-day Incidence

| **Max. 7-day Incidence** | | | | | | | | | | | |
| --- | --- | --- | --- | --- | --- | --- | --- | --- | --- | --- | --- |
|  | | | **DCT** | **Dct** | | | | | | | |
|  |  |  | **RNT** | **No Rnt** | | | | **Rnt** | | | |
|  |  |  | **QCH** | **No Qch** | | **Qch** | | **No Qch** | | **Qch** | |
|  |  |  | **CBR** | **No Cbr** | **Cbr** | **No Cbr** | **Cbr** | **No Cbr** | **Cbr** | **No Cbr** | **Cbr** |
| **DCT** | **RNT** | **QCH** | **CBR** |  |  |  |  |  |  |  |  |
| **No Dct** | **No Rnt** | **No Qch** | **No Cbr** | -10.3 [-11.7, -9.43] | -10.5 [-11.9, -9.64] | -10.4 [-11.6, -9.57] | -10.5 [-12.1, -9.57] | -10.4 [-11.8, -9.57] | -10.4 [-11.9, -9.14] | -10.4 [-11.7, -9.21] | -10.6 [-12.1, -9.64] |
| **Dct** | **No Rnt** | **No Qch** | **No Cbr** |  | -0.21 [-1.00, 0.50] | -0.07 [-0.57, 0.43] | -0.21 [-0.79, 0.36] | -0.07 [-0.79, 0.36] | -0.07 [-0.57, 1.00] | -0.14 [-0.79, 0.93] | -0.29 [-0.86, 0.36] |
|  |  |  | **Cbr** |  |  | 0.14 [-0.57, 0.93] | 0.00 [-0.71, 0.86] | 0.14 [-0.57, 0.93] | 0.14 [-0.50, 1.36] | 0.07 [-0.57, 1.29] | -0.07 [-0.71, 0.86] |
|  |  | **Qch** | **No Cbr** |  |  |  | -0.14 [-0.71, 0.43] | 0.00 [-0.64, 0.43] | 0.00 [-0.43, 1.00] | -0.07 [-0.57, 0.86] | -0.21 [-0.71, 0.36] |
|  |  |  | **Cbr** |  |  |  |  | 0.14 [-0.64, 0.64] | 0.14 [-0.43, 1.14] | 0.07 [-0.57, 1.00] | -0.07 [-0.64, 0.57] |
|  | **Rnt** | **No Qch** | **No Cbr** |  |  |  |  |  | 0.00 [-0.43, 1.14] | -0.07 [-0.50, 1.00] | -0.21 [-0.57, 0.50] |
|  |  |  | **Cbr** |  |  |  |  |  |  | -0.07 [-1.14, 0.86] | -0.21 [-1.21, 0.36] |
|  |  | **Qch** | **No Cbr** |  |  |  |  |  |  |  | -0.14 [-1.00, 0.43] |
|  |  |  | **Cbr** |  |  |  |  |  |  |  |  |

*Table S15:* *Difference of median with 95% CI for the “max. 7-day Incidence” for all NPI configurations. A negative difference refers to an improvement from configurations in the columns with respect to the row, whereas a positive CI marks an improvement of the row configuration over the column. There is a significant difference (green) in the median of “max. 7-day Incidence” between any configuration with DCT and only the default NPI.*

## Wave Duration

| ***Wave Duration [d]*** | | | | | | | | | | | |
| --- | --- | --- | --- | --- | --- | --- | --- | --- | --- | --- | --- |
|  | | | ***DCT*** | ***Dct*** | | | | | | | |
|  |  |  | ***RNT*** | ***No Rnt*** | | | | ***Rnt*** | | | |
|  |  |  | ***QCH*** | ***No Qch*** | | ***Qch*** | | ***No Qch*** | | ***Qch*** | |
|  |  |  | ***CBR*** | ***No Cbr*** | ***Cbr*** | ***No Cbr*** | ***Cbr*** | ***No Cbr*** | ***Cbr*** | ***No Cbr*** | ***Cbr*** |
| ***DCT*** | ***RNT*** | ***QCH*** | ***CBR*** |  |  |  |  |  |  |  |  |
| ***No Dct*** | ***No Rnt*** | ***No Qch*** | ***No Cbr*** | *-85.1 [-96.6, -76.3]* | *-89.3 [-100, -79.8]* | *-88.0 [-99.5, -79.2]* | *-87.7 [-99.1, -80.8]* | *-87.1 [-98.4, -77.8]* | *-85.1 [-96.9, -77.2]* | *-87.1 [-98.9, -77.8]* | *-85.8 [-97.2, -75.8]* |
| ***Dct*** | ***No Rnt*** | ***No Qch*** | ***No Cbr*** |  | *-4.19 [-7.31, -0.06]* | *-2.87 [-5.92, 0.84]* | *-2.63 [-5.44, -0.70]* | *-2.06 [-5.30, 2.42]* | *-0.00 [-4.01, 4.48]* | *-2.00 [-5.90, 1.96]* | *-0.70 [-4.35, 6.83]* |
|  |  |  | ***Cbr*** |  |  | *1.33 [-3.16, 4.52]* | *1.56 [-2.34, 3.51]* | *2.13 [-1.74, 7.21]* | *4.19 [-0.92, 9.62]* | *2.19 [-2.53, 6.00]* | *3.49 [-0.51, 10.1]* |
|  |  | ***Qch*** | ***No Cbr*** |  |  |  | *0.24 [-3.20, 2.18]* | *0.81 [-2.10, 5.32]* | *2.87 [-0.82, 7.63]* | *0.87 [-2.96, 5.20]* | *2.16 [-1.25, 9.52]* |
|  |  |  | ***Cbr*** |  |  |  |  | *0.57 [-1.59, 5.29]* | *2.63 [-0.58, 7.76]* | *0.63 [-2.75, 5.64]* | *1.93 [-0.77, 8.83]* |
|  | ***Rnt*** | ***No Qch*** | ***No Cbr*** |  |  |  |  |  | *2.06 [-3.33, 6.58]* | *0.06 [-5.35, 4.10]* | *1.36 [-3.64, 7.27]* |
|  |  |  | ***Cbr*** |  |  |  |  |  |  | *-2.00 [-7.30, 2.64]* | *-0.70 [-5.31, 7.57]* |
|  |  | ***Qch*** | ***No Cbr*** |  |  |  |  |  |  |  | *1.30 [-3.28, 9.39]* |
|  |  |  | ***Cbr*** |  |  |  |  |  |  |  |  |

*Table S16:* *Difference of median with 95% CI for the “wave duration” for all NPI configurations. A negative difference refers to an improvement from configurations in the columns with respect to the row, whereas a positive CI marks an improvement of the row configuration over the column. There is a significant difference (green) in the median of “wave duration” between any configuration with DCT and only the default NPI. While adding CBR seems to do better than only DCT, the difference is not practically relevant.*

| ***Days in Quarantine [d]*** | | | | | | | | | | | |
| --- | --- | --- | --- | --- | --- | --- | --- | --- | --- | --- | --- |
|  | | | ***DCT*** | ***Dct*** | | | | | | | |
|  |  |  | ***RNT*** | ***No Rnt*** | | | | ***Rnt*** | | | |
|  |  |  | ***QCH*** | ***No Qch*** | | ***Qch*** | | ***No Qch*** | | ***Qch*** | |
|  |  |  | ***CBR*** | ***No Cbr*** | ***Cbr*** | ***No Cbr*** | ***Cbr*** | ***No Cbr*** | ***Cbr*** | ***No Cbr*** | ***Cbr*** |
| ***DCT*** | ***RNT*** | ***QCH*** | ***CBR*** |  |  |  |  |  |  |  |  |
| ***No Dct*** | ***No Rnt*** | ***No Qch*** | ***No Cbr*** | *-10.8 [-11.6, -9.77]* | *-10.8 [-11.7, -9.77]* | *-10.8 [-11.7, -9.70]* | *-10.8 [-11.7, -9.77]* | *-20.6 [-21.4, -19.3]* | *-20.8 [-21.7, -19.7]* | *-20.7 [-21.4, -19.5]* | *-20.8 [-21.6, -19.7]* |
| ***Dct*** | ***No Rnt*** | ***No Qch*** | ***No Cbr*** |  | *0.00 [0.00, 0.00]* | *0.00 [0.00, 0.00]* | *0.00 [0.00, 0.00]* | *-9.77 [-9.96, -9.17]* | *-9.99 [-10.0, -9.92]* | *-9.90 [-9.97, -9.27]* | *-9.97 [-9.99, -9.87]* |
|  |  |  | ***Cbr*** |  |  | *0.00 [0.00, 0.00]* | *0.00 [0.00, 0.00]* | *-9.77 [-9.96, -9.17]* | *-9.99 [-10.0, -9.91]* | *-9.90 [-9.97, -9.27]* | *-9.97 [-9.99, -9.87]* |
|  |  | ***Qch*** | ***No Cbr*** |  |  |  | *0.00 [0.00, 0.00]* | *-9.77 [-9.98, -9.17]* | *-9.99 [-10.0, -9.92]* | *-9.90 [-9.97, -9.27]* | *-9.97 [-9.99, -9.85]* |
|  |  |  | ***Cbr*** |  |  |  |  | *-9.77 [-9.98, -9.13]* | *-9.99 [-10.0, -9.92]* | *-9.90 [-9.97, -9.27]* | *-9.97 [-10.00, -9.86]* |
|  | ***Rnt*** | ***No Qch*** | ***No Cbr*** |  |  |  |  |  | *-0.22 [-0.85, 0.00]* | *-0.12 [-0.75, 0.52]* | *-0.19 [-0.81, 0.01]* |
|  |  |  | ***Cbr*** |  |  |  |  |  |  | *0.09 [-0.02, 0.72]* | *0.02 [-0.05, 0.13]* |
|  |  | ***Qch*** | ***No Cbr*** |  |  |  |  |  |  |  | *-0.07 [-0.70, 0.05]* |
|  |  |  | ***Cbr*** |  |  |  |  |  |  |  |  |

*Table S17:* *Difference of median with 95% CI for the “Days in Quarantine” for all NPI configurations. A negative difference refers to an improvement from configurations in the columns with respect to the row, whereas a positive CI marks an improvement of the row configuration over the column. There is a significant difference (green) in the median of “Days in Quarantine” between any configuration with DCT and only the default NPI. In addition, there is a significant difference of median when using RNT, which reduces the Days in quarantine by ~10 days. Applying the remaining NPI does not achieve any significant difference.*

## Generation Interval

| **Generation Interval [d]** | | | | | | | | | | | |
| --- | --- | --- | --- | --- | --- | --- | --- | --- | --- | --- | --- |
|  | | | **DCT** | **Dct** | | | | | | | |
|  |  |  | **RNT** | **No Rnt** | | | | **Rnt** | | | |
|  |  |  | **QCH** | **No Qch** | | **Qch** | | **No Qch** | | **Qch** | |
|  |  |  | **CBR** | **No Cbr** | **Cbr** | **No Cbr** | **Cbr** | **No Cbr** | **Cbr** | **No Cbr** | **Cbr** |
| **DCT** | **RNT** | **QCH** | **CBR** |  |  |  |  |  |  |  |  |
| **No Dct** | **No Rnt** | **No Qch** | **No Cbr** | -0.81 [-2.58, -0.16] | -0.99 [-2.70, 0.04] | -0.91 [-2.60, 0.33] | -0.75 [-2.60, 0.25] | 0.32 [-1.34, 1.33] | -1.10 [-3.11, 0.13] | -1.52  [-3.12, -0.12] | -1.16  [-2.82, -0.29] |
| **Dct** | **No Rnt** | **No Qch** | **No Cbr** |  | -0.18 [-1.38, 1.35] | -0.10 [-1.14, 1.37] | 0.06 [-1.45, 1.36] | 1.13 [0.00, 2.56] | -0.29  [-1.65, 1.39] | -0.72 [-1.79, 1.19] | -0.35 [-1.42, 1.10] |
|  |  |  | **Cbr** |  |  | 0.08 [-1.30, 1.55] | 0.24 [-1.32, 1.44] | 1.32 [0.04, 2.65] | -0.11  [-1.77, 1.46] | -0.53 [-1.67, 1.25] | -0.17 [-1.44, 1.14] |
|  |  | **Qch** | **No Cbr** |  |  |  | 0.16 [-1.51, 1.44] | 1.24 [-0.14, 2.64] | -0.19  [-2.02, 1.30] | -0.61 [-1.86, 1.17] | -0.25 [-1.58, 1.12] |
|  |  |  | **Cbr** |  |  |  |  | 1.07 [-0.05, 2.65] | -0.35  [-1.98, 1.45] | -0.78 [-1.85, 1.40] | -0.41 [-1.50, 1.18] |
|  | **Rnt** | **No Qch** | **No Cbr** |  |  |  |  |  | -1.42  [-3.12, 0.06] | -1.85  [-2.98, -0.07] | -1.48  [-2.71, -0.26] |
|  |  |  | **Cbr** |  |  |  |  |  |  | -0.43 [-1.79, 1.71] | -0.06 [-1.36, 1.60] |
|  |  | **Qch** | **No Cbr** |  |  |  |  |  |  |  | 0.36 [-1.34, 1.52] |
|  |  |  | **Cbr** |  |  |  |  |  |  |  |  |

*Table S18:* *Difference of median with 95% CI for the “Generation Interval ” for all NPI configurations. A negative difference refers to an improvement from configurations in the columns with respect to the row, whereas a positive CI marks an improvement of the row configuration over the column. The significant statistical differences (green) from applying any NPI combination on top of the default NPI are considered not practical differences.*

## Serial Interval

| **Serial Interval [d]** | | | | | | | | | | | |
| --- | --- | --- | --- | --- | --- | --- | --- | --- | --- | --- | --- |
|  | | | **DCT** | **Dct** | | | | | | | |
|  |  |  | **RNT** | **No Rnt** | | | | **Rnt** | | | |
|  |  |  | **QCH** | **No Qch** | | **Qch** | | **No Qch** | | **Qch** | |
|  |  |  | **CBR** | **No Cbr** | **Cbr** | **No Cbr** | **Cbr** | **No Cbr** | **Cbr** | **No Cbr** | **Cbr** |
| **DCT** | **RNT** | **QCH** | **CBR** |  |  |  |  |  |  |  |  |
| **No Dct** | **No Rnt** | **No Qch** | **No Cbr** | 0.20 [-1.17, 1.70] | 0.77 [-0.45, 1.98] | 0.16 [-1.25, 2.22] | 0.31 [-1.62, 2.01] | 0.28 [-0.98, 1.80] | 0.52 [-0.70, 2.05] | 0.18 [-1.26, 1.35] | 0.86 [-0.58, 1.82] |
| **Dct** | **No Rnt** | **No Qch** | **No Cbr** |  | 0.57 [-0.98, 1.99] | -0.04 [-1.65, 2.12] | 0.11 [-2.19, 2.08] | 0.08 [-1.47, 1.60] | 0.32 [-1.27, 1.94] | -0.02 [-2.01, 1.29] | 0.66 [-1.17, 1.84] |
|  |  |  | **Cbr** |  |  | -0.61 [-1.89, 1.62] | -0.46 [-2.54, 1.24] | -0.49 [-1.78, 1.15] | -0.25 [-1.53, 1.26] | -0.59 [-2.14, 0.64] | 0.09 [-1.39, 1.22] |
|  |  | **Qch** | **No Cbr** |  |  |  | 0.15 [-2.34, 1.93] | 0.12 [-1.92, 1.67] | 0.36 [-1.76, 1.70] | 0.02 [-2.14, 1.30] | 0.70 [-1.69, 1.76] |
|  |  |  | **Cbr** |  |  |  |  | -0.03 [-1.83, 2.43] | 0.21 [-1.47, 2.23] | -0.13 [-2.12, 1.72] | 0.55 [-1.32, 2.41] |
|  | **Rnt** | **No Qch** | **No Cbr** |  |  |  |  |  | 0.24 [-1.25, 1.72] | -0.10 [-2.01, 1.22] | 0.58 [-1.19, 1.72] |
|  |  |  | **Cbr** |  |  |  |  |  |  | -0.34 [-2.08, 0.90] | 0.34 [-1.26, 1.53] |
|  |  | **Qch** | **No Cbr** |  |  |  |  |  |  |  | 0.68 [-0.67, 2.11] |
|  |  |  | **Cbr** |  |  |  |  |  |  |  |  |

*Table S19:* *Difference of median with 95% CI for the “Serial Interval ” for all NPI configurations. A negative difference refers to an improvement from configurations in the columns with respect to the row, whereas a positive CI marks an improvement of the row configuration over the column. There are no practical statistical significant differences from applying any NPI combination on top of the default NPI.*

# Pandemic characteristics difference of medians with 95% Confidence Intervals for Realistic Behaviour

## Total Infected

| **Total infected** | | | | | | | | | | | | | | | | | | |
| --- | --- | --- | --- | --- | --- | --- | --- | --- | --- | --- | --- | --- | --- | --- | --- | --- | --- | --- |
|  | | | **DCT** |  |  |  |  |  |  |  | **Dct** | | | | | | | |
|  |  |  | **RNT** |  |  |  | **Rnt** | | | | **No Rnt** | | | | **Rnt** | | | |
|  |  |  | **QCH** |  | **Qch** | | **No Qch** | | **Qch** | | **No Qch** | | **Qch** | | **No Qch** | | **Qch** | |
|  |  |  | **CBR** | **Cbr** | **No Cbr** | **Cbr** | **No Cbr** | **Cbr** | **No Cbr** | **Cbr** | **No Cbr** | **Cbr** | **No Cbr** | **Cbr** | **No Cbr** | **Cbr** | **No Cbr** | **Cbr** |
| **DCT** | **RNT** | **QCH** | **CBR** |  |  |  |  |  |  |  |  |  |  |  |  |  |  |  |
| **No Dct** | **No Rnt** | **No Qch** | **No Cbr** | -27.0 [-92.0, 19.0] | -19.0 [-56.0, 33.5] | 5.50 [-31.0, 48.5] | 47.0 [6.99, 97.0] | 40.5 [3.00, 90.0] | 39.5 [4.49, 84.5] | 35.5 [-6.01, 80.5] | -280 [-315, -229] | -331 [-405, -263] | -360 [-408, -296] | -274 [-358, -223] | -184 [-286, -104] | -174 [-250, -127] | -146 [-242, -97.0] | -196 [-234, -124] |
|  |  |  | **Cbr** |  | 8.00 [-30.5, 74.5] | 32.5 [-6.51, 87.0] | 74.0 [30.0, 137] | 67.5 [22.5, 132] | 66.5 [31.5, 134] | 62.5 [21.9, 123] | -253 [-291, -173] | -304 [-379, -203] | -333 [-383, -252] | -247 [-337, -186] | -157 [-253, -55.5] | -147 [-221, -78.5] | -119 [-205, -54.5] | -169 [-210, -75.5] |
|  |  | **Qch** | **No Cbr** |  |  | 24.5 [-17.5, 57.5] | 66.0 [25.5, 101] | 59.5 [16.5, 98.5] | 58.5 [20.0, 98.0] | 54.5 [9.98, 86.5] | -261 [-302, -211] | -312 [-386, -246] | -341 [-391, -280] | -255 [-343, -221] | -165 [-262, -84.5] | -155 [-231, -114] | -127 [-224, -90.5] | -177 [-219, -109] |
|  |  |  | **Cbr** |  |  |  | 41.5 [8.00, 79.0] | 35.0 [3.50, 77.5] | 34.0 [7.49, 74.0] | 30.0 [-6.00, 66.0] | -286 [-317, -230] | -337 [-405, -266] | -365 [-411, -304] | -279 [-354, -248] | -189 [-278, -106] | -179 [-252, -135] | -151 [-227, -114] | -202 [-232, -127] |
|  | **Rnt** | **No Qch** | **No Cbr** |  |  |  |  | -6.50 [-45.0, 34.5] | -7.50 [-38.0, 27.5] | -11.5 [-53.0, 23.5] | -327 [-361, -269] | -378 [-457, -308] | -407 [-459, -350] | -321 [-408, -285] | -231 [-320, -150] | -221 [-295, -177] | -193 [-291, -158] | -243 [-280, -179] |
|  |  |  | **Cbr** |  |  |  |  |  | -1.00 [-35.0, 40.0] | -5.00 [-48.0, 31.0] | -321 [-366, -268] | -372 [-452, -299] | -400 [-452, -334] | -314 [-402, -276] | -224 [-324, -142] | -214 [-292, -169] | -186 [-276, -145] | -237 [-283, -168] |
|  |  | **Qch** | **No Cbr** |  |  |  |  |  |  | -4.00 [-50.0, 25.5] | -320 [-357, -267] | -371 [-451, -301] | -399 [-451, -340] | -313 [-395, -285] | -223 [-314, -144] | -213 [-295, -175] | -185 [-280, -149] | -236 [-276, -169] |
|  |  |  | **Cbr** |  |  |  |  |  |  |  | -316 [-352, -262] | -367 [-443, -294] | -395 [-445, -327] | -309 [-395, -263] | -219 [-315, -135] | -209 [-282, -159] | -181 [-281, -136] | -232 [-268, -157] |
| **Dct** | **No Rnt** | **No Qch** | **No Cbr** |  |  |  |  |  |  |  |  | -51.0 [-142, 21.0] | -79.5 [-143, -19.5] | 6.50 [-81.0, 45.0] | 96.5 [1.45, 172] | 106 [17.5, 150] | 134 [27.9, 171] | 84.0 [26.0, 155] |
|  |  |  | **Cbr** |  |  |  |  |  |  |  |  |  | -28.5 [-103, 62.1] | 57.5 [-53.0, 121] | 147 [45.5, 255] | 157 [53.0, 224] | 185 [67.5, 254] | 135 [62.0, 242] |
|  |  | **Qch** | **No Cbr** |  |  |  |  |  |  |  |  |  |  | 86.0 [-10.5, 133] | 176 [81.4, 259] | 186 [92.0, 238] | 214 [117, 259] | 163 [105, 236] |
|  |  |  | **Cbr** |  |  |  |  |  |  |  |  |  |  |  | 90.0 [-4.50, 206] | 100 [27.9, 183] | 128 [39.9, 208] | 77.5 [44.0, 181] |
|  | **Rnt** | **No Qch** | **No Cbr** |  |  |  |  |  |  |  |  |  |  |  |  | 10.0 [-97.5, 101] | 38.0 [-89.1, 130] | -12.5 [-95.0, 107] |
|  |  |  | **Cbr** |  |  |  |  |  |  |  |  |  |  |  |  |  | 28.0 [-68.0, 98.0] | -22.5 [-63.5, 77.5] |
|  |  | **Qch** | **No Cbr** |  |  |  |  |  |  |  |  |  |  |  |  |  |  | -50.5 [-91.0, 62.0] |
|  |  |  | **Cbr** |  |  |  |  |  |  |  |  |  |  |  |  |  |  |  |

*Table S20:* *Difference of median with 95% CI for the “Total Infected” for all NPI configurations. A negative difference refers to an improvement from configurations in the columns with respect to the row, whereas a positive CI marks an improvement of the row configuration over the column. There is a statistical significant reduction in the “Total Infected” using DCT with any additional configuration (green). Using DCT + QCH has a statistically significant advantage over DCT. Combinations without RNT have statistically significant improvement in performance with respect to those with RNT (amber) with the exception of DCT + QCH + CBR, which has similar performance to DCT + RNT.*

## Max. 7-day Incidence

| **Max. 7-day Incidence** | | | | | | | | | | | | | | | | | | |
| --- | --- | --- | --- | --- | --- | --- | --- | --- | --- | --- | --- | --- | --- | --- | --- | --- | --- | --- |
|  | | | **DCT** | **No Dct** | | | | | | | **Dct** | | | | | | | |
|  |  |  | **RNT** |  |  |  | **Rnt** | | | | **No Rnt** | | | | **Rnt** | | | |
|  |  |  | **QCH** |  | **Qch** | | **No Qch** | | **Qch** | | **No Qch** | | **Qch** | | **No Qch** | | **Qch** | |
|  |  |  | **CBR** | **Cbr** | **No Cbr** | **Cbr** | **No Cbr** | **Cbr** | **No Cbr** | **Cbr** | **No Cbr** | **Cbr** | **No Cbr** | **Cbr** | **No Cbr** | **Cbr** | **No Cbr** | **Cbr** |
| **DCT** | **RNT** | **QCH** | **CBR** |  |  |  |  |  |  |  |  |  |  |  |  |  |  |  |
| **No Dct** | **No Rnt** | **No Qch** | **No Cbr** | -3.79 [-5.50, -1.57] | -1.36 [-3.21, 1.36] | -2.07 [-4.36, 1.71] | 0.50 [-1.50, 3.79] | 0.79 [-1.29, 2.79] | 0.57 [-1.43, 2.86] | -0.93 [-3.14, 2.14] | -7.14 [-8.86, -4.86] | -7.43 [-10.0, -5.14] | -8.50 [-10.3, -6.21] | -7.36 [-9.21, -5.14] | -6.50 [-8.93, -3.93] | -6.71 [-8.43, -4.57] | -5.71 [-7.79, -3.36] | -6.14 [-8.57, -4.00] |
|  |  |  | **Cbr** |  | 2.43 [0.86, 4.72] | 1.71 [-0.29, 4.86] | 4.29 [2.71, 6.86] | 4.57 [3.00, 5.57] | 4.36 [2.64, 5.79] | 2.86 [1.14, 5.36] | -3.36 [-4.86, -2.36] | -3.64 [-5.86, -2.50] | -4.71 [-6.00, -3.07] | -3.57 [-5.14, -2.50] | -2.71 [-4.64, -0.93] | -2.93 [-4.29, -2.07] | -1.93 [-3.29, -0.21] | -2.36 [-4.57, -1.00] |
|  |  | **Qch** | **No Cbr** |  |  | -0.71 [-3.36, 2.79] | 1.86 [-0.57, 4.43] | 2.14 [-0.43, 3.50] | 1.93 [-0.50, 3.79] | 0.43 [-2.43, 2.86] | -5.79 [-8.29, -4.43] | -6.07 [-8.93, -4.64] | -7.14 [-9.71, -5.50] | -6.00 [-8.29, -4.57] | -5.14 [-7.86, -3.29] | -5.36 [-7.93, -4.29] | -4.36 [-6.72, -2.43] | -4.79 [-7.64, -3.14] |
|  |  |  | **Cbr** |  |  |  | 2.57 [-0.86, 5.57] | 2.86 [-0.71, 4.57] | 2.64 [-0.72, 4.79] | 1.14 [-2.43, 4.07] | -5.07 [-8.57, -3.29] | -5.36 [-9.36, -3.50] | -6.43 [-9.79, -4.21] | -5.29 [-8.57, -3.57] | -4.43 [-8.07, -2.21] | -4.64 [-7.86, -3.00] | -3.64 [-7.00, -1.43] | -4.07 [-8.00, -2.00] |
|  | **Rnt** | **No Qch** | **No Cbr** |  |  |  |  | 0.29 [-2.71, 1.86] | 0.07 [-2.43, 2.00] | -1.43 [-4.36, 1.14] | -7.64 [-10.3, -6.14] | -7.93 [-10.9, -6.21] | -9.00 [-11.6, -7.07] | -7.86 [-10.4, -6.43] | -7.00 [-10.00, -5.00] | -7.21 [-9.57, -5.79] | -6.21 [-9.00, -4.21] | -6.64 [-9.57, -4.93] |
|  |  |  | **Cbr** |  |  |  |  |  | -0.21 [-1.71, 1.71] | -1.71 [-3.50, 1.29] | -7.93 [-9.29, -6.43] | -8.21 [-10.5, -6.64] | -9.29 [-10.6, -7.50] | -8.14 [-9.36, -6.64] | -7.29 [-9.07, -5.21] | -7.50 [-8.43, -6.07] | -6.50 [-7.86, -4.43] | -6.93 [-9.00, -5.14] |
|  |  | **Qch** | **No Cbr** |  |  |  |  |  |  | -1.50 [-3.43, 1.29] | -7.71 [-9.57, -6.07] | -8.00 [-10.4, -6.50] | -9.07 [-10.7, -7.28] | -7.93 [-9.71, -6.43] | -7.07 [-9.21, -5.21] | -7.29 [-8.93, -6.07] | -6.29 [-8.14, -4.29] | -6.71 [-8.93, -5.14] |
|  |  |  | **Cbr** |  |  |  |  |  |  |  | -6.21 [-8.86, -4.43] | -6.50 [-9.86, -4.79] | -7.57 [-10.3, -5.64] | -6.43 [-9.00, -4.71] | -5.57 [-8.57, -3.57] | -5.79 [-8.22, -3.93] | -4.79 [-7.43, -2.64] | -5.21 [-8.36, -3.36] |
| **Dct** | **No Rnt** | **No Qch** | **No Cbr** |  |  |  |  |  |  |  |  | -0.29 [-2.50, 1.22] | -1.36 [-2.57, 0.43] | -0.21 [-1.43, 1.29] | 0.64 [-1.14, 2.71] | 0.43 [-0.57, 1.50] | 1.43 [0.14, 3.36] | 1.00 [-1.00, 2.57] |
|  |  |  | **Cbr** |  |  |  |  |  |  |  |  |  | -1.07 [-2.36, 1.43] | 0.07 [-1.36, 2.36] | 0.93 [-0.86, 3.43] | 0.71 [-0.43, 2.71] | 1.71 [0.29, 4.29] | 1.29 [-0.64, 3.57] |
|  |  | **Qch** | **No Cbr** |  |  |  |  |  |  |  |  |  |  | 1.14 [-0.50, 2.43] | 2.00 [-0.14, 3.86] | 1.79 [0.21, 2.86] | 2.79 [1.00, 4.79] | 2.36 [0.14, 4.00] |
|  |  |  | **Cbr** |  |  |  |  |  |  |  |  |  |  |  | 0.86 [-1.00, 2.71] | 0.64 [-0.50, 1.79] | 1.64 [0.29, 3.50] | 1.21 [-0.93, 2.86] |
|  | **Rnt** | **No Qch** | **No Cbr** |  |  |  |  |  |  |  |  |  |  |  |  | -0.21 [-2.00, 1.43] | 0.79 [-1.00, 3.07] | 0.36 [-1.93, 2.36] |
|  |  |  | **Cbr** |  |  |  |  |  |  |  |  |  |  |  |  |  | 1.00 [-0.07, 2.86] | 0.57 [-1.43, 2.00] |
|  |  | **Qch** | **No Cbr** |  |  |  |  |  |  |  |  |  |  |  |  |  |  | -0.43 [-2.79, 1.07] |
|  |  |  | **Cbr** |  |  |  |  |  |  |  |  |  |  |  |  |  |  |  |

*Table S21:* *Difference of median with 95% CI for the “Max. 7-day Incidence” for all NPI configurations. A negative difference refers to an improvement from configurations in the columns with respect to the row, whereas a positive CI marks an improvement of the row configuration over the column. There is a statistical significant reduction in the “Max. 7-day Incidence” using DCT with any additional configuration (green). Without DCT, CBR alone has a statistically significant reduction effect (green and amber). DCT + RNT + QCH does significantly worse than DCT with QCH or CBR (amber). DCT + QCH does significantly better than DCT + RNT + any of the other NPIs (amber).*

## Max. 7-day Hosp. Incidence

| **Max. 7-day Hosp. Incidence** | | | | | | | | | | | | | | | | | | |
| --- | --- | --- | --- | --- | --- | --- | --- | --- | --- | --- | --- | --- | --- | --- | --- | --- | --- | --- |
|  | | | **DCT** | **No Dct** | | | | | | | **Dct** | | | | | | | |
|  |  |  | **RNT** |  |  |  | **Rnt** | | | | **No Rnt** | | | | **Rnt** | | | |
|  |  |  | **QCH** |  | **Qch** | | **No Qch** | | **Qch** | | **No Qch** | | **Qch** | | **No Qch** | | **Qch** | |
|  |  |  | **CBR** | **Cbr** | **No Cbr** | **Cbr** | **No Cbr** | **Cbr** | **No Cbr** | **Cbr** | **No Cbr** | **Cbr** | **No Cbr** | **Cbr** | **No Cbr** | **Cbr** | **No Cbr** | **Cbr** |
| **DCT** | **RNT** | **QCH** | **CBR** |  |  |  |  |  |  |  |  |  |  |  |  |  |  |  |
| **No Dct** | **No Rnt** | **No Qch** | **No Cbr** | -0.71 [-1.29, -0.21] | -0.14 [-0.64, 0.29] | -0.50 [-1.00, 0.14] | 0.21 [-0.43, 0.71] | 0.14 [-0.43, 0.71] | 0.00 [-0.57, 0.57] | -0.29 [-0.71, 0.43] | -1.57 [-2.07, -1.00] | -1.79 [-2.29, -1.21] | -1.86 [-2.36, -1.36] | -1.71 [-2.21, -1.21] | -1.50 [-2.00, -1.00] | -1.29 [-1.71, -0.86] | -1.21 [-1.79, -0.64] | -1.29 [-1.79, -0.86] |
|  |  |  | **Cbr** |  | 0.57 [0.00, 1.00] | 0.21 [-0.29, 0.86] | 0.93 [0.36, 1.36] | 0.86 [0.36, 1.43] | 0.71 [0.14, 1.29] | 0.43 [0.00, 1.14] | -0.86 [-1.43, -0.43] | -1.07 [-1.57, -0.50] | -1.14 [-1.71, -0.64] | -1.00 [-1.57, -0.57] | -0.79 [-1.29, -0.29] | -0.57 [-1.07, -0.07] | -0.50 [-1.07, 0.07] | -0.57 [-1.14, -0.00] |
|  |  | **Qch** | **No Cbr** |  |  | -0.36 [-0.79, 0.29] | 0.36 [-0.14, 0.71] | 0.29 [-0.14, 0.86] | 0.14 [-0.29, 0.71] | -0.14 [-0.43, 0.43] | -1.43 [-1.79, -1.00] | -1.64 [-2.00, -1.14] | -1.71 [-2.14, -1.29] | -1.57 [-2.00, -1.14] | -1.36 [-1.71, -0.86] | -1.14 [-1.43, -0.71] | -1.07 [-1.50, -0.57] | -1.14 [-1.57, -0.71] |
|  |  |  | **Cbr** |  |  |  | 0.71 [0.07, 1.14] | 0.64 [0.00, 1.14] | 0.50 [-0.14, 1.00] | 0.21 [-0.29, 0.86] | -1.07 [-1.64, -0.57] | -1.29 [-1.86, -0.71] | -1.36 [-1.93, -0.93] | -1.21 [-1.86, -0.79] | -1.00 [-1.57, -0.50] | -0.79 [-1.29, -0.36] | -0.71 [-1.29, -0.21] | -0.79 [-1.43, -0.36] |
|  | **Rnt** | **No Qch** | **No Cbr** |  |  |  |  | -0.07 [-0.57, 0.57] | -0.21 [-0.71, 0.43] | -0.50 [-0.86, 0.29] | -1.79 [-2.14, -1.14] | -2.00 [-2.36, -1.29] | -2.07 [-2.43, -1.57] | -1.93 [-2.36, -1.29] | -1.71 [-2.07, -1.14] | -1.50 [-1.79, -1.00] | -1.43 [-1.86, -0.71] | -1.50 [-1.93, -0.93] |
|  |  |  | **Cbr** |  |  |  |  |  | -0.14 [-0.71, 0.43] | -0.43 [-0.86, 0.21] | -1.71 [-2.21, -1.14] | -1.93 [-2.43, -1.36] | -2.00 [-2.50, -1.50] | -1.86 [-2.43, -1.36] | -1.64 [-2.14, -1.14] | -1.43 [-1.86, -1.00] | -1.36 [-1.93, -0.79] | -1.43 [-2.00, -1.00] |
|  |  | **Qch** | **No Cbr** |  |  |  |  |  |  | -0.29 [-0.71, 0.36] | -1.57 [-2.07, -1.07] | -1.79 [-2.29, -1.21] | -1.86 [-2.36, -1.29] | -1.71 [-2.29, -1.21] | -1.50 [-2.00, -1.00] | -1.29 [-1.71, -0.79] | -1.21 [-1.71, -0.57] | -1.29 [-1.86, -0.71] |
|  |  |  | **Cbr** |  |  |  |  |  |  |  | -1.29 [-1.86, -1.00] | -1.50 [-2.07, -1.14] | -1.57 [-2.14, -1.21] | -1.43 [-2.00, -1.07] | -1.21 [-1.86, -0.79] | -1.00 [-1.50, -0.71] | -0.93 [-1.57, -0.57] | -1.00 [-1.71, -0.64] |
| **Dct** | **No Rnt** | **No Qch** | **No Cbr** |  |  |  |  |  |  |  |  | -0.21 [-0.57, 0.29] | -0.29 [-0.71, 0.14] | -0.14 [-0.57, 0.29] | 0.07 [-0.29, 0.50] | 0.29 [-0.00, 0.64] | 0.36 [-0.14, 0.86] | 0.29 [-0.21, 0.71] |
|  |  |  | **Cbr** |  |  |  |  |  |  |  |  |  | -0.07 [-0.57, 0.36] | 0.07 [-0.43, 0.50] | 0.29 [-0.21, 0.71] | 0.50 [0.07, 0.86] | 0.57 [0.00, 1.07] | 0.50 [0.00, 0.86] |
|  |  | **Qch** | **No Cbr** |  |  |  |  |  |  |  |  |  |  | 0.14 [-0.29, 0.57] | 0.36 [-0.00, 0.79] | 0.57 [0.21, 0.93] | 0.64 [0.14, 1.14] | 0.57 [0.14, 1.00] |
|  |  |  | **Cbr** |  |  |  |  |  |  |  |  |  |  |  | 0.21 [-0.21, 0.71] | 0.43 [0.07, 0.86] | 0.50 [0.00, 1.00] | 0.43 [-0.00, 0.86] |
|  | **Rnt** | **No Qch** | **No Cbr** |  |  |  |  |  |  |  |  |  |  |  |  | 0.21 [-0.21, 0.57] | 0.29 [-0.21, 0.79] | 0.21 [-0.29, 0.64] |
|  |  |  | **Cbr** |  |  |  |  |  |  |  |  |  |  |  |  |  | 0.07 [-0.36, 0.50] | 0.00 [-0.43, 0.36] |
|  |  | **Qch** | **No Cbr** |  |  |  |  |  |  |  |  |  |  |  |  |  |  | -0.07 [-0.64, 0.43] |
|  |  |  | **Cbr** |  |  |  |  |  |  |  |  |  |  |  |  |  |  |  |

*Table S2*2 *Difference of median with 95% CI for the “Max. 7-day Hosp. Incidence” for all NPI configurations. A negative difference refers to an improvement from configurations in the columns with respect to the row, whereas a positive CI marks an improvement of the row configuration over the column. There is a statistical significant reduction in the “Max. 7-day Hosp. Incidence” using DCT with any additional configuration (green). Without DCT, CBR alone has a statistically significant reduction effect (green and amber). QCH alone statistically significantly improves over RNT alone. DCT + RNT + QCH does significantly worse than DCT with QCH or CBR (amber). DCT + QCH does significantly better than DCT + RNT + any of the other NPIs (amber).*

## Wave Duration

| **Wave Duration** | | | | | | | | | | | | | | | | | | |
| --- | --- | --- | --- | --- | --- | --- | --- | --- | --- | --- | --- | --- | --- | --- | --- | --- | --- | --- |
|  | | | **DCT** | **No Dct** | | | | | | | **Dct** | | | | | | | |
|  |  |  | **RNT** |  |  |  | **Rnt** | | | | **No Rnt** | | | | **Rnt** | | | |
|  |  |  | **QCH** |  | **Qch** | | **No Qch** | | **Qch** | | **No Qch** | | **Qch** | | **No Qch** | | **Qch** | |
|  |  |  | **CBR** | **Cbr** | **No Cbr** | **Cbr** | **No Cbr** | **Cbr** | **No Cbr** | **Cbr** | **No Cbr** | **Cbr** | **No Cbr** | **Cbr** | **No Cbr** | **Cbr** | **No Cbr** | **Cbr** |
| **DCT** | **RNT** | **QCH** | **CBR** |  |  |  |  |  |  |  |  |  |  |  |  |  |  |  |
| **No Dct** | **No Rnt** | **No Qch** | **No Cbr** | 15.3 [-1.01, 29.3] | -7.44 [-17.9, 7.74] | 12.6 [-0.89, 22.7] | 1.95 [-8.09, 11.5] | 5.55 [-6.35, 13.1] | 10.7 [3.07, 21.5] | 11.2 [1.15, 21.4] | 1.94 [-11.9, 20.3] | -14.2 [-27.8, 1.77] | -15.4 [-27.3, 9.17] | 0.45 [-10.6, 20.3] | 5.37 [-9.46, 17.3] | 14.7 [0.09, 26.8] | 10.4 [-2.80, 22.2] | 19.0 [3.07, 29.7] |
|  |  |  | **Cbr** |  | -22.7 [-35.3, -2.60] | -2.69 [-20.8, 13.3] | -13.3 [-26.8, 0.94] | -9.74 [-26.7, 3.03] | -4.61 [-18.5, 9.84] | -4.14 [-17.8, 10.5] | -13.3 [-29.2, 7.90] | -29.5 [-48.5, -9.70] | -30.6 [-46.7, -2.55] | -14.8 [-28.1, 10.4] | -9.92 [-27.9, 7.48] | -0.58 [-18.8, 15.9] | -4.93 [-22.3, 11.2] | 3.70 [-15.2, 19.0] |
|  |  | **Qch** | **No Cbr** |  |  | 20.0 [1.19, 31.3] | 9.39 [-6.03, 18.6] | 13.0 [-4.30, 20.1] | 18.1 [2.62, 28.2] | 18.6 [1.16, 28.3] | 9.38 [-7.19, 25.9] | -6.73 [-24.9, 7.29] | -7.92 [-23.7, 16.4] | 7.89 [-8.92, 27.0] | 12.8 [-6.24, 23.2] | 22.2 [4.82, 34.2] | 17.8 [-1.29, 29.0] | 26.4 [7.53, 35.7] |
|  |  |  | **Cbr** |  |  |  | -10.6 [-19.7, 2.59] | -7.05 [-19.4, 5.17] | -1.92 [-10.6, 12.7] | -1.45 [-11.2, 12.0] | -10.7 [-22.4, 7.91] | -26.8 [-40.6, -11.4] | -28.0 [-40.1, -0.79] | -12.1 [-23.1, 12.4] | -7.23 [-21.5, 7.28] | 2.11 [-13.3, 18.3] | -2.23 [-16.4, 11.6] | 6.39 [-8.35, 19.4] |
|  | **Rnt** | **No Qch** | **No Cbr** |  |  |  |  | 3.60 [-8.18, 10.0] | 8.73 [0.63, 18.8] | 9.20 [-1.09, 18.2] | -0.01 [-11.2, 17.7] | -16.1 [-30.1, -2.64] | -17.3 [-27.2, 3.11] | -1.50 [-12.1, 17.2] | 3.42 [-10.3, 13.6] | 12.8 [-1.47, 24.4] | 8.41 [-4.67, 20.2] | 17.0 [2.98, 26.7] |
|  |  |  | **Cbr** |  |  |  |  |  | 5.13 [-0.77, 17.8] | 5.60 [-2.12, 17.4] | -3.61 [-13.4, 14.9] | -19.7 [-32.5, -2.25] | -20.9 [-31.1, 5.11] | -5.10 [-14.0, 16.0] | -0.18 [-12.0, 13.0] | 9.16 [-1.35, 22.9] | 4.81 [-7.10, 17.7] | 13.4 [0.48, 26.3] |
|  |  | **Qch** | **No Cbr** |  |  |  |  |  |  | 0.47 [-10.7, 7.78] | -8.74 [-22.0, 7.70] | -24.9 [-39.1, -11.6] | -26.0 [-38.8, -4.17] | -10.2 [-21.9, 8.34] | -5.31 [-19.2, 5.23] | 4.03 [-9.78, 14.9] | -0.32 [-15.0, 11.1] | 8.31 [-7.87, 17.5] |
|  |  |  | **Cbr** |  |  |  |  |  |  |  | -9.21 [-20.0, 8.83] | -25.3 [-38.1, -8.52] | -26.5 [-38.0, -0.10] | -10.7 [-20.4, 9.31] | -5.78 [-19.1, 6.49] | 3.56 [-9.91, 17.0] | -0.79 [-15.3, 12.4] | 7.84 [-7.32, 18.5] |
| **Dct** | **No Rnt** | **No Qch** | **No Cbr** |  |  |  |  |  |  |  |  | -16.1 [-35.5, 2.49] | -17.3 [-35.1, 10.0] | -1.49 [-18.3, 19.7] | 3.43 [-15.3, 18.3] | 12.8 [-6.96, 25.1] | 8.42 [-10.3, 21.8] | 17.0 [-2.77, 29.6] |
|  |  |  | **Cbr** |  |  |  |  |  |  |  |  |  | -1.19 [-15.4, 23.8] | 14.6 [-0.76, 36.8] | 19.5 [2.82, 36.4] | 28.9 [10.7, 43.5] | 24.5 [7.27, 41.2] | 33.2 [15.3, 46.5] |
|  |  | **Qch** | **No Cbr** |  |  |  |  |  |  |  |  |  |  | 15.8 [-10.6, 35.5] | 20.7 [-4.08, 34.2] | 30.1 [1.65, 43.1] | 25.7 [-0.63, 38.9] | 34.3 [5.79, 45.9] |
|  |  |  | **Cbr** |  |  |  |  |  |  |  |  |  |  |  | 4.92 [-15.9, 16.6] | 14.3 [-7.15, 26.4] | 9.91 [-13.2, 20.9] | 18.5 [-5.32, 28.7] |
|  | **Rnt** | **No Qch** | **No Cbr** |  |  |  |  |  |  |  |  |  |  |  |  | 9.34 [-5.00, 24.9] | 4.99 [-9.43, 18.4] | 13.6 [-2.69, 26.7] |
|  |  |  | **Cbr** |  |  |  |  |  |  |  |  |  |  |  |  |  | -4.35 [-19.6, 10.0] | 4.28 [-12.4, 18.4] |
|  |  | **Qch** | **No Cbr** |  |  |  |  |  |  |  |  |  |  |  |  |  |  | 8.62 [-7.70, 22.5] |
|  |  |  | **Cbr** |  |  |  |  |  |  |  |  |  |  |  |  |  |  |  |

*Table S23:* *Difference of median with 95% CI for the “Wave Duration” for all NPI configurations. A negative difference refers to an improvement from configurations in the columns with respect to the row and is marked green if the difference is significant. Whereas a positive CI marks an improvement of the row configuration over the column and is highlighted amber. There are several configurations with statistical significant difference of medians, the possible reductions is between ~19% - 35%. We observe that DCT + QCH or DCT + CBR.*

## Days in Quarantine

| **Days in Quarantine** | | | | | | | | | | | | | | | | | | |
| --- | --- | --- | --- | --- | --- | --- | --- | --- | --- | --- | --- | --- | --- | --- | --- | --- | --- | --- |
|  | | | **DCT** | **No Dct** | | | | | | | **Dct** | | | | | | | |
|  |  |  | **RNT** |  |  |  | **Rnt** | | | | **No Rnt** | | | | **Rnt** | | | |
|  |  |  | **QCH** |  | **Qch** | | **No Qch** | | **Qch** | | **No Qch** | | **Qch** | | **No Qch** | | **Qch** | |
|  |  |  | **CBR** | **Cbr** | **No Cbr** | **Cbr** | **No Cbr** | **Cbr** | **No Cbr** | **Cbr** | **No Cbr** | **Cbr** | **No Cbr** | **Cbr** | **No Cbr** | **Cbr** | **No Cbr** | **Cbr** |
| **DCT** | **RNT** | **QCH** | **CBR** |  |  |  |  |  |  |  |  |  |  |  |  |  |  |  |
| **No Dct** | **No Rnt** | **No Qch** | **No Cbr** | -0.25 [-1.98, 1.34] | 0.32 [-1.29, 1.62] | -0.38 [-2.50, 1.25] | -19.3 [-20.7, -17.1] | -19.7 [-21.2, -18.3] | -20.2 [-21.4, -18.5] | -19.7 [-21.2, -17.7] | -0.88 [-11.5, 2.46] | 0.33 [-1.25, 2.78] | -0.37 [-10.2, 1.48] | -1.60 [-10.7, 3.38] | -17.8 [-19.9, -14.8] | -18.8 [-20.3, -15.5] | -17.5 [-20.1, -15.4] | -18.7 [-20.1, -16.8] |
|  |  |  | **Cbr** |  | 0.56 [-1.26, 2.41] | -0.13 [-2.38, 1.79] | -19.0 [-20.8, -16.8] | -19.5 [-21.3, -17.8] | -20.0 [-21.4, -18.0] | -19.5 [-21.2, -17.3] | -0.63 [-11.3, 2.92] | 0.57 [-1.32, 3.19] | -0.12 [-9.66, 2.16] | -1.35 [-11.1, 4.05] | -17.5 [-20.0, -14.4] | -18.5 [-20.3, -14.9] | -17.3 [-20.2, -14.9] | -18.5 [-20.1, -16.4] |
|  |  | **Qch** | **No Cbr** |  |  | -0.70 [-2.63, 1.35] | -19.6 [-21.0, -17.5] | -20.0 [-21.5, -18.4] | -20.5 [-21.6, -18.9] | -20.0 [-21.7, -17.8] | -1.19 [-11.6, 2.20] | 0.01 [-1.59, 2.83] | -0.69 [-10.6, 1.29] | -1.92 [-11.2, 3.73] | -18.1 [-20.2, -15.1] | -19.1 [-20.6, -15.9] | -17.9 [-20.4, -15.4] | -19.0 [-20.5, -16.8] |
|  |  |  | **Cbr** |  |  |  | -18.9 [-20.6, -16.5] | -19.3 [-21.1, -17.2] | -19.8 [-21.3, -17.8] | -19.3 [-21.1, -17.0] | -0.50 [-10.9, 3.00] | 0.71 [-1.10, 3.52] | 0.01 [-10.1, 2.45] | -1.22 [-10.6, 3.81] | -17.4 [-19.9, -13.9] | -18.4 [-20.2, -14.9] | -17.2 [-19.8, -14.3] | -18.3 [-20.1, -16.0] |
|  | **Rnt** | **No Qch** | **No Cbr** |  |  |  |  | -0.45 [-2.73, 1.19] | -0.96 [-2.91, 0.71] | -0.45 [-2.64, 1.56] | 18.4 [7.30, 21.7] | 19.6 [17.3, 22.0] | 18.9 [11.2, 20.8] | 17.7 [8.30, 22.9] | 1.48 [-0.89, 4.48] | 0.51 [-1.63, 3.70] | 1.73 [-1.15, 4.17] | 0.54 [-1.60, 2.60] |
|  |  |  | **Cbr** |  |  |  |  |  | -0.51 [-1.59, 1.28] | -0.00 [-1.37, 2.26] | 18.8 [8.68, 22.2] | 20.0 [18.5, 22.6] | 19.3 [9.51, 21.5] | 18.1 [9.03, 23.9] | 1.93 [-0.13, 4.96] | 0.96 [-0.39, 4.28] | 2.18 [-0.36, 4.49] | 0.99 [-0.38, 3.35] |
|  |  | **Qch** | **No Cbr** |  |  |  |  |  |  | 0.51 [-1.14, 2.32] | 19.3 [8.71, 22.5] | 20.6 [18.8, 23.0] | 19.9 [10.1, 21.7] | 18.6 [9.44, 23.7] | 2.44 [0.28, 5.27] | 1.47 [-0.18, 4.49] | 2.69 [-0.07, 4.76] | 1.50 [-0.12, 3.51] |
|  |  |  | **Cbr** |  |  |  |  |  |  |  | 18.8 [7.73, 22.1] | 20.0 [18.2, 22.8] | 19.3 [9.07, 21.5] | 18.1 [8.53, 23.2] | 1.93 [-0.40, 4.99] | 0.96 [-1.20, 4.34] | 2.18 [-0.98, 4.39] | 0.99 [-1.00, 3.20] |
| **Dct** | **No Rnt** | **No Qch** | **No Cbr** |  |  |  |  |  |  |  |  | 1.20 [-2.08, 12.1] | 0.51 [-7.63, 11.6] | -0.72 [-11.5, 12.7] | -16.9 [-20.8, -4.79] | -17.9 [-21.3, -5.63] | -16.7 [-20.9, -5.29] | -17.9 [-21.4, -6.76] |
|  |  |  | **Cbr** |  |  |  |  |  |  |  |  |  | -0.70 [-10.4, 1.28] | -1.93 [-11.7, 3.38] | -18.1 [-21.1, -15.1] | -19.1 [-21.4, -15.7] | -17.9 [-20.9, -15.7] | -19.1 [-21.4, -17.0] |
|  |  | **Qch** | **No Cbr** |  |  |  |  |  |  |  |  |  |  | -1.23 [-10.9, 9.09] | -17.4 [-19.9, -8.76] | -18.4 [-20.5, -8.83] | -17.2 [-20.2, -9.04] | -18.4 [-20.3, -8.53] |
|  |  |  | **Cbr** |  |  |  |  |  |  |  |  |  |  |  | -16.2 [-21.8, -5.80] | -17.2 [-23.1, -6.13] | -15.9 [-22.1, -6.13] | -17.1 [-22.3, -7.79] |
|  | **Rnt** | **No Qch** | **No Cbr** |  |  |  |  |  |  |  |  |  |  |  |  | -0.97 [-3.95, 3.08] | 0.25 [-3.84, 3.01] | -0.94 [-3.98, 1.66] |
|  |  |  | **Cbr** |  |  |  |  |  |  |  |  |  |  |  |  |  | 1.22 [-3.20, 3.49] | 0.03 [-3.11, 2.23] |
|  |  | **Qch** | **No Cbr** |  |  |  |  |  |  |  |  |  |  |  |  |  |  | -1.19 [-3.32, 1.88] |
|  |  |  | **Cbr** |  |  |  |  |  |  |  |  |  |  |  |  |  |  |  |

*Table S24:* *Difference of median with 95% CI for the “Days in Quarantine” for all NPI configurations. A negative difference refers to an improvement from configurations in the columns with respect to the row and is marked green if the difference is significant. Whereas a positive CI marks an improvement of the row configuration over the column and is highlighted amber. RNT has a direct impact in the reduction of “Days in Quarantine”. RNT in addition to any other NPI has no statistically significant benefit.*

## Generation Interval

| **Generation Interval** | | | | | | | | | | | | | | | | | | |
| --- | --- | --- | --- | --- | --- | --- | --- | --- | --- | --- | --- | --- | --- | --- | --- | --- | --- | --- |
|  | | | **DCT** | **No Dct** | | | | | | | **Dct** | | | | | | | |
|  |  |  | **RNT** |  |  |  | **Rnt** | | | | **No Rnt** | | | | **Rnt** | | | |
|  |  |  | **QCH** |  | **Qch** | | **No Qch** | | **Qch** | | **No Qch** | | **Qch** | | **No Qch** | | **Qch** | |
|  |  |  | **CBR** | **Cbr** | **No Cbr** | **Cbr** | **No Cbr** | **Cbr** | **No Cbr** | **Cbr** | **No Cbr** | **Cbr** | **No Cbr** | **Cbr** | **No Cbr** | **Cbr** | **No Cbr** | **Cbr** |
| **DCT** | **RNT** | **QCH** | **CBR** |  |  |  |  |  |  |  |  |  |  |  |  |  |  |  |
| **No Dct** | **No Rnt** | **No Qch** | **No Cbr** | 0.15 [-1.58, 1.01] | 0.75 [-0.94, 2.00] | 0.01 [-1.51, 1.69] | -0.04 [-1.28, 0.85] | -0.74 [-1.86, 0.42] | -0.03 [-1.51, 1.32] | -0.21 [-2.09, 1.13] | -0.82 [-2.15, 0.34] | -0.90 [-2.33, 0.51] | -0.47 [-2.06, 1.42] | -0.77 [-2.09, 0.70] | -0.10 [-1.88, 0.74] | -0.18 [-1.57, 0.73] | -0.66 [-1.96, 0.62] | 0.39 [-0.97, 1.63] |
|  |  |  | **Cbr** |  | 0.61 [-0.47, 1.87] | -0.13 [-1.08, 1.56] | -0.18 [-0.97, 0.77] | -0.89 [-1.46, 0.28] | -0.18 [-1.16, 1.23] | -0.35 [-1.75, 1.11] | -0.97 [-1.73, 0.29] | -1.05 [-1.98, 0.47] | -0.62 [-1.72, 1.54] | -0.92 [-1.68, 0.61] | -0.25 [-1.31, 0.66] | -0.32 [-1.11, 0.62] | -0.81 [-1.44, 0.51] | 0.24 [-0.45, 1.61] |
|  |  | **Qch** | **No Cbr** |  |  | -0.74 [-1.90, 1.07] | -0.79 [-1.84, 0.25] | -1.50 [-2.22, -0.18] | -0.79 [-1.86, 0.72] | -0.96 [-2.55, 0.62] | -1.58 [-2.54, -0.24] | -1.65 [-2.83, -0.11] | -1.23 [-2.57, 0.77] | -1.53 [-2.46, -0.09] | -0.85 [-2.21, 0.02] | -0.93 [-1.97, 0.02] | -1.41 [-2.33, 0.08] | -0.37 [-1.33, 1.08] |
|  |  |  | **Cbr** |  |  |  | -0.05 [-1.74, 0.91] | -0.76 [-2.14, 0.32] | -0.05 [-1.74, 1.31] | -0.22 [-2.21, 1.13] | -0.84 [-2.40, 0.30] | -0.91 [-2.50, 0.50] | -0.49 [-2.32, 1.54] | -0.79 [-2.41, 0.61] | -0.11 [-2.00, 0.75] | -0.19 [-1.85, 0.74] | -0.67 [-2.16, 0.58] | 0.37 [-1.04, 1.73] |
|  | **Rnt** | **No Qch** | **No Cbr** |  |  |  |  | -0.71 [-1.11, 0.34] | 0.00 [-1.07, 1.22] | -0.17 [-1.57, 0.97] | -0.79 [-1.48, 0.20] | -0.86 [-1.81, 0.39] | -0.44 [-1.51, 1.36] | -0.74 [-1.39, 0.36] | -0.06 [-1.20, 0.48] | -0.14 [-0.99, 0.49] | -0.62 [-1.19, 0.53] | 0.42 [-0.22, 1.60] |
|  |  |  | **Cbr** |  |  |  |  |  | 0.71 [-0.65, 1.80] | 0.54 [-1.03, 1.63] | -0.08 [-1.13, 0.76] | -0.15 [-1.25, 0.89] | 0.27 [-1.14, 2.02] | -0.03 [-1.10, 1.01] | 0.64 [-0.70, 0.94] | 0.57 [-0.56, 0.90] | 0.09 [-1.02, 1.02] | 1.13 [0.05, 2.08] |
|  |  | **Qch** | **No Cbr** |  |  |  |  |  |  | -0.17 [-1.83, 1.33] | -0.79 [-1.98, 0.31] | -0.86 [-2.09, 0.44] | -0.44 [-2.05, 1.68] | -0.74 [-2.03, 0.89] | -0.07 [-1.74, 0.72] | -0.14 [-1.39, 0.80] | -0.62 [-1.86, 0.82] | 0.42 [-0.78, 1.76] |
|  |  |  | **Cbr** |  |  |  |  |  |  |  | -0.62 [-1.90, 0.87] | -0.69 [-1.94, 1.04] | -0.27 [-1.80, 1.90] | -0.57 [-1.85, 1.26] | 0.11 [-1.42, 1.44] | 0.03 [-1.19, 1.43] | -0.45 [-1.69, 1.34] | 0.59 [-0.68, 2.42] |
| **Dct** | **No Rnt** | **No Qch** | **No Cbr** |  |  |  |  |  |  |  |  | -0.07 [-1.14, 1.11] | 0.35 [-0.88, 2.28] | 0.05 [-0.95, 1.36] | 0.72 [-0.57, 1.46] | 0.65 [-0.37, 1.39] | 0.16 [-0.73, 1.39] | 1.21 [0.17, 2.35] |
|  |  |  | **Cbr** |  |  |  |  |  |  |  |  |  | 0.43 [-1.00, 2.17] | 0.12 [-1.06, 1.59] | 0.80 [-0.88, 1.59] | 0.72 [-0.60, 1.60] | 0.24 [-0.90, 1.38] | 1.28 [0.08, 2.63] |
|  |  | **Qch** | **No Cbr** |  |  |  |  |  |  |  |  |  |  | -0.30 [-2.29, 1.37] | 0.37 [-1.84, 1.36] | 0.30 [-1.68, 1.35] | -0.19 [-2.13, 1.21] | 0.86 [-0.99, 2.40] |
|  |  |  | **Cbr** |  |  |  |  |  |  |  |  |  |  |  | 0.67 [-0.94, 1.19] | 0.60 [-0.71, 1.22] | 0.12 [-1.03, 1.25] | 1.16 [-0.03, 2.35] |
|  | **Rnt** | **No Qch** | **No Cbr** |  |  |  |  |  |  |  |  |  |  |  |  | -0.08 [-0.73, 0.93] | -0.56 [-1.01, 0.92] | 0.49 [-0.04, 1.98] |
|  |  |  | **Cbr** |  |  |  |  |  |  |  |  |  |  |  |  |  | -0.48 [-1.01, 0.76] | 0.56 [-0.01, 1.71] |
|  |  | **Qch** | **No Cbr** |  |  |  |  |  |  |  |  |  |  |  |  |  |  | 1.05 [0.03, 2.07] |
|  |  |  | **Cbr** |  |  |  |  |  |  |  |  |  |  |  |  |  |  |  |

*Table S25:* *Difference of median with 95% CI for the “Generation Interval” for all NPI configurations. A negative difference refers to an improvement from configurations in the columns with respect to the row and is marked green if the difference is significant. Whereas a positive CI marks an improvement of the row configuration over the column and is highlighted amber. There is no NPI that controls the “Generation Interval”. Some combinations might perform better than others, but there is no NPI that outperforms all other combinations.*

## Serial Interval

| **Serial Interval** | | | | | | | | | | | | | | | | | | |
| --- | --- | --- | --- | --- | --- | --- | --- | --- | --- | --- | --- | --- | --- | --- | --- | --- | --- | --- |
|  | | | **DCT** | **No Dct** | | | | | | | **Dct** | | | | | | | |
|  |  |  | **RNT** |  |  |  | **Rnt** | | | | **No Rnt** | | | | **Rnt** | | | |
|  |  |  | **QCH** |  | **Qch** | | **No Qch** | | **Qch** | | **No Qch** | | **Qch** | | **No Qch** | | **Qch** | |
|  |  |  | **CBR** | **Cbr** | **No Cbr** | **Cbr** | **No Cbr** | **Cbr** | **No Cbr** | **Cbr** | **No Cbr** | **Cbr** | **No Cbr** | **Cbr** | **No Cbr** | **Cbr** | **No Cbr** | **Cbr** |
| **DCT** | **RNT** | **QCH** | **CBR** |  |  |  |  |  |  |  |  |  |  |  |  |  |  |  |
| **No Dct** | **No Rnt** | **No Qch** | **No Cbr** | 0.27 [-1.41, 2.13] | -0.70 [-2.05, 0.74] | -2.26 [-3.09, -0.42] | -0.51 [-1.36, 1.20] | -0.98 [-1.94, 0.70] | -0.59 [-2.10, 1.03] | -0.43 [-1.91, 1.18] | -0.95 [-2.04, 1.22] | -1.03 [-2.17, 0.61] | -1.07 [-2.46, 0.81] | -0.24 [-1.43, 1.52] | -0.59 [-1.71, 1.16] | -1.14 [-2.45, 0.38] | 0.30 [-1.24, 1.94] | -1.25 [-2.04, 0.46] |
|  |  |  | **Cbr** |  | -0.97 [-2.66, 0.70] | -2.53 [-3.66, -0.44] | -0.77 [-1.93, 1.03] | -1.24 [-2.41, 0.59] | -0.86 [-2.35, 1.01] | -0.69 [-2.27, 1.19] | -1.21 [-2.45, 1.43] | -1.30 [-2.74, 0.49] | -1.33 [-2.99, 0.91] | -0.51 [-1.99, 1.32] | -0.86 [-2.27, 1.02] | -1.40 [-2.88, 0.45] | 0.03 [-1.83, 1.57] | -1.52 [-2.64, 0.40] |
|  |  | **Qch** | **No Cbr** |  |  | -1.56 [-2.31, 0.32] | 0.19 [-0.49, 1.49] | -0.28 [-1.00, 1.31] | 0.11 [-0.98, 1.72] | 0.27 [-1.00, 1.75] | -0.24 [-1.38, 1.93] | -0.33 [-1.32, 1.19] | -0.36 [-1.71, 1.68] | 0.46 [-0.75, 2.00] | 0.11 [-0.95, 1.59] | -0.43 [-1.68, 0.85] | 1.00 [-0.49, 2.27] | -0.55 [-1.27, 1.05] |
|  |  |  | **Cbr** |  |  |  | 1.76 [0.15, 2.88] | 1.29 [-0.51, 2.69] | 1.67 [-0.14, 2.79] | 1.84 [0.16, 3.07] | 1.32 [-0.28, 3.34] | 1.23 [-0.41, 2.17] | 1.20 [-0.95, 2.69] | 2.02 [0.25, 3.02] | 1.67 [-0.10, 2.61] | 1.13 [-0.75, 2.02] | 2.56 [0.45, 3.44] | 1.01 [-0.60, 2.12] |
|  | **Rnt** | **No Qch** | **No Cbr** |  |  |  |  | -0.47 [-1.68, 0.83] | -0.08 [-1.74, 1.05] | 0.08 [-1.72, 1.22] | -0.44 [-1.59, 1.48] | -0.52 [-1.64, 0.42] | -0.56 [-1.92, 0.90] | 0.26 [-1.17, 1.30] | -0.09 [-1.33, 0.97] | -0.63 [-2.04, 0.27] | 0.81 [-0.89, 1.60] | -0.74 [-1.72, 0.26] |
|  |  |  | **Cbr** |  |  |  |  |  | 0.39 [-1.23, 1.63] | 0.55 [-1.19, 1.74] | 0.03 [-1.45, 2.01] | -0.05 [-1.56, 1.00] | -0.09 [-1.80, 1.40] | 0.73 [-0.76, 1.96] | 0.39 [-1.00, 1.50] | -0.16 [-1.93, 0.85] | 1.28 [-0.62, 2.22] | -0.27 [-1.40, 0.90] |
|  |  | **Qch** | **No Cbr** |  |  |  |  |  |  | 0.16 [-1.48, 1.71] | -0.36 [-1.53, 1.81] | -0.44 [-1.66, 1.15] | -0.48 [-2.05, 1.44] | 0.35 [-1.10, 2.07] | -0.00 [-1.36, 1.47] | -0.55 [-2.03, 0.75] | 0.89 [-0.97, 2.31] | -0.66 [-1.64, 0.81] |
|  |  |  | **Cbr** |  |  |  |  |  |  |  | -0.52 [-1.79, 1.84] | -0.60 [-1.92, 1.11] | -0.64 [-2.25, 1.16] | 0.18 [-1.24, 1.74] | -0.16 [-1.45, 1.50] | -0.71 [-2.12, 0.62] | 0.73 [-1.12, 2.02] | -0.82 [-1.99, 0.80] |
| **Dct** | **No Rnt** | **No Qch** | **No Cbr** |  |  |  |  |  |  |  |  | -0.09 [-2.14, 0.99] | -0.12 [-2.56, 1.26] | 0.70 [-1.58, 2.08] | 0.35 [-1.88, 1.56] | -0.19 [-2.49, 0.89] | 1.24 [-1.27, 2.19] | -0.31 [-2.22, 0.84] |
|  |  |  | **Cbr** |  |  |  |  |  |  |  |  |  | -0.03 [-1.64, 1.50] | 0.79 [-0.59, 2.23] | 0.44 [-0.82, 1.75] | -0.10 [-1.39, 0.93] | 1.33 [-0.35, 2.48] | -0.22 [-1.19, 1.03] |
|  |  | **Qch** | **No Cbr** |  |  |  |  |  |  |  |  |  |  | 0.82 [-1.02, 2.38] | 0.47 [-1.25, 2.13] | -0.07 [-1.76, 1.35] | 1.36 [-0.80, 2.75] | -0.19 [-1.63, 1.36] |
|  |  |  | **Cbr** |  |  |  |  |  |  |  |  |  |  |  | -0.35 [-1.70, 0.96] | -0.89 [-2.32, 0.34] | 0.54 [-1.20, 1.65] | -1.01 [-1.95, 0.40] |
|  | **Rnt** | **No Qch** | **No Cbr** |  |  |  |  |  |  |  |  |  |  |  |  | -0.54 [-1.88, 0.56] | 0.89 [-0.93, 1.96] | -0.66 [-1.58, 0.74] |
|  |  |  | **Cbr** |  |  |  |  |  |  |  |  |  |  |  |  |  | 1.43 [-0.20, 2.64] | -0.12 [-1.00, 1.46] |
|  |  | **Qch** | **No Cbr** |  |  |  |  |  |  |  |  |  |  |  |  |  |  | -1.55 [-2.39, 0.24] |
|  |  |  | **Cbr** |  |  |  |  |  |  |  |  |  |  |  |  |  |  |  |

*Table S27:* *Difference of median with 95% CI for the “Serial Interval” for all NPI configurations. A negative difference refers to an improvement from configurations in the columns with respect to the row and is marked green if the difference is significant. Whereas a positive CI marks an improvement of the row configuration over the column and is highlighted amber. There is no NPI that controls the “Serial Interval”. Some combinations might perform better than others, but there is no consistent NPI that outperforms all other combinations.*

# Standard Relative Error

We evaluated the standard relative error for the 50 runs of our optimal and realistic behaviours evaluation scenarios. The error was calculated as follows:

$\frac{\sqrt{\frac{s^{2}}{N}}}{\theta}\cdot100\%$ ,

where s is the variance of the pandemic characteristic estimator, $\theta$ is the estimator’s median, and N is the 50 runs per NPI combination.

## Optimal Behaviour

| **DCT** | **No Dct** | **Dct** | | | | | | | |
| --- | --- | --- | --- | --- | --- | --- | --- | --- | --- |
| **RNT** | **No Rnt** | **No Rnt** | | | | **Rnt** | | | |
| **QCH** | **No Qch** | **No Qch** | | **Qch** | | **No Qch** | | **Qch** | |
| **CBR** | **No Cbr** | **No Cbr** | **Cbr** | **No Cbr** | **Cbr** | **No Cbr** | **Cbr** | **No Cbr** | **Cbr** |
| **Total infected** | 3,49% | 9,49% | 11,94% | 8,02% | 10,53% | 12,42% | 10,55% | 15,22% | 14,84% |
| **7-day Hosp. Incidence** | 5,00% | 10,20% | 11,93% | 7,40% | 8,38% | 8,13% | 10,73% | 9,04% | 11,97% |
| **7-day Incidence** | 5,21% | 7,17% | 9,19% | 6,51% | 8,73% | 7,55% | 8,82% | 8,67% | 7,95% |
| **Wave Duration** | 3,91% | 6,28% | 5,15% | 4,41% | 3,89% | 6,58% | 6,14% | 5,27% | 6,88% |
| **Days in Quarantine** | 1,25% | 1,48% | 1,04% | 1,07% | 1,13% | 1,65% | 1,69% | 1,61% | 1,65% |
| **Generation Interval** | 0,78% | 2,26% | 2,37% | 2,87% | 1,98% | 2,37% | 1,88% | 1,76% | 1,90% |
| **Serial Interval** | 0,88% | 2,07% | 2,94% | 3,13% | 2,22% | 1,93% | 1,88% | 2,05% | 1,75% |

*Table S28: Standard relative error for optimal behaviour configuration scenarios. For most variable configurations, the error was below 5% of the respective metric. For other variables, the error has no practical implications; even the largest error, 15% of “Total infected”, corresponds to only 6 individuals, representing 0.6% of the total simulated population.*

## Realistic Behaviour

| **DCT** | **No Dct** | | | | | | | | **Dct** | | | | | | | |
| --- | --- | --- | --- | --- | --- | --- | --- | --- | --- | --- | --- | --- | --- | --- | --- | --- |
| **RNT** | **No Rnt** | | | | **Rnt** | | | | **No Rnt** | | | | **Rnt** | | | |
| **QCH** | **No Qch** | | **Qch** | | **No Qch** | | **Qch** | | **No Qch** | | **Qch** | | **No Qch** | | **Qch** | |
| **CBR** | **No Cbr** | **Cbr** | **No Cbr** | **Cbr** | **No Cbr** | **Cbr** | **No Cbr** | **Cbr** | **No Cbr** | **Cbr** | **No Cbr** | **Cbr** | **No Cbr** | **Cbr** | **No Cbr** | **Cbr** |
| **Total infected** | 4,12% | 5,90% | 4,97% | 4,41% | 2,29% | 3,56% | 3,03% | 4,27% | 5,82% | 7,77% | 7,08% | 5,76% | 6,20% | 5,93% | 5,74% | 5,94% |
| **7-day Hosp. Incidence** | 4,67% | 7,21% | 5,42% | 5,76% | 3,94% | 4,26% | 4,55% | 5,37% | 5,18% | 6,79% | 7,63% | 6,35% | 6,80% | 6,07% | 6,43% | 5,79% |
| **7-day Incidence** | 5,05% | 7,49% | 5,77% | 5,86% | 3,99% | 4,20% | 4,65% | 5,67% | 5,07% | 6,59% | 6,68% | 6,10% | 6,47% | 5,65% | 7,15% | 6,07% |
| **Wave Duration** | 4,37% | 2,99% | 4,78% | 4,61% | 3,52% | 4,44% | 3,10% | 3,73% | 5,60% | 6,78% | 5,83% | 4,98% | 5,66% | 5,36% | 4,61% | 4,93% |
| **Days in Quarantine** | 1,36% | 0,49% | 1,65% | 1,36% | 0,82% | 0,90% | 0,82% | 0,99% | 2,44% | 2,84% | 2,20% | 2,19% | 2,21% | 2,32% | 2,26% | 2,14% |
| **Generation Interval** | 0,73% | 0,60% | 1,06% | 0,64% | 0,60% | 0,72% | 0,65% | 0,78% | 1,11% | 1,04% | 1,06% | 0,92% | 1,37% | 1,07% | 1,32% | 0,90% |
| **Serial Interval** | 0,86% | 0,52% | 1,41% | 0,65% | 1,60% | 0,71% | 0,57% | 0,85% | 0,58% | 1,06% | 1,07% | 0,85% | 1,75% | 0,79% | 2,12% | 1,03% |

*Table S29: Standard relative error for realistic behaviour configuration scenarios. For most variable configurations, the error was below 5% of the respective metric. For other variables, the error has no practical implications; even the largest error, 8% of “Total infected” is about 25 people, representing 2.5% of the total simulated population.*

# Effect sizes

Since we are using medians to measure our central tendencies, we used Cohen's d compatible effect size [13] calculations using the following formulas.

$$d = \frac{\left| Median_{1}- Median_{2} \right|}{"Pooled MAD"}$$

$$"Pooled MAD" = \sqrt{\frac{((n₁ - 1) * MAD₁^{2} + (n₂ - 1) * MAD₂^{2})}{n₁ + n₂ - 2}}$$

$$MAD=median(\left| Xi-median(X) \right|)$$

Where $Xi$ is a sample in $X$, i.e., the set of 50 runs for a pandemic characteristic, $MAD_{1}$ and $MAD_{2}$are the median absolute deviation for run configurations 1 and 2, $n₁ = n₂ =50$ is the number of runs, and $Median_{1}$ and $Median_{2}$ are the respective configuration medians. We applied the following qualitative boundaries to the effect size interpretation: Small - d < 0.2, Medium - 0.2 < d < 0.8, Large d > 0.8.

**Total Infected**

### Optimal Behaviour

|  |  |  | **DCT** | **No Dct** | **Dct** | | | | | | | |
| --- | --- | --- | --- | --- | --- | --- | --- | --- | --- | --- | --- | --- |
|  |  |  | **RNT** | **No Rnt** | **No Rnt** | | | | **Rnt** | | | |
|  |  |  | **QCH** | **No Qch** | **No Qch** | | **Qch** | | **No Qch** | | **Qch** | |
|  |  |  | **CBR** | **No Cbr** | **No Cbr** | **Cbr** | **No Cbr** | **Cbr** | **No Cbr** | **Cbr** | **No Cbr** | **Cbr** |
| **DCT** | **RNT** | **QCH** | **CBR** |  |  |  |  |  |  |  |  |  |
| **No Dct** | **No Rnt** | **No Qch** | **No Cbr** |  | 16,23 | 16,96 | 16,71 | 16,77 | 16,37 | 15,93 | 16,29 | 16,39 |
| **Dct** | **No Rnt** | **No Qch** | **No Cbr** |  |  | 0,57 | 0,35 | 0,29 | 0,09 | 0,30 | 0,23 | 0,23 |
|  |  |  | **Cbr** |  |  |  | 0,23 | 0,32 | 0,49 | 0,88 | 0,29 | 0,31 |
|  |  | **Qch** | **No Cbr** |  |  |  |  | 0,07 | 0,26 | 0,66 | 0,09 | 0,10 |
|  |  |  | **Cbr** |  |  |  |  |  | 0,20 | 0,61 | 0,03 | 0,03 |
|  | **Rnt** | **No Qch** | **No Cbr** |  |  |  |  |  |  | 0,39 | 0,14 | 0,15 |
|  |  |  | **Cbr** |  |  |  |  |  |  |  | 0,51 | 0,53 |
|  |  | **Qch** | **No Cbr** |  |  |  |  |  |  |  |  | 0,00 |
|  |  |  | **Cbr** |  |  |  |  |  |  |  |  |  |

*Table S30: Absolute effect size for a pairwise comparison between NPI configurations. Applying DCT has a large effect on “Total Infected” over not applying DCT.*

### Realistic Behaviour

|  |  |  | **DCT** | **No Dct** | | | | | | | | **Dct** | | | | | | | |
| --- | --- | --- | --- | --- | --- | --- | --- | --- | --- | --- | --- | --- | --- | --- | --- | --- | --- | --- | --- |
|  |  |  | **RNT** | **No Rnt** | | | | **Rnt** | | | | **No Rnt** | | | | **Rnt** | | | |
|  |  |  | **QCH** | **No Qch** | | **Qch** | | **No Qch** | | **Qch** | | **No Qch** | | **Qch** | | **No Qch** | | **Qch** | |
|  |  |  | **CBR** | **No Cbr** | **Cbr** | **No Cbr** | **Cbr** | **No Cbr** | **Cbr** | **No Cbr** | **Cbr** | **No Cbr** | **Cbr** | **No Cbr** | **Cbr** | **No Cbr** | **Cbr** | **No Cbr** | **Cbr** |
| **DCT** | **RNT** | **QCH** | **CBR** |  |  |  |  |  |  |  |  |  |  |  |  |  |  |  |  |
| **No Dct** | **No Rnt** | **No Qch** | **No Cbr** |  | 0,46 | 0,36 | 0,10 | 0,91 | 0,77 | 0,73 | 0,64 | 3,51 | 2,79 | 3,67 | 3,03 | 1,87 | 2,10 | 1,78 | 2,48 |
|  |  |  | **Cbr** |  |  | 0,15 | 0,60 | 1,45 | 1,30 | 1,26 | 1,15 | 3,19 | 2,57 | 3,41 | 2,75 | 1,60 | 1,78 | 1,46 | 2,16 |
|  |  | **Qch** | **No Cbr** |  |  |  | 0,50 | 1,47 | 1,29 | 1,24 | 1,11 | 3,45 | 2,69 | 3,60 | 2,94 | 1,74 | 1,96 | 1,63 | 2,37 |
|  |  |  | **Cbr** |  |  |  |  | 0,87 | 0,72 | 0,68 | 0,58 | 3,70 | 2,88 | 3,81 | 3,17 | 1,97 | 2,23 | 1,90 | 2,64 |
|  | **Rnt** | **No Qch** | **No Cbr** |  |  |  |  |  | 0,15 | 0,16 | 0,24 | 4,38 | 3,28 | 4,33 | 3,73 | 2,45 | 2,83 | 2,50 | 3,30 |
|  |  |  | **Cbr** |  |  |  |  |  |  | 0,02 | 0,10 | 4,26 | 3,21 | 4,24 | 3,64 | 2,37 | 2,73 | 2,40 | 3,18 |
|  |  | **Qch** | **No Cbr** |  |  |  |  |  |  |  | 0,08 | 4,20 | 3,19 | 4,20 | 3,60 | 2,34 | 2,69 | 2,37 | 3,14 |
|  |  |  | **Cbr** |  |  |  |  |  |  |  |  | 4,09 | 3,13 | 4,12 | 3,51 | 2,28 | 2,60 | 2,28 | 3,04 |
| **Dct** | **No Rnt** | **No Qch** | **No Cbr** |  |  |  |  |  |  |  |  |  | 0,39 | 0,71 | 0,06 | 0,86 | 1,08 | 1,37 | 0,88 |
|  |  |  | **Cbr** |  |  |  |  |  |  |  |  |  |  | 0,20 | 0,42 | 1,03 | 1,19 | 1,41 | 1,04 |
|  |  | **Qch** | **No Cbr** |  |  |  |  |  |  |  |  |  |  |  | 0,72 | 1,40 | 1,63 | 1,89 | 1,47 |
|  |  |  | **Cbr** |  |  |  |  |  |  |  |  |  |  |  |  | 0,75 | 0,93 | 1,20 | 0,74 |
|  | **Rnt** | **No Qch** | **No Cbr** |  |  |  |  |  |  |  |  |  |  |  |  |  | 0,09 | 0,33 | 0,11 |
|  |  |  | **Cbr** |  |  |  |  |  |  |  |  |  |  |  |  |  |  | 0,28 | 0,23 |
|  |  | **Qch** | **No Cbr** |  |  |  |  |  |  |  |  |  |  |  |  |  |  |  | 0,52 |
|  |  |  | **Cbr** |  |  |  |  |  |  |  |  |  |  |  |  |  |  |  |  |

*Table S31: Absolute effect size for a pairwise comparison between NPI configurations. Applying DCT has a large effect on “Total Infected” over not applying DCT. The effect continues to be large, although smaller than in the Optimal behaviour scenario.*

## Max. 7-day Hosp. Incidence

### Optimal Behaviour

|  |  |  | **DCT** | **No Dct** | **Dct** | | | | | | | |
| --- | --- | --- | --- | --- | --- | --- | --- | --- | --- | --- | --- | --- |
|  |  |  | **RNT** | **No Rnt** | **No Rnt** | | | | **Rnt** | | | |
|  |  |  | **QCH** | **No Qch** | **No Qch** | | **Qch** | | **No Qch** | | **Qch** | |
|  |  |  | **CBR** | **No Cbr** | **No Cbr** | **Cbr** | **No Cbr** | **Cbr** | **No Cbr** | **Cbr** | **No Cbr** | **Cbr** |
| **DCT** | **RNT** | **QCH** | **CBR** |  |  |  |  |  |  |  |  |  |
| **No Dct** | **No Rnt** | **No Qch** | **No Cbr** |  | 6,49 | 6,69 | 6,14 | 6,14 | 6,80 | 5,59 | 6,14 | 6,49 |
| **Dct** | **No Rnt** | **No Qch** | **No Cbr** |  |  | 0,28 | 0,50 | 0,50 | 0,63 | 0,66 | 0,50 | 0,00 |
|  |  |  | **Cbr** |  |  |  | 0,28 | 0,28 | 0,39 | 0,49 | 0,28 | 0,28 |
|  |  | **Qch** | **No Cbr** |  |  |  |  | 0,00 | 0,00 | 0,22 | 0,00 | 0,50 |
|  |  |  | **Cbr** |  |  |  |  |  | 0,00 | 0,22 | 0,00 | 0,50 |
|  | **Rnt** | **No Qch** | **No Cbr** |  |  |  |  |  |  | 0,26 | 0,00 | 0,63 |
|  |  |  | **Cbr** |  |  |  |  |  |  |  | 0,22 | 0,66 |
|  |  | **Qch** | **No Cbr** |  |  |  |  |  |  |  |  | 0,50 |
|  |  |  | **Cbr** |  |  |  |  |  |  |  |  |  |

*Table S32: Absolute effect size for a pairwise comparison between NPI configurations. Applying DCT has a large effect on “Max. 7-day Hosp. Incidence” over not applying DCT.*

Realistic Behaviour

|  |  |  | **DCT** | **No Dct** | | | | | | | | **Dct** | | | | | | | |
| --- | --- | --- | --- | --- | --- | --- | --- | --- | --- | --- | --- | --- | --- | --- | --- | --- | --- | --- | --- |
|  |  |  | **RNT** | **No Rnt** | | | | **Rnt** | | | | **No Rnt** | | | | **Rnt** | | | |
|  |  |  | **QCH** | **No Qch** | | **Qch** | | **No Qch** | | **Qch** | | **No Qch** | | **Qch** | | **No Qch** | | **Qch** | |
|  |  |  | **CBR** | **No Cbr** | **Cbr** | **No Cbr** | **Cbr** | **No Cbr** | **Cbr** | **No Cbr** | **Cbr** | **No Cbr** | **Cbr** | **No Cbr** | **Cbr** | **No Cbr** | **Cbr** | **No Cbr** | **Cbr** |
| **DCT** | **RNT** | **QCH** | **CBR** |  |  |  |  |  |  |  |  |  |  |  |  |  |  |  |  |
| **No Dct** | **No Rnt** | **No Qch** | **No Cbr** |  | 1,10 | 0,23 | 0,82 | 0,33 | 0,22 | 0,00 | 0,42 | 3,11 | 2,94 | 3,25 | 3,00 | 2,47 | 2,25 | 1,67 | 2,25 |
|  |  |  | **Cbr** |  |  | 0,84 | 0,32 | 1,30 | 1,20 | 1,00 | 0,57 | 1,46 | 1,58 | 1,77 | 1,55 | 1,16 | 0,88 | 0,63 | 0,88 |
|  |  | **Qch** | **No Cbr** |  |  |  | 0,56 | 0,53 | 0,42 | 0,21 | 0,20 | 2,61 | 2,56 | 2,82 | 2,58 | 2,11 | 1,88 | 1,41 | 1,88 |
|  |  |  | **Cbr** |  |  |  |  | 1,05 | 0,95 | 0,74 | 0,30 | 1,96 | 2,00 | 2,23 | 2,00 | 1,56 | 1,29 | 0,94 | 1,29 |
|  | **Rnt** | **No Qch** | **No Cbr** |  |  |  |  |  | 0,10 | 0,30 | 0,67 | 3,03 | 2,94 | 3,20 | 2,98 | 2,52 | 2,32 | 1,81 | 2,32 |
|  |  |  | **Cbr** |  |  |  |  |  |  | 0,20 | 0,57 | 2,91 | 2,84 | 3,09 | 2,87 | 2,42 | 2,21 | 1,72 | 2,21 |
|  |  | **Qch** | **No Cbr** |  |  |  |  |  |  |  | 0,38 | 2,67 | 2,63 | 2,87 | 2,65 | 2,21 | 1,99 | 1,54 | 1,99 |
|  |  |  | **Cbr** |  |  |  |  |  |  |  |  | 2,03 | 2,09 | 2,29 | 2,08 | 1,69 | 1,46 | 1,13 | 1,46 |
| **Dct** | **No Rnt** | **No Qch** | **No Cbr** |  |  |  |  |  |  |  |  |  | 0,39 | 0,57 | 0,28 | 0,13 | 0,57 | 0,53 | 0,57 |
|  |  |  | **Cbr** |  |  |  |  |  |  |  |  |  |  | 0,12 | 0,12 | 0,44 | 0,82 | 0,75 | 0,82 |
|  |  | **Qch** | **No Cbr** |  |  |  |  |  |  |  |  |  |  |  | 0,25 | 0,59 | 1,00 | 0,88 | 1,00 |
|  |  |  | **Cbr** |  |  |  |  |  |  |  |  |  |  |  |  | 0,35 | 0,75 | 0,69 | 0,75 |
|  | **Rnt** | **No Qch** | **No Cbr** |  |  |  |  |  |  |  |  |  |  |  |  |  | 0,35 | 0,38 | 0,35 |
|  |  |  | **Cbr** |  |  |  |  |  |  |  |  |  |  |  |  |  |  | 0,10 | 0,00 |
|  |  | **Qch** | **No Cbr** |  |  |  |  |  |  |  |  |  |  |  |  |  |  |  | 0,10 |
|  |  |  | **Cbr** |  |  |  |  |  |  |  |  |  |  |  |  |  |  |  |  |

*Table S33: Absolute effect size for a pairwise comparison between NPI configurations. Applying DCT has a large effect on “Max. 7-day Hosp. Incidence” over not applying DCT. The effect continues to be large, although smaller than in the Optimal behaviour scenario.*

## Max. 7-day Incidence

### Optimal Behaviour

|  |  |  | **DCT** | **No Dct** | **Dct** | | | | | | | |
| --- | --- | --- | --- | --- | --- | --- | --- | --- | --- | --- | --- | --- |
|  |  |  | **RNT** | **No Rnt** | **No Rnt** | | | | **Rnt** | | | |
|  |  |  | **QCH** | **No Qch** | **No Qch** | | **Qch** | | **No Qch** | | **Qch** | |
|  |  |  | **CBR** | **No Cbr** | **No Cbr** | **Cbr** | **No Cbr** | **Cbr** | **No Cbr** | **Cbr** | **No Cbr** | **Cbr** |
| **DCT** | **RNT** | **QCH** | **CBR** |  |  |  |  |  |  |  |  |  |
| **No Dct** | **No Rnt** | **No Qch** | **No Cbr** |  | 5,74 | 5,80 | 5,95 | 5,92 | 5,84 | 5,72 | 5,82 | 6,02 |
| **Dct** | **No Rnt** | **No Qch** | **No Cbr** |  |  | 0,22 | 0,09 | 0,24 | 0,08 | 0,07 | 0,15 | 0,33 |
|  |  |  | **Cbr** |  |  |  | 0,16 | 0,00 | 0,15 | 0,14 | 0,07 | 0,08 |
|  |  | **Qch** | **No Cbr** |  |  |  |  | 0,18 | 0,00 | 0,00 | 0,09 | 0,29 |
|  |  |  | **Cbr** |  |  |  |  |  | 0,17 | 0,15 | 0,08 | 0,09 |
|  | **Rnt** | **No Qch** | **No Cbr** |  |  |  |  |  |  | 0,00 | 0,08 | 0,26 |
|  |  |  | **Cbr** |  |  |  |  |  |  |  | 0,07 | 0,24 |
|  |  | **Qch** | **No Cbr** |  |  |  |  |  |  |  |  | 0,17 |
|  |  |  | **Cbr** |  |  |  |  |  |  |  |  |  |

*Table S34: Absolute effect size for a pairwise comparison between NPI configurations. Applying DCT has a large effect on “Max. 7-day Incidence” over not applying DCT.*

### Realistic Behaviour

|  |  |  | **DCT** | **No Dct** | | | | | | | | **Dct** | | | | | | | |
| --- | --- | --- | --- | --- | --- | --- | --- | --- | --- | --- | --- | --- | --- | --- | --- | --- | --- | --- | --- |
|  |  |  | **RNT** | **No Rnt** | | | | **Rnt** | | | | **No Rnt** | | | | **Rnt** | | | |
|  |  |  | **QCH** | **No Qch** | | **Qch** | | **No Qch** | | **Qch** | | **No Qch** | | **Qch** | | **No Qch** | | **Qch** | |
|  |  |  | **CBR** | **No Cbr** | **Cbr** | **No Cbr** | **Cbr** | **No Cbr** | **Cbr** | **No Cbr** | **Cbr** | **No Cbr** | **Cbr** | **No Cbr** | **Cbr** | **No Cbr** | **Cbr** | **No Cbr** | **Cbr** |
| **DCT** | **RNT** | **QCH** | **CBR** |  |  |  |  |  |  |  |  |  |  |  |  |  |  |  |  |
| **No Dct** | **No Rnt** | **No Qch** | **No Cbr** |  | 1,47 | 0,43 | 0,64 | 0,18 | 0,33 | 0,19 | 0,27 | 3,07 | 2,67 | 3,17 | 2,71 | 2,13 | 2,85 | 1,95 | 2,29 |
|  |  |  | **Cbr** |  |  | 0,81 | 0,55 | 1,66 | 2,05 | 1,51 | 0,86 | 1,55 | 1,37 | 1,86 | 1,39 | 0,93 | 1,34 | 0,69 | 0,93 |
|  |  | **Qch** | **No Cbr** |  |  |  | 0,20 | 0,59 | 0,75 | 0,57 | 0,11 | 2,07 | 1,90 | 2,30 | 1,92 | 1,50 | 1,90 | 1,31 | 1,54 |
|  |  |  | **Cbr** |  |  |  |  | 0,80 | 0,97 | 0,76 | 0,30 | 1,75 | 1,64 | 2,02 | 1,65 | 1,26 | 1,59 | 1,07 | 1,28 |
|  | **Rnt** | **No Qch** | **No Cbr** |  |  |  |  |  | 0,12 | 0,02 | 0,42 | 3,29 | 2,85 | 3,36 | 2,89 | 2,29 | 3,06 | 2,12 | 2,48 |
|  |  |  | **Cbr** |  |  |  |  |  |  | 0,08 | 0,54 | 4,11 | 3,33 | 3,96 | 3,42 | 2,64 | 3,82 | 2,47 | 2,96 |
|  |  | **Qch** | **No Cbr** |  |  |  |  |  |  |  | 0,41 | 2,89 | 2,60 | 3,04 | 2,63 | 2,13 | 2,70 | 1,96 | 2,25 |
|  |  |  | **Cbr** |  |  |  |  |  |  |  |  | 1,99 | 1,87 | 2,23 | 1,88 | 1,51 | 1,84 | 1,33 | 1,54 |
| **Dct** | **No Rnt** | **No Qch** | **No Cbr** |  |  |  |  |  |  |  |  |  | 0,12 | 0,59 | 0,09 | 0,24 | 0,23 | 0,55 | 0,44 |
|  |  |  | **Cbr** |  |  |  |  |  |  |  |  |  |  | 0,39 | 0,03 | 0,30 | 0,29 | 0,57 | 0,47 |
|  |  | **Qch** | **No Cbr** |  |  |  |  |  |  |  |  |  |  |  | 0,43 | 0,66 | 0,77 | 0,96 | 0,89 |
|  |  |  | **Cbr** |  |  |  |  |  |  |  |  |  |  |  |  | 0,28 | 0,27 | 0,56 | 0,45 |
|  | **Rnt** | **No Qch** | **No Cbr** |  |  |  |  |  |  |  |  |  |  |  |  |  | 0,08 | 0,24 | 0,12 |
|  |  |  | **Cbr** |  |  |  |  |  |  |  |  |  |  |  |  |  |  | 0,38 | 0,25 |
|  |  | **Qch** | **No Cbr** |  |  |  |  |  |  |  |  |  |  |  |  |  |  |  | 0,15 |
|  |  |  | **Cbr** |  |  |  |  |  |  |  |  |  |  |  |  |  |  |  |  |

*Table S35: Absolute effect size for a pairwise comparison between NPI configurations. Applying DCT has a large effect on “Max. 7-day Incidence” over not applying DCT. The effect continues to be large, although smaller than in the Optimal behaviour scenario.*

## Wave Duration

### Optimal Behaviour

|  |  |  | **DCT** | **No Dct** | **Dct** | | | | | | | |
| --- | --- | --- | --- | --- | --- | --- | --- | --- | --- | --- | --- | --- |
|  |  |  | **RNT** | **No Rnt** | **No Rnt** | | | | **Rnt** | | | |
|  |  |  | **QCH** | **No Qch** | **No Qch** | | **Qch** | | **No Qch** | | **Qch** | |
|  |  |  | **CBR** | **No Cbr** | **No Cbr** | **Cbr** | **No Cbr** | **Cbr** | **No Cbr** | **Cbr** | **No Cbr** | **Cbr** |
| **DCT** | **RNT** | **QCH** | **CBR** |  |  |  |  |  |  |  |  |  |
| **No Dct** | **No Rnt** | **No Qch** | **No Cbr** |  | 6,84 | 7,16 | 7,13 | 7,18 | 6,85 | 6,71 | 6,81 | 6,31 |
| **Dct** | **No Rnt** | **No Qch** | **No Cbr** |  |  | 0,79 | 0,58 | 0,57 | 0,35 | 0,00 | 0,33 | 0,09 |
|  |  |  | **Cbr** |  |  |  | 0,26 | 0,33 | 0,36 | 0,72 | 0,36 | 0,46 |
|  |  | **Qch** | **No Cbr** |  |  |  |  | 0,05 | 0,14 | 0,52 | 0,15 | 0,29 |
|  |  |  | **Cbr** |  |  |  |  |  | 0,11 | 0,50 | 0,11 | 0,27 |
|  | **Rnt** | **No Qch** | **No Cbr** |  |  |  |  |  |  | 0,32 | 0,01 | 0,17 |
|  |  |  | **Cbr** |  |  |  |  |  |  |  | 0,31 | 0,09 |
|  |  | **Qch** | **No Cbr** |  |  |  |  |  |  |  |  | 0,16 |
|  |  |  | **Cbr** |  |  |  |  |  |  |  |  |  |

*Table S36: Absolute effect size for a pairwise comparison between NPI configurations. Applying DCT has a large effect on “Wave Duration” over not applying DCT.*

### Realistic Behaviour

|  |  |  | **DCT** | **No Dct** | | | | | | | | **Dct** | | | | | | | |
| --- | --- | --- | --- | --- | --- | --- | --- | --- | --- | --- | --- | --- | --- | --- | --- | --- | --- | --- | --- |
|  |  |  | **RNT** | **No Rnt** | | | | **Rnt** | | | | **No Rnt** | | | | **Rnt** | | | |
|  |  |  | **QCH** | **No Qch** | | **Qch** | | **No Qch** | | **Qch** | | **No Qch** | | **Qch** | | **No Qch** | | **Qch** | |
|  |  |  | **CBR** | **No Cbr** | **Cbr** | **No Cbr** | **Cbr** | **No Cbr** | **Cbr** | **No Cbr** | **Cbr** | **No Cbr** | **Cbr** | **No Cbr** | **Cbr** | **No Cbr** | **Cbr** | **No Cbr** | **Cbr** |
| **DCT** | **RNT** | **QCH** | **CBR** |  |  |  |  |  |  |  |  |  |  |  |  |  |  |  |  |
| **No Dct** | **No Rnt** | **No Qch** | **No Cbr** |  | 0,89 | 0,49 | 0,71 | 0,14 | 0,36 | 0,70 | 0,73 | 0,09 | 0,53 | 0,64 | 0,02 | 0,25 | 0,74 | 0,59 | 1,00 |
|  |  |  | **Cbr** |  |  | 1,35 | 0,14 | 0,87 | 0,58 | 0,27 | 0,25 | 0,61 | 1,06 | 1,22 | 0,65 | 0,44 | 0,03 | 0,26 | 0,18 |
|  |  | **Qch** | **No Cbr** |  |  |  | 1,15 | 0,70 | 0,86 | 1,21 | 1,24 | 0,46 | 0,25 | 0,33 | 0,37 | 0,61 | 1,13 | 1,04 | 1,41 |
|  |  |  | **Cbr** |  |  |  |  | 0,67 | 0,40 | 0,11 | 0,08 | 0,48 | 0,95 | 1,10 | 0,52 | 0,32 | 0,10 | 0,12 | 0,31 |
|  | **Rnt** | **No Qch** | **No Cbr** |  |  |  |  |  | 0,27 | 0,66 | 0,69 | 0,00 | 0,63 | 0,76 | 0,07 | 0,17 | 0,70 | 0,54 | 0,98 |
|  |  |  | **Cbr** |  |  |  |  |  |  | 0,34 | 0,37 | 0,17 | 0,74 | 0,88 | 0,24 | 0,01 | 0,47 | 0,28 | 0,72 |
|  |  | **Qch** | **No Cbr** |  |  |  |  |  |  |  | 0,03 | 0,42 | 0,93 | 1,09 | 0,48 | 0,25 | 0,21 | 0,02 | 0,44 |
|  |  |  | **Cbr** |  |  |  |  |  |  |  |  | 0,45 | 0,95 | 1,11 | 0,50 | 0,27 | 0,18 | 0,05 | 0,42 |
| **Dct** | **No Rnt** | **No Qch** | **No Cbr** |  |  |  |  |  |  |  |  |  | 0,53 | 0,62 | 0,06 | 0,14 | 0,53 | 0,38 | 0,73 |
|  |  |  | **Cbr** |  |  |  |  |  |  |  |  |  |  | 0,04 | 0,48 | 0,64 | 0,98 | 0,88 | 1,14 |
|  |  | **Qch** | **No Cbr** |  |  |  |  |  |  |  |  |  |  |  | 0,56 | 0,74 | 1,12 | 1,02 | 1,30 |
|  |  |  | **Cbr** |  |  |  |  |  |  |  |  |  |  |  |  | 0,19 | 0,57 | 0,43 | 0,77 |
|  | **Rnt** | **No Qch** | **No Cbr** |  |  |  |  |  |  |  |  |  |  |  |  |  | 0,38 | 0,22 | 0,57 |
|  |  |  | **Cbr** |  |  |  |  |  |  |  |  |  |  |  |  |  |  | 0,20 | 0,19 |
|  |  | **Qch** | **No Cbr** |  |  |  |  |  |  |  |  |  |  |  |  |  |  |  | 0,42 |
|  |  |  | **Cbr** |  |  |  |  |  |  |  |  |  |  |  |  |  |  |  |  |

*Table S37: Absolute effect size for a pairwise comparison between NPI configurations. Applying DCT has a large effect on “Wave Duration” over not applying DCT. The effect continues to be large, although smaller than in the Optimal behaviour scenario.*

## Days in Quarantine

### Optimal Behaviour

|  |  |  | **DCT** | **No Dct** | **Dct** | | | | | | | |
| --- | --- | --- | --- | --- | --- | --- | --- | --- | --- | --- | --- | --- |
|  |  |  | **RNT** | **No Rnt** | **No Rnt** | | | | **Rnt** | | | |
|  |  |  | **QCH** | **No Qch** | **No Qch** | | **Qch** | | **No Qch** | | **Qch** | |
|  |  |  | **CBR** | **No Cbr** | **No Cbr** | **Cbr** | **No Cbr** | **Cbr** | **No Cbr** | **Cbr** | **No Cbr** | **Cbr** |
| **DCT** | **RNT** | **QCH** | **CBR** |  |  |  |  |  |  |  |  |  |
| **No Dct** | **No Rnt** | **No Qch** | **No Cbr** |  | 13,96 | 14,22 | 15,27 | 14,34 | 39,40 | 37,22 | 37,05 | 35,68 |
| **Dct** | **No Rnt** | **No Qch** | **No Cbr** |  |  | 0,06 | 0,34 | 0,36 | 21,48 | 20,17 | 20,06 | 19,39 |
|  |  |  | **Cbr** |  |  |  | 0,41 | 0,43 | 22,38 | 20,93 | 20,82 | 20,06 |
|  |  | **Qch** | **No Cbr** |  |  |  |  | 0,04 | 23,68 | 21,95 | 21,82 | 20,90 |
|  |  |  | **Cbr** |  |  |  |  |  | 21,06 | 19,76 | 19,66 | 19,00 |
|  | **Rnt** | **No Qch** | **No Cbr** |  |  |  |  |  |  | 0,40 | 0,45 | 0,16 |
|  |  |  | **Cbr** |  |  |  |  |  |  |  | 0,04 | 0,19 |
|  |  | **Qch** | **No Cbr** |  |  |  |  |  |  |  |  | 0,22 |
|  |  |  | **Cbr** |  |  |  |  |  |  |  |  |  |

*Table S38: Absolute effect size for a pairwise comparison between NPI configurations. Applying DCT has a large effect on “Days in Quarantine” over not applying DCT. Furthermore, we see a large effect of not applying RNT vs applying RNT.*

### Realistic Behaviour

|  |  |  | **DCT** | **No Dct** | | | | | | | | **Dct** | | | | | | | |
| --- | --- | --- | --- | --- | --- | --- | --- | --- | --- | --- | --- | --- | --- | --- | --- | --- | --- | --- | --- |
|  |  |  | **RNT** | **No Rnt** | | | | **Rnt** | | | | **No Rnt** | | | | **Rnt** | | | |
|  |  |  | **QCH** | **No Qch** | | **Qch** | | **No Qch** | | **Qch** | | **No Qch** | | **Qch** | | **No Qch** | | **Qch** | |
|  |  |  | **CBR** | **No Cbr** | **Cbr** | **No Cbr** | **Cbr** | **No Cbr** | **Cbr** | **No Cbr** | **Cbr** | **No Cbr** | **Cbr** | **No Cbr** | **Cbr** | **No Cbr** | **Cbr** | **No Cbr** | **Cbr** |
| **DCT** | **RNT** | **QCH** | **CBR** |  |  |  |  |  |  |  |  |  |  |  |  |  |  |  |  |
| **No Dct** | **No Rnt** | **No Qch** | **No Cbr** |  | 0,18 | 0,39 | 0,07 | 44,62 | 43,63 | 44,79 | 43,21 | 0,08 | 0,50 | 0,86 | 0,02 | 28,41 | 33,64 | 28,97 | 32,94 |
|  |  |  | **Cbr** |  |  | 0,57 | 0,12 | 45,12 | 44,12 | 45,31 | 43,68 | 0,03 | 0,54 | 0,92 | 0,04 | 28,66 | 33,97 | 29,23 | 33,26 |
|  |  | **Qch** | **No Cbr** |  |  |  | 0,48 | 44,05 | 43,08 | 44,22 | 42,66 | 0,19 | 0,41 | 0,73 | 0,14 | 28,03 | 33,18 | 28,58 | 32,50 |
|  |  |  | **Cbr** |  |  |  |  | 48,50 | 47,23 | 48,74 | 46,68 | 0,06 | 0,52 | 0,89 | 0,00 | 29,55 | 35,54 | 30,18 | 34,73 |
|  | **Rnt** | **No Qch** | **No Cbr** |  |  |  |  |  | 0,13 | 0,26 | 0,41 | 9,22 | 7,11 | 10,74 | 10,63 | 3,10 | 4,33 | 3,32 | 4,24 |
|  |  |  | **Cbr** |  |  |  |  |  |  | 0,35 | 0,27 | 9,22 | 7,11 | 10,74 | 10,62 | 3,09 | 4,23 | 3,30 | 4,16 |
|  |  | **Qch** | **No Cbr** |  |  |  |  |  |  |  | 0,63 | 9,20 | 7,09 | 10,72 | 10,61 | 3,04 | 4,26 | 3,25 | 4,17 |
|  |  |  | **Cbr** |  |  |  |  |  |  |  |  | 9,24 | 7,13 | 10,76 | 10,65 | 3,17 | 4,31 | 3,38 | 4,23 |
| **Dct** | **No Rnt** | **No Qch** | **No Cbr** |  |  |  |  |  |  |  |  |  | 0,44 | 0,61 | 0,05 | 8,32 | 8,43 | 8,32 | 8,40 |
|  |  |  | **Cbr** |  |  |  |  |  |  |  |  |  |  | 0,06 | 0,42 | 6,42 | 6,48 | 6,41 | 6,46 |
|  |  | **Qch** | **No Cbr** |  |  |  |  |  |  |  |  |  |  |  | 0,61 | 9,50 | 9,69 | 9,50 | 9,65 |
|  |  |  | **Cbr** |  |  |  |  |  |  |  |  |  |  |  |  | 9,52 | 9,68 | 9,52 | 9,64 |
|  | **Rnt** | **No Qch** | **No Cbr** |  |  |  |  |  |  |  |  |  |  |  |  |  | 0,01 | 0,07 | 0,05 |
|  |  |  | **Cbr** |  |  |  |  |  |  |  |  |  |  |  |  |  |  | 0,09 | 0,08 |
|  |  | **Qch** | **No Cbr** |  |  |  |  |  |  |  |  |  |  |  |  |  |  |  | 0,03 |
|  |  |  | **Cbr** |  |  |  |  |  |  |  |  |  |  |  |  |  |  |  |  |

*Table S39: Absolute effect size for a pairwise comparison between NPI configurations. Applying DCT has a large effect on “Days in Quarantine” over not applying DCT. Furthermore, we see a large effect of not applying RNT vs applying RNT. The effect continues to be large, although smaller than in the Optimal behaviour scenario.*

## Generation Interval

### Optimal Behaviour

|  |  |  | **DCT** | **No Dct** | **Dct** | | | | | | | |
| --- | --- | --- | --- | --- | --- | --- | --- | --- | --- | --- | --- | --- |
|  |  |  | **RNT** | **No Rnt** | **No Rnt** | | | | **Rnt** | | | |
|  |  |  | **QCH** | **No Qch** | **No Qch** | | **Qch** | | **No Qch** | | **Qch** | |
|  |  |  | **CBR** | **No Cbr** | **No Cbr** | **Cbr** | **No Cbr** | **Cbr** | **No Cbr** | **Cbr** | **No Cbr** | **Cbr** |
| **DCT** | **RNT** | **QCH** | **CBR** |  |  |  |  |  |  |  |  |  |
| **No Dct** | **No Rnt** | **No Qch** | **No Cbr** |  | 1,35 | 0,47 | 1,04 | 1,73 | 1,21 | 1,95 | 1,92 | 0,98 |
| **Dct** | **No Rnt** | **No Qch** | **No Cbr** |  |  | 0,56 | 0,13 | 0,46 | 0,10 | 0,38 | 0,29 | 0,36 |
|  |  |  | **Cbr** |  |  |  | 0,40 | 0,94 | 0,46 | 0,92 | 0,85 | 0,26 |
|  |  | **Qch** | **No Cbr** |  |  |  |  | 0,56 | 0,04 | 0,49 | 0,41 | 0,19 |
|  |  |  | **Cbr** |  |  |  |  |  | 0,55 | 0,13 | 0,23 | 0,80 |
|  | **Rnt** | **No Qch** | **No Cbr** |  |  |  |  |  |  | 0,48 | 0,39 | 0,25 |
|  |  |  | **Cbr** |  |  |  |  |  |  |  | 0,11 | 0,77 |
|  |  | **Qch** | **No Cbr** |  |  |  |  |  |  |  |  | 0,69 |
|  |  |  | **Cbr** |  |  |  |  |  |  |  |  |  |

*Table S40: Absolute effect size for a pairwise comparison between NPI configurations. Applying DCT has a large effect on “Generation Interval” over not applying DCT.*

### Realistic Behaviour

|  |  |  | **DCT** | **No Dct** | | | | | | | | **Dct** | | | | | | | |
| --- | --- | --- | --- | --- | --- | --- | --- | --- | --- | --- | --- | --- | --- | --- | --- | --- | --- | --- | --- |
|  |  |  | **RNT** | **No Rnt** | | | | **Rnt** | | | | **No Rnt** | | | | **Rnt** | | | |
|  |  |  | **QCH** | **No Qch** | | **Qch** | | **No Qch** | | **Qch** | | **No Qch** | | **Qch** | | **No Qch** | | **Qch** | |
|  |  |  | **CBR** | **No Cbr** | **Cbr** | **No Cbr** | **Cbr** | **No Cbr** | **Cbr** | **No Cbr** | **Cbr** | **No Cbr** | **Cbr** | **No Cbr** | **Cbr** | **No Cbr** | **Cbr** | **No Cbr** | **Cbr** |
| **DCT** | **RNT** | **QCH** | **CBR** |  |  |  |  |  |  |  |  |  |  |  |  |  |  |  |  |
| **No Dct** | **No Rnt** | **No Qch** | **No Cbr** |  | 0,47 | 0,84 | 0,38 | 0,03 | 0,33 | 0,07 | 0,15 | 0,90 | 0,38 | 0,95 | 0,49 | 0,49 | 0,99 | 0,39 | 0,22 |
|  |  |  | **Cbr** |  |  | 0,31 | 0,01 | 0,38 | 0,10 | 0,55 | 0,48 | 1,15 | 0,65 | 1,23 | 0,76 | 0,81 | 1,26 | 0,69 | 0,11 |
|  |  | **Qch** | **No Cbr** |  |  |  | 0,23 | 0,68 | 0,39 | 0,93 | 0,74 | 1,38 | 0,87 | 1,51 | 0,97 | 1,09 | 1,53 | 0,94 | 0,33 |
|  |  |  | **Cbr** |  |  |  |  | 0,32 | 0,10 | 0,44 | 0,43 | 1,04 | 0,59 | 1,08 | 0,69 | 0,71 | 1,12 | 0,62 | 0,10 |
|  | **Rnt** | **No Qch** | **No Cbr** |  |  |  |  |  | 0,26 | 0,09 | 0,15 | 0,86 | 0,37 | 0,89 | 0,48 | 0,46 | 0,93 | 0,38 | 0,18 |
|  |  |  | **Cbr** |  |  |  |  |  |  | 0,39 | 0,38 | 1,05 | 0,56 | 1,10 | 0,66 | 0,69 | 1,14 | 0,59 | 0,03 |
|  |  | **Qch** | **No Cbr** |  |  |  |  |  |  |  | 0,10 | 0,87 | 0,34 | 0,92 | 0,46 | 0,44 | 0,96 | 0,35 | 0,26 |
|  |  |  | **Cbr** |  |  |  |  |  |  |  |  | 0,67 | 0,22 | 0,66 | 0,33 | 0,27 | 0,71 | 0,21 | 0,29 |
| **Dct** | **No Rnt** | **No Qch** | **No Cbr** |  |  |  |  |  |  |  |  |  | 0,40 | 0,07 | 0,29 | 0,44 | 0,02 | 0,46 | 0,89 |
|  |  |  | **Cbr** |  |  |  |  |  |  |  |  |  |  | 0,36 | 0,10 | 0,01 | 0,40 | 0,03 | 0,47 |
|  |  | **Qch** | **No Cbr** |  |  |  |  |  |  |  |  |  |  |  | 0,25 | 0,41 | 0,06 | 0,43 | 0,90 |
|  |  |  | **Cbr** |  |  |  |  |  |  |  |  |  |  |  |  | 0,10 | 0,29 | 0,13 | 0,56 |
|  | **Rnt** | **No Qch** | **No Cbr** |  |  |  |  |  |  |  |  |  |  |  |  |  | 0,45 | 0,05 | 0,55 |
|  |  |  | **Cbr** |  |  |  |  |  |  |  |  |  |  |  |  |  |  | 0,47 | 0,94 |
|  |  | **Qch** | **No Cbr** |  |  |  |  |  |  |  |  |  |  |  |  |  |  |  | 0,48 |
|  |  |  | **Cbr** |  |  |  |  |  |  |  |  |  |  |  |  |  |  |  |  |

*Table S41: Absolute effect size for a pairwise comparison between NPI configurations. Applying DCT has a large effect on “Generation Interval” over not applying DCT. The effect is not as pronounced as in the Optimal behaviour scenario, with some paintings only having medium effect.*

## Serial Interval

### Optimal Behaviour

|  |  |  | **DCT** | **No Dct** | **Dct** | | | | | | | |
| --- | --- | --- | --- | --- | --- | --- | --- | --- | --- | --- | --- | --- |
|  |  |  | **RNT** | **No Rnt** | **No Rnt** | | | | **Rnt** | | | |
|  |  |  | **QCH** | **No Qch** | **No Qch** | | **Qch** | | **No Qch** | | **Qch** | |
|  |  |  | **CBR** | **No Cbr** | **No Cbr** | **Cbr** | **No Cbr** | **Cbr** | **No Cbr** | **Cbr** | **No Cbr** | **Cbr** |
| **DCT** | **RNT** | **QCH** | **CBR** |  |  |  |  |  |  |  |  |  |
| **No Dct** | **No Rnt** | **No Qch** | **No Cbr** |  | 1,99 | 0,66 | 0,90 | 1,19 | 1,12 | 0,60 | 1,37 | 1,24 |
| **Dct** | **No Rnt** | **No Qch** | **No Cbr** |  |  | 0,79 | 0,40 | 0,33 | 0,65 | 0,94 | 0,19 | 0,57 |
|  |  |  | **Cbr** |  |  |  | 0,28 | 0,41 | 0,21 | 0,09 | 0,54 | 0,29 |
|  |  | **Qch** | **No Cbr** |  |  |  |  | 0,10 | 0,11 | 0,37 | 0,21 | 0,04 |
|  |  |  | **Cbr** |  |  |  |  |  | 0,24 | 0,51 | 0,13 | 0,16 |
|  | **Rnt** | **No Qch** | **No Cbr** |  |  |  |  |  |  | 0,33 | 0,38 | 0,09 |
|  |  |  | **Cbr** |  |  |  |  |  |  |  | 0,65 | 0,41 |
|  |  | **Qch** | **No Cbr** |  |  |  |  |  |  |  |  | 0,30 |
|  |  |  | **Cbr** |  |  |  |  |  |  |  |  |  |

*Table S42: Absolute effect size for a pairwise comparison between NPI configurations. Applying DCT has a large effect on “Serial Interval” over not applying DCT.*

### Realistic Behaviour

|  |  |  | **DCT** | **No Dct** | | | | | | | | **Dct** | | | | | | | |
| --- | --- | --- | --- | --- | --- | --- | --- | --- | --- | --- | --- | --- | --- | --- | --- | --- | --- | --- | --- |
|  |  |  | **RNT** | **No Rnt** | | | | **Rnt** | | | | **No Rnt** | | | | **Rnt** | | | |
|  |  |  | **QCH** | **No Qch** | | **Qch** | | **No Qch** | | **Qch** | | **No Qch** | | **Qch** | | **No Qch** | | **Qch** | |
|  |  |  | **CBR** | **No Cbr** | **Cbr** | **No Cbr** | **Cbr** | **No Cbr** | **Cbr** | **No Cbr** | **Cbr** | **No Cbr** | **Cbr** | **No Cbr** | **Cbr** | **No Cbr** | **Cbr** | **No Cbr** | **Cbr** |
| **DCT** | **RNT** | **QCH** | **CBR** |  |  |  |  |  |  |  |  |  |  |  |  |  |  |  |  |
| **No Dct** | **No Rnt** | **No Qch** | **No Cbr** |  | 0,49 | 0,73 | 0,83 | 0,42 | 0,62 | 0,05 | 0,66 | 0,56 | 0,46 | 0,44 | 0,68 | 0,29 | 0,84 | 0,10 | 0,67 |
|  |  |  | **Cbr** |  |  | 0,15 | 0,23 | 0,15 | 0,04 | 0,48 | 0,07 | 1,04 | 0,82 | 0,90 | 1,06 | 0,79 | 1,33 | 0,40 | 0,16 |
|  |  | **Qch** | **No Cbr** |  |  |  | 0,10 | 0,36 | 0,13 | 0,73 | 0,10 | 1,37 | 0,99 | 1,18 | 1,28 | 1,11 | 1,79 | 0,63 | 0,03 |
|  |  |  | **Cbr** |  |  |  |  | 0,47 | 0,23 | 0,83 | 0,20 | 1,47 | 1,05 | 1,28 | 1,36 | 1,21 | 1,90 | 0,72 | 0,06 |
|  | **Rnt** | **No Qch** | **No Cbr** |  |  |  |  |  | 0,23 | 0,40 | 0,28 | 1,06 | 0,78 | 0,89 | 1,05 | 0,78 | 1,44 | 0,31 | 0,34 |
|  |  |  | **Cbr** |  |  |  |  |  |  | 0,61 | 0,03 | 1,25 | 0,91 | 1,07 | 1,19 | 0,98 | 1,65 | 0,51 | 0,13 |
|  |  | **Qch** | **No Cbr** |  |  |  |  |  |  |  | 0,66 | 0,64 | 0,51 | 0,51 | 0,74 | 0,36 | 0,95 | 0,06 | 0,66 |
|  |  |  | **Cbr** |  |  |  |  |  |  |  |  | 1,32 | 0,94 | 1,13 | 1,24 | 1,05 | 1,75 | 0,56 | 0,11 |
| **Dct** | **No Rnt** | **No Qch** | **No Cbr** |  |  |  |  |  |  |  |  |  | 0,07 | 0,09 | 0,24 | 0,29 | 0,23 | 0,66 | 1,23 |
|  |  |  | **Cbr** |  |  |  |  |  |  |  |  |  |  | 0,13 | 0,13 | 0,27 | 0,09 | 0,53 | 0,95 |
|  |  | **Qch** | **No Cbr** |  |  |  |  |  |  |  |  |  |  |  | 0,30 | 0,18 | 0,32 | 0,54 | 1,08 |
|  |  |  | **Cbr** |  |  |  |  |  |  |  |  |  |  |  |  | 0,47 | 0,07 | 0,75 | 1,21 |
|  | **Rnt** | **No Qch** | **No Cbr** |  |  |  |  |  |  |  |  |  |  |  |  |  | 0,57 | 0,39 | 0,99 |
|  |  |  | **Cbr** |  |  |  |  |  |  |  |  |  |  |  |  |  |  | 0,95 | 1,56 |
|  |  | **Qch** | **No Cbr** |  |  |  |  |  |  |  |  |  |  |  |  |  |  |  | 0,58 |
|  |  |  | **Cbr** |  |  |  |  |  |  |  |  |  |  |  |  |  |  |  |  |

*Table S43: Absolute effect size for a pairwise comparison between NPI configurations. Applying DCT has a large effect on “Generation Interval” over not applying DCT. The effect is not as pronounced as in the Optimal behaviour scenario, with some paintings only having a small effect.*

# References

1.	Statista. Households by size in Germany 2020. Statista. 2022. Available from: https://www.statista.com/statistics/464187/households-by-size-germany/ [accessed June 1, 2022]

2.	Vaizman Y, Ellis K, Lanckriet G. Recognizing Detailed Human Context in the Wild from Smartphones and Smartwatches. IEEE Pervasive Comput 2017 Oct;16(4):62–74. doi: 10.1109/MPRV.2017.3971131

3.	Yuan H, Chan S, Creagh AP, Tong C, Acquah A, Clifton DA, Doherty A. Self-supervised learning for human activity recognition using 700,000 person-days of wearable data. Npj Digit Med Nature Publishing Group; 2024 Apr 12;7(1):1–10. doi: 10.1038/s41746-024-01062-3

4.	VGN - Zahlen, Daten, Fakten. VGN. 2020. Available from: https://www.vgn.de/ueber-uns/fakten/ [accessed Sept 24, 2020]

5.	Tomori DV, Rübsamen N, Berger T, Scholz S, Walde J, Wittenberg I, Lange B, Kuhlmann A, Horn J, Mikolajczyk R, Jaeger VK, Karch A. Individual social contact data and population mobility data as early markers of SARS-CoV-2 transmission dynamics during the first wave in Germany—an analysis based on the COVIMOD study. BMC Med 2021 Oct 14;19(1):271. PMID:34649541

6.	Ott D. Corona: Rechtsgrundlagen. Bürgerbeauftragter Bayer Staatsregierung. 2022. Available from: https://www.buergerbeauftragter.bayern/corona-rechtsgrundlagen/ [accessed May 24, 2022]

7.	Kissler SM, Fauver JR, Mack C, Tai CG, Breban MI, Watkins AE, Samant RM, Anderson DJ, Metti J, Khullar G, Baits R, MacKay M, Salgado D, Baker T, Dudley JT, Mason CE, Ho DD, Grubaugh ND, Grad YH. Viral Dynamics of SARS-CoV-2 Variants in Vaccinated and Unvaccinated Persons. N Engl J Med 2021 Dec 23;385(26):2489–2491. doi: 10.1056/NEJMc2102507

8.	He X, Lau EHY, Wu P, Deng X, Wang J, Hao X, Lau YC, Wong JY, Guan Y, Tan X, Mo X, Chen Y, Liao B, Chen W, Hu F, Zhang Q, Zhong M, Wu Y, Zhao L, Zhang F, Cowling BJ, Li F, Leung GM. Temporal dynamics in viral shedding and transmissibility of COVID-19. Nat Med 2020 May;26(5):672–675. doi: 10.1038/s41591-020-0869-5

9.	Ganyani T, Kremer C, Chen D, Torneri A, Faes C, Wallinga J, Hens N. Estimating the generation interval for coronavirus disease (COVID-19) based on symptom onset data, March 2020. Eurosurveillance 2020 Apr 30;25(17):2000257. doi: 10.2807/1560-7917.ES.2020.25.17.2000257

10.	Lauer SA, Grantz KH, Bi Q, Jones FK, Zheng Q, Meredith HR, Azman AS, Reich NG, Lessler J. The Incubation Period of Coronavirus Disease 2019 (COVID-19) From Publicly Reported Confirmed Cases: Estimation and Application. Ann Intern Med 2020 Mar 10;172(9):577–582. PMID:32150748

11.	Ferguson NM, Cummings DAT, Fraser C, Cajka JC, Cooley PC, Burke DS. Strategies for mitigating an influenza pandemic. Nature Nature Publishing Group; 2006 July;442(7101):448–452. doi: 10.1038/nature04795

12.	Hinch R, Probert WJM, Nurtay A, Kendall M, Wymant C, Hall M, Lythgoe K, Cruz AB, Zhao L, Stewart A, Ferretti L, Montero D, Warren J, Mather N, Abueg M, Wu N, Legat O, Bentley K, Mead T, Van-Vuuren K, Feldner-Busztin D, Ristori T, Finkelstein A, Bonsall DG, Abeler-Dörner L, Fraser C. OpenABM-Covid19—An agent-based model for non-pharmaceutical interventions against COVID-19 including contact tracing. PLOS Comput Biol Public Library of Science; 2021 July 12;17(7):e1009146. doi: 10.1371/journal.pcbi.1009146

13.	Wilcox R. A Robust Nonparametric Measure of Effect Size Based on an Analog of Cohen’s d, Plus Inferences About the Median of the Typical Difference. J Mod Appl Stat Methods 2018 Nov 1;17. doi: 10.22237/jmasm/1551905677
